# A competing, dual mechanism for catalytic direct benzene hydroxylation from combined experimental-DFT studies†
†Electronic supplementary information (ESI) available: For experimental catalytic details and mechanistic procedures, including GC and GC-MS traces, additional figures (Fig. S1–S25), computational data including Cartesian coordinates and energies of all stationary points reported in the text. See DOI: 10.1039/c7sc02898a


**DOI:** 10.1039/c7sc02898a

**Published:** 2017-10-05

**Authors:** Laia Vilella, Ana Conde, David Balcells, M. Mar Díaz-Requejo, Agustí Lledós, Pedro J. Pérez

**Affiliations:** a Departament de Química , Universitat Autònoma de Barcelona , 08193 Bellaterra , Spain . Email: agusti@klingon.uab.cat; b Laboratorio de Catálisis Homogénea , Unidad Asociada al CSIC , CIQSO-Centro de Investigación en Química Sostenible , Departamento de Química , Universidad de Huelva , 21007 Huelva , Spain . Email: perez@dqcm.uhu.es ; Email: mmdiaz@dqcm.uhu.es; c Hylleraas Quantum Molecular Sciences , Department of Chemistry , University of Oslo , P.O. Box 1033 Blindern , N-0315 Oslo , Norway . Email: david.balcells@kjemi.uio.no

## Abstract

A dual mechanism for direct benzene catalytic hydroxylation is described. Experimental studies and DFT calculations have provided a mechanistic explanation for the acid-free, Tp^*x*^Cu-catalyzed hydroxylation of benzene with hydrogen peroxide (Tp^*x*^ = hydrotrispyrazolylborate ligand). In contrast with other catalytic systems that promote this transformation through Fenton-like pathways, this system operates through a copper-oxyl intermediate that may interact with the arene ring following two different, competitive routes: (a) electrophilic aromatic substitution, with the copper-oxyl species acting as the formal electrophile, and (b) the so-called rebound mechanism, in which the hydrogen is abstracted by the Cu–O moiety prior to the C–O bond formation. Both pathways contribute to the global transformation albeit to different extents, the electrophilic substitution route seeming to be largely favoured.

## Abstract 2

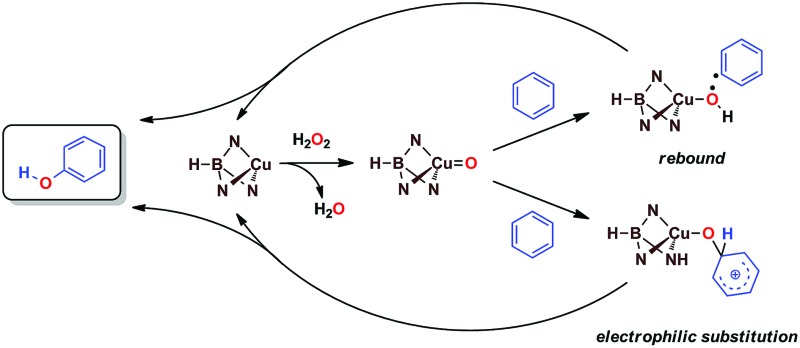
A dual mechanism for direct benzene catalytic hydroxylation is described.

## Introduction

**Scheme 1:**
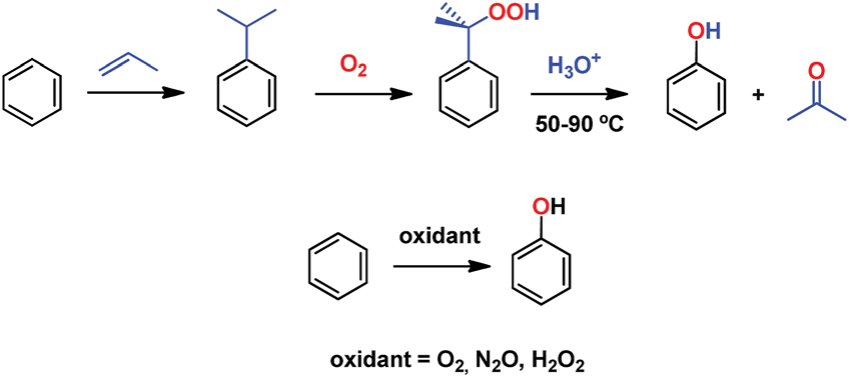
The cumene process (top) and the desired alternative direct hydroxylation of benzene into phenol (bottom).

Phenol is a raw material for producing a large number of chemicals^1^ and it is prepared at the industrial scale by means of the cumene process.^2^ This consists of a three step overall procedure in which benzene and propylene are converted first into cumene, which is further oxidized into cumyl hydroperoxide *en route* to acid-induced decomposition into acetone and phenol (Scheme 1). The instability of the peroxide intermediate as well as the formation of stoichiometric, low-valued acetone are the major drawbacks of this process. One of the most interesting transformations yet to be efficiently developed from a practical point of view is the direct benzene hydroxylation toward phenol, for which a number of systems have been reported.^3^ Several oxidants have been employed, such as molecular oxygen,^4^ dinitrogen oxide^5^ and hydrogen peroxide, the latter being, by far, the most employed in the plethora of contributions described toward that end.^6^ Unfortunately, most of them are far from being practical from an industrial point of view.

**Scheme 2:**
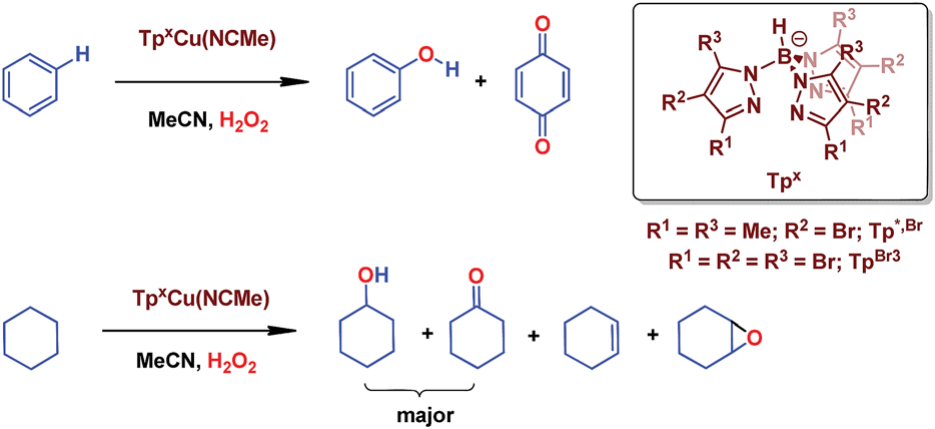
The oxidation of C_sp^2^_–H bonds and C_sp^3^_–H bonds catalysed by Cu(i) complexes bearing trispyrazolyborate ligands.

**Scheme 3:**
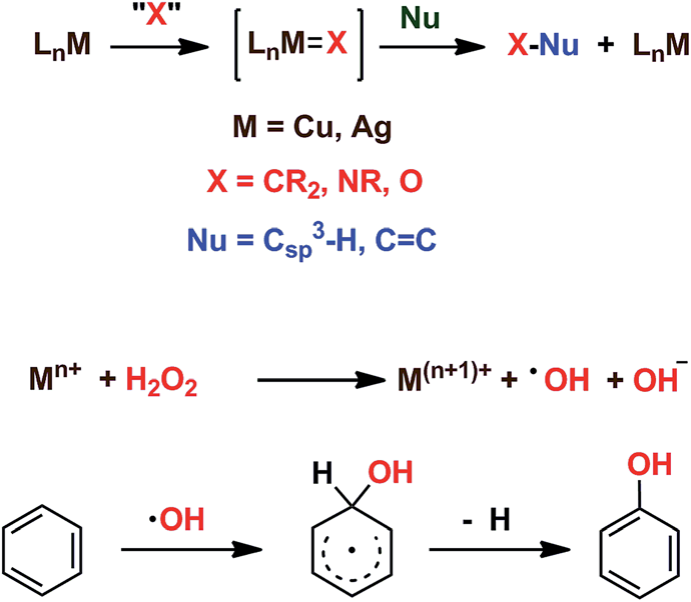
Top: The metal-mediated catalytic transfer of carbene, nitrene and oxo/oxyl groups. Bottom: The Fenton-like oxidation of benzene with hydrogen peroxide.

We have previously reported^7^ a copper-based catalyst containing a trispyrazolylborate type ligand^8^ that induced the hydroxylation of benzene with high selectivity toward phenol (Scheme 2), with H_2_O_2_ as the oxidant and in the absence of acids. Further work from our laboratories also showed the catalytic capabilities of this system to oxidize the C_sp^3^_–H bonds of alkanes as well as to promote their oxidative dehydrogenation (Scheme 2).^9^ We are interested in the study of the mechanisms of these transformations, given our experience with the somewhat related carbene (from diazo compounds)^10^ and nitrene (from iminoiodonanes)^11^ transfer reactions from coinage metal-based catalysts. Those M=X moieties (X = carbene or nitrene, Scheme 3) are well established as intermediates in those functionalization processes. In our alkane oxidation catalytic system,^9^ the presence of the [Tp^*x*^Cu=O] species was also proposed, based on experimental data and theoretical calculations. The latter showed that the electronic structure of this species is closer to a Cu(ii)–O˙ oxyl configuration, with radical character on the oxygen, than to a closed-shell Cu(iii)=O oxo species. On the other hand, the arene oxidation reaction with hydrogen peroxide is well known to proceed through Fenton chemistry,^12^ where the role of the metal center is in the generation of radical species that trigger the C–H oxidation reaction. The generation of hydroxyl radicals (˙OH) capable of oxidizing the arene ring has been known since the last century,^13^ and is still the subject of studies. For instance, a recent contribution by Karlin, Fukuzumi and Yamada^14^ has shown the ability of [Cu(tmpa)]^2+^ (tmpa = tris(2-pyridylmethyl)amine) to induce the direct hydroxylation of benzene by means of generating free HO_2_˙ radicals from H_2_O_2_, that interact with the arene. Herein we report a mechanistic study on the benzene hydroxylation reaction with Tp^*x*^Cu(NCMe) as the catalyst to verify whether this transformation occurs *via* a Fenton-like pathway or through Cu-oxyl species *en route* to oxygen transfer to benzene. Experimental and theoretical data presented in this work have allowed not only to propose the latter, but also the unprecedented feature of the co-existence of two competing pathways from the Cu-oxyl intermediate: an electrophilic aromatic substitution pathway^15^ and the rebound^16^ route, in which a hydrogen atom transfer takes place, the former being more favored in the overall transformation.

## Results and discussion

### Benzene oxidation induced by Tp^*x*^Cu(NCMe): experimental data to evaluate Fenton-like pathways

**Scheme 4:**
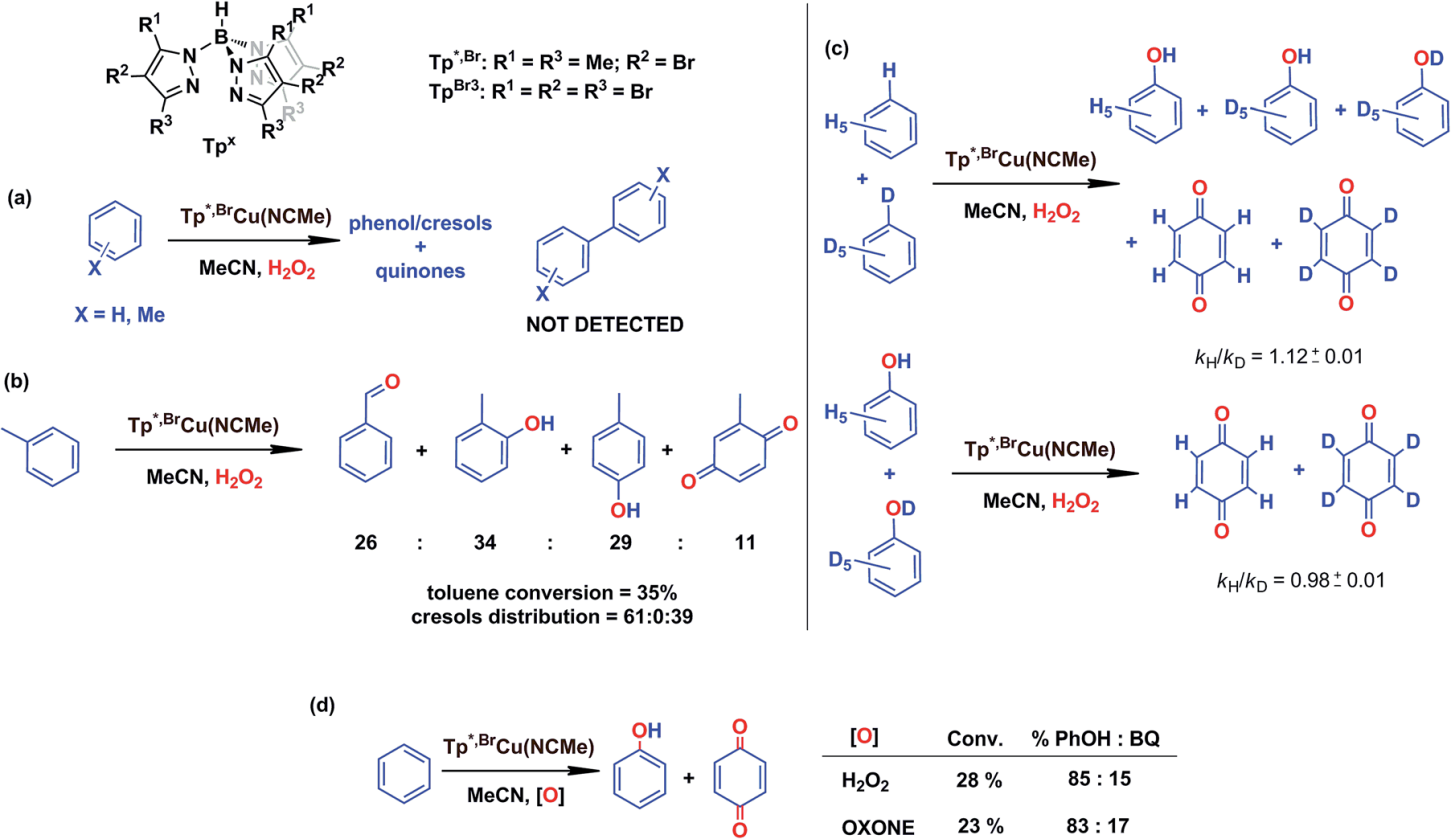
Evidences collected to discard the existence of a Fenton-like mechanism in the Tp^*x*^Cu(NCMe)-catalysed hydroxylation of benzene. Reaction conditions: 0.01 mmol catalyst in 2–3 mL of acetonitrile (see ESI† for details in each case) and 5–10 mmol of the arene (or mixture of protio and deutero benzenes). Reaction time: 5–8 h at 80 °C. H_2_O_2_ is employed in excess compared to the arene.

In our preliminary communication,^7^ the catalytic conversion of benzene into phenol with hydrogen peroxide as oxidant was described to proceed in the presence of complexes of type Tp^*x*^Cu(NCMe) (Scheme 2) and in the absence of acids. For instance, Tp^*,Br^Cu(NCMe) provided 92% selectivity for phenol and 8% for benzoquinone, with sulfolane as an additive (82% for phenol without sulfolane: the use of sulfolane decreases overoxidation of phenol upon protection through hydrogen bond formation) for 25–30% conversion of benzene at 80 °C. Once we had established the viability of these copper-based complexes for the target reaction, we focused on the study of the mechanism of this transformation. We first collected information regarding the possible generation of ˙OH radicals in the reaction mixture containing the Tp^*x*^Cu(NCMe) catalyst, following Fenton chemistry. One typical observation in these reactions is the formation of bis-aryl derivatives, derived from homocoupling of aryl radicals.^13^ In our case, we have not detected, by GC studies with the reaction mixtures, any biaryl compound when using benzene or toluene as substrates (Scheme 4a).

A previous report by Marusawa, Tezuka and co-workers demonstrated^17^ that the distribution of cresols formed upon photolytically generated ˙OH radicals and toluene was 71 : 9 : 20 for *o* : *m* : *p*-isomers, respectively. We have previously reported^9^ that the oxidation of toluene with Tp^*x*^Cu(NCMe) yielded a ratio of 61 : 0 : 39 (accounting the benzoquinone derivatives from their respective cresol precursors, Scheme 4b), a somewhat distinct ratio from that based on the involvement of ˙OH radicals.

A third piece of information comes from competition experiments with protio- and deutero-benzenes (Scheme 4c), from which a relatively low kinetic isotopic effect *k*_H_/*k*_D_ could be obtained as 1.12 ± 0.01. The *k*_H_/*k*_D_ for the overoxidation reaction of a mixture of phenol-H_6_ and phenol-D_6_ has also been determined as 0.98 ± 0.01, thus the above value is not influenced by the subsequent oxidation reaction. For Fenton-type hydroxylations, KIE values of *ca.* 1.7 are frequently reported.^18^

In an effort to check the involvement of radical species, a series of experiments were run in the presence of a radical trap. As shown in Table 1, the experiment in the absence of such additives gave 8.5% benzene conversion with *ca.* 70% selectivity toward phenol. The presence of CCl_4_ or CBrCl_3_ did not affect such results, since both benzene conversions and phenol selectivity were found within a narrow interval in all cases. However, within the total amount of oxygenated products, *ca.* 5% of chloro- or bromobenzene was formed, compared to 95% of phenol and benzoquinone (entries 2 and 3 in Table 1) suggesting the formation of the C_6_H_5_˙ radical^19^ at any stage of the catalytic process (blank experiments in the absence of catalyst did not show such amounts of halobenzenes). In the cyclohexane oxidation with these catalysts,^9^ we found nearly complete trapping of the corresponding cyclohexyl radical (97% of the products). From this difference, it seems that the main path in the benzene hydroxylation reaction is not that based on the generation of phenyl radicals, albeit their presence in the reaction mixture must be explained when proposing a mechanistic picture. The use of radical inhibitors such as TEMPO or 2,6-di-*tert*-butyl-4-methylphenol (BHT) was excluded since they were also oxidized under the reaction conditions, due to the presence of aromatic or aliphatic C–H bonds.

**Table 1:** Effect of additives in the reaction of benzene and H_2_O_2_ using Tp^*,Br^Cu(NCMe) as the catalyst^*a*^

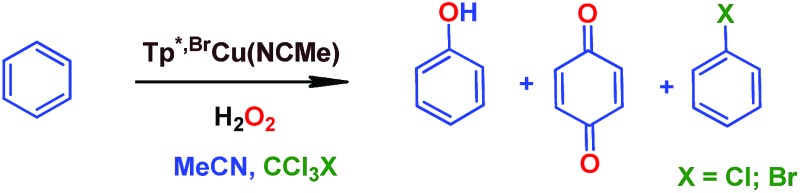			
Entry	Additive	% conversion^*b*^ ^,^^*c*^	PhOH : BQ : Ph–X^*b*^ ^,^^*d*^
1	None	8.5	6% : 2.5% : 0 (70 : 30 : 0)
2	CCl_4_	9	6% : 2.5% : 0.5% (67 : 28 : 5)
3	CBrCl_3_	8.5	6.8% : 1.3% : 0.4%^*e*^ (80 : 15 : 5)

^*a*^Reaction conditions: catalyst, 0.01 mmol; C_6_H_6_, 1 mmol; H_2_O_2_, 1.5 mmol; temp = 80 °C; time = 4 h; 2.5 mL of CH_3_CN; 0.5 mL of radical trap.

^*b*^Values determined by GC using cycloheptanone as internal standard.

^*c*^As percentage of initial benzene consumed.

^*d*^Yields and ratio of products.

^*e*^ *o*-Br-phenol is also detected in *ca.* 8% from oxidation of the bromobenzene formed (see ESI, Fig. S2 and S3).

The use of Oxone®, an oxidant based on KHSO_5_, has led to a similar result in the reaction of benzene and such reactants in the presence of catalytic amounts of Tp^*,Br^Cu(NCMe) (Scheme 4d). It is generally accepted that no radicals are involved in such an oxidation, with a metalloxo intermediate being usually proposed.^20^

### Effect of substituents in the benzene ring in the oxidation reactions

**Fig. 1:**
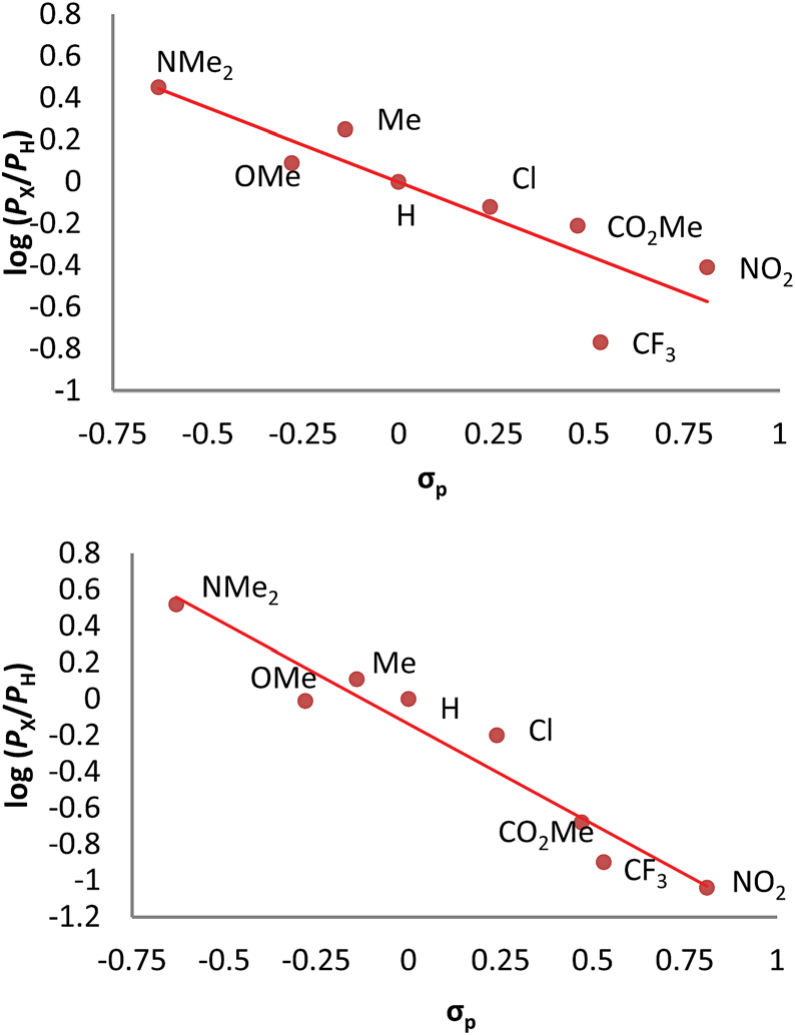
Hammett *σρ* correlations of mono-substituted benzene hydroxylations with Tp^*,Br^Cu(NCMe) (top) and Tp^Br3^Cu(NCMe) (bottom). *P*_X_ corresponds to the yields of hydroxylated products (and their overoxidation derivatives) of substituted benzenes and *P*_H_ is the yield with benzene as the substrate. See Fig. S5–S12 in the ESI† for a complete description.

We have also carried out a series of experiments with several substituted benzenes bearing electron-withdrawing or electron-donating groups, to assess their effect on the C_sp^2^_–H bond oxidation reaction catalyzed by Tp^*x*^Cu(NCMe) complexes, with hydrogen peroxide as the oxidant. When R-C_6_H_5_ rings were employed as substrates, mixtures of products were obtained (Table 2), in most cases consisting of the three X-substituted phenols (in different ratios) as well as the *ortho*-substituted benzoquinone. With toluene, as previously shown (Scheme 4b), the product derived from the oxidation of the methyl group was also observed. The study was performed with two copper complexes as catalysts, Tp^*,Br^Cu(NCMe) and Tp^Br3^Cu(NCMe). Table 2 displays the values of the yields of each product, from which a general trend is extracted: arenes with electron-donating groups as substituents are more prone to react than those bearing electron-withdrawing groups. We have employed those values of reactivity to build Hammett-like plots (Fig. 1), albeit the data do not correspond to competition experiments, that due to the very low conversions observed (<5%), do not provide good quality data toward such a purpose. Thus, the ratio *P*_x_/*P*_H_ corresponds to the yields of all hydroxylation products with X-substituted benzenes *vs.* that of benzene, that of benzene, in what we consider an estimation of the relative reactivity of the whole arene. The *ρ* values obtained from fitting were –0.75 and –1.1 for Tp^*,Br^Cu(NCMe) and Tp^Br3^Cu(NCMe), respectively. With the above restrictions in mind, we interpret the negative value as the consequence of an electrophilic intermediate with a certain positive charge that is respectively stabilised or destabilised with donor or acceptor groups in the benzene ring. Visser, Nam and co-workers have described^21^ negative *ρ* values for the aromatic hydroxylation reaction where an oxo-iron intermediate is involved. Although they found a much larger effect of the substituents (*ρ* = –3.9), we believe that our data (which are not obtained from pure competition experiments from substrates), in conjunction with the other pieces of information, point toward fitting in the aromatic electrophilic substitution mechanism, where a copper-oxyl species would act as an electrophile attacking the nucleophilic arene ring.

**Table 2:** Effect of substituents in the benzene ring on the oxidation reactions using Tp^*,Br^Cu(NCMe) and Tp^Br3^Cu(NCMe) as catalysts^*a*^

R	Conversion^*b*^ ^,^^*c*^	P1%^*b*^	P2%^*b*^	P3%^*b*^	P4%^*b*^
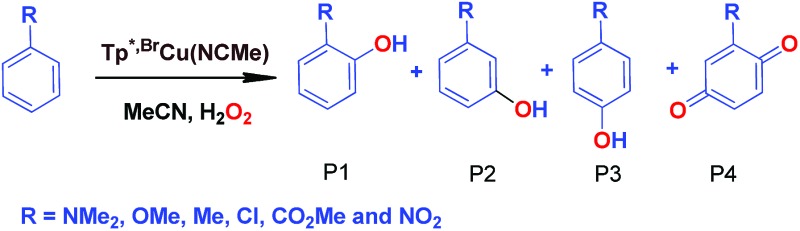					
NMe_2_	22%	73	—	27	—
OMe	9.5%	72	7%	18.6	2.4
Me^*d*^	14%	31.2	—	20.6	23.2
Cl	5.9%	38	—	38	24
CO_2_Me	4.8%	27.5	31	41.5	—
CF_3_	1.4%	8.5	52.4	39.1	—
NO_2_	3%	19	28	53	—

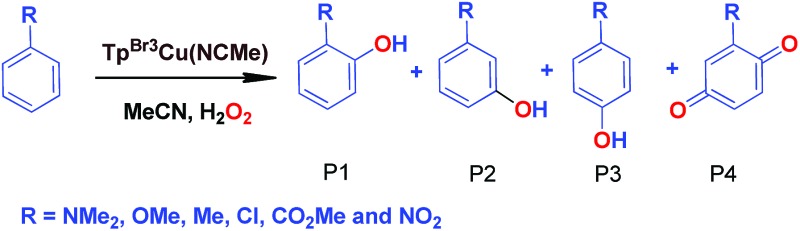					
NMe_2_	31.5%	48	—	52	—
OMe	9.33%	61	12.3	16	10.7
Me^*d*^	12.5%	22.7	—	17.8	34.5
Cl	6.03%	29	—	32	39
CO_2_Me	1.96%	18	36	46	—
CF_3_	1.2%	9.8	60.5	29.7	—
NO_2_	0.9%	15	41	44	—

^*a*^Reaction conditions: catalyst, 0.01 mmol; substrate 1 mmol; H_2_O_2_, 1.5 mmol; temp = 80 °C; time = 4 h; 3 mL of CH_3_CN.

^*b*^Values determined by GC using cycloheptanone as an internal standard.

^*c*^As percentage of initial benzene consumed.

^*d*^Benzaldehyde is also detected in *ca.* 25% from oxidation of the toluene, for both catalysts.

### The nuclearity of the active species

**Fig. 2:**
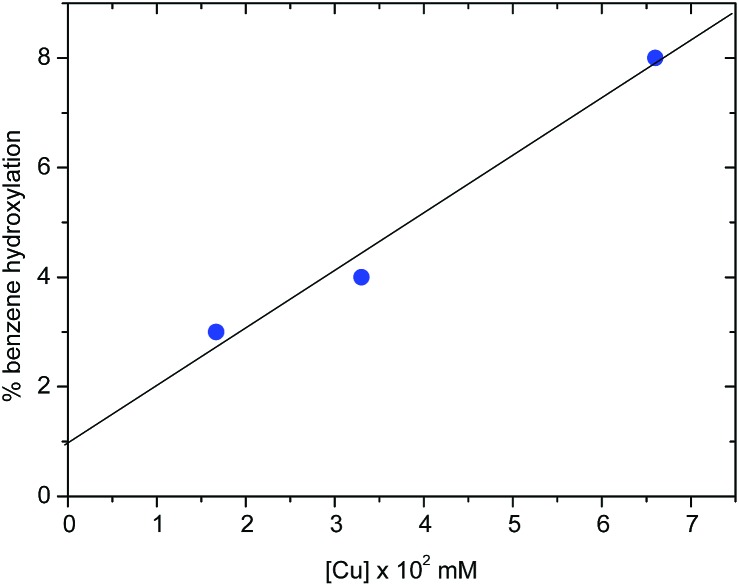
Effect of the catalyst concentration on the overall benzene oxidation reaction (phenol + quinone). Experiments carried out at 60 °C, 30 min, in 3 mL of MeCN, with 3 mmol of benzene and 9 mmol of H_2_O_2_, with Tp^*,Br^Cu(NCMe) as the catalyst.

The activation of dioxygen by copper complexes bearing the trispyrazolylborate ligand has been extensively studied.^22^ Kitajima and co-workers pioneered^23^ this area, showing that two copper centers were involved in the activation of O_2_. In a previous report from one of our laboratories we also found that a series of Tp^*x*^Cu complexes reacted with dioxygen and carbon dioxide in a consecutive sequence leading to dinuclear carbonato-bridged complexes.^24^ Thus, we wondered about a possible implication of dinuclear intermediates in the aromatic hydroxylation reactions described in this contribution, in spite of being performed with hydrogen peroxide, and not with molecular oxygen. A series of catalytic experiments of benzene oxidation were run, varying the concentration of the catalyst [Tp^*,Br^Cu(NCMe)] from 0.0166 mM to 0.0666 mM. As shown in Fig. 2, a linear dependence was observed, assessing a first-order dependence on catalyst concentration. In addition, TOF values of 2.4 h^–1^ were found for the experiments carried out with 3.33 mM and 6.66 mM of catalyst. The observation of this TOF value independent of the concentration of catalyst supports the proposal of a mononuclear species as the catalytically relevant species in this process.

### The mechanism of the benzene hydroxylation reaction from experimental data

**Scheme 5:**
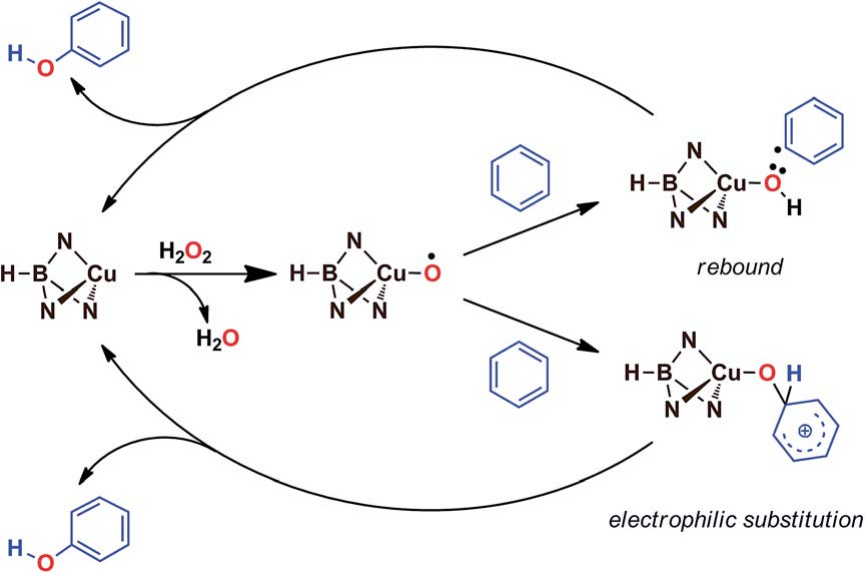
The two pathways leading to the formation of phenol, compatible with experimental data.

Based on the above data, any mechanistic interpretation for the Tp^*x*^Cu-catalyzed hydroxylation of benzene and its derivatives should account for the following: (a) the lack of formation of ˙OH or other H_2_O_2_-derived free radicals; (b) the involvement of copper-oxyl species; (c) the formation of intermediates that resemble the electrophilic aromatic substitution mechanism (based on the effect of the substituents) and (d) the presence of the phenyl radical (albeit at a very low extent). Scheme 5 shows a plausible general picture in agreement with the above requirements. The interaction of the Tp^*x*^Cu core with H_2_O_2_ leads to the Tp^*x*^Cu–O˙ species, which was found to be more feasible than hydroperoxo Tp^*x*^Cu–OOH or superoxo Tp^*x*^Cu(η^2^-O_2_) alternatives.^9^ Such a copper-oxyl intermediate may react with benzene throughout the rebound route in which a hydrogen atom is abstracted from benzene, two radical species being originated. These short-lived radicals collapse upon forming a C–O bond leading to phenol. This path would explain the observation of small amounts of halobenzenes when the reaction is carried out in the presence of CCl_4_ or CBrCl_3_. The second path corresponds to an electrophilic substitution, the copper-oxyl intermediate, electrophilic in nature, attacking the arene ring with the concomitant formation of a Wheland-intermediate, from which phenol is finally delivered.

In view of this dichotomy between two possible paths arisen from experimental data, we decided to study this reaction from a theoretical point of view, as shown in the next section.

### Computational studies: electrophilic aromatic substitution on benzene

**Fig. 3:**
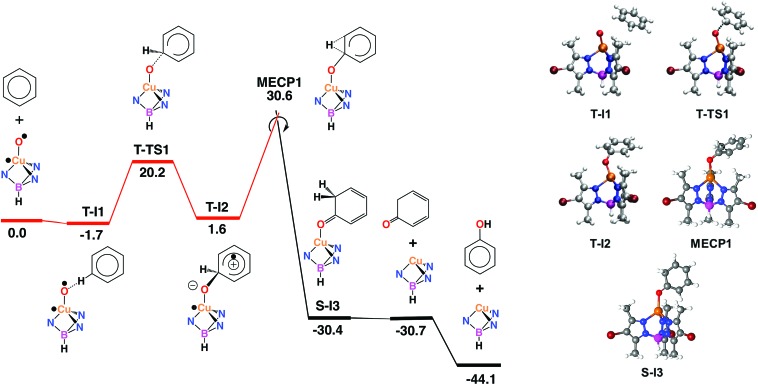
Left: Gibbs energy profile in solution, in kcal mol^–1^, of the electrophilic aromatic substitution (EAS) mechanism with crossing triplet (red) and singlet (black) state reaction pathways. Right: optimized stationary points on the EAS pathway. Color code: orange (Cu), blue (N), red (O), purple (B), maroon (Br), grey (C) and white (H).

With the aim of providing a better understanding of the benzene oxidation process and rationalizing the experimental observations, a computational study of the reaction mechanism was performed at the Density Functional Theory (DFT) level (see ESI† for details). We have first computed the interaction of benzene and the copper-oxyl species Tp^*,Br^Cu–O˙; the reaction pathway is shown in Fig. 3. Once the oxyl is formed from the catalyst and hydrogen peroxide, a process which is exoergic by –3.6 kcal mol^–1^,^9^ the first step, **T-I1** → **T-I2**, involves the addition of the substrate to the oxyl ligand. This reaction takes place in the triplet state, which is the ground state of the copper-oxyl species.^25^,^26^ In the transition state, **T-TS1** (Fig. 3), the oxyl binds to benzene (Table 3), *d*(C···O) = 1.89 Å (3.39 and 1.40 Å in **T-I1** and **T-I2**, respectively), and remains bound to the metal centre, *d*(Cu–O) = 1.80 Å (1.81 Å in **T-I1** and **T-I2**, respectively). The relaxation of **T-TS1** by means of IRC calculations connected this transition state to intermediates **T-I1** (reactant) and **T-I2** (product). In **T-I1**, benzene is associated to the oxyl through a weak hydrogen bond; *d*(CH···O) = 2.58 Å. Due to the entropy penalty, this intermediate is almost isoenergetic with the starting reactants. In **T-I2**, the substrate appears bound to copper through the oxyl. This species can be described as a Wheland intermediate^27^ due to its zwitterionic nature, with the copper-oxyl and benzene fragments negatively and positively charged, respectively; *q* = *q*(C_6_H_6_) = –*q*(Tp^*,Br^CuO) = 0.29. In line with this, electron density is transferred from the C_6_H_6_ fragment to Tp^*,Br^CuO as the system evolves from **T-I1** (*q* = 0.00) to **T-TS1** (*q* = 0.18). This charge redistribution and the nature of **T-I2** are consistent with an electrophilic aromatic substitution (EAS) mechanism, in which the aromatic ring acts as the nucleophile. The loss of aromaticity is reflected on the C_*ipso*_ – C_*ortho*_ distance, which elongates from 1.39 Å (**T-I1**) to 1.50 Å (**T-I2**). The formation of **T-I2** is endoergic, Δ*G* = 1.6 kcal mol^–1^, and involves a significant energy barrier, Δ*G*^‡^ = 20.2 kcal mol^–1^.

**Table 3:** Selected bond distances, in Å, for the species involved in the EAS pathway

Species	Cu–O	C_*ipso*_–O	C_*ipso*_–H	C_*ortho*_–H	C_*ipso*_–C_*ortho*_	C_*ortho*_–O
**T-I1**	1.81	3.39	1.08	2.14	1.39	4.47
**T-TS1**	1.80	1.89	1.08	2.15	1.42	2.56
**T-I2**	1.81	1.40	1.10	2.10	1.50	2.38
**MECP1**	1.97	1.30	1.19	1.83	1.47	2.40
**S-I3**	2.06	1.23	2.11	1.09	1.51	2.36

**Fig. 4:**
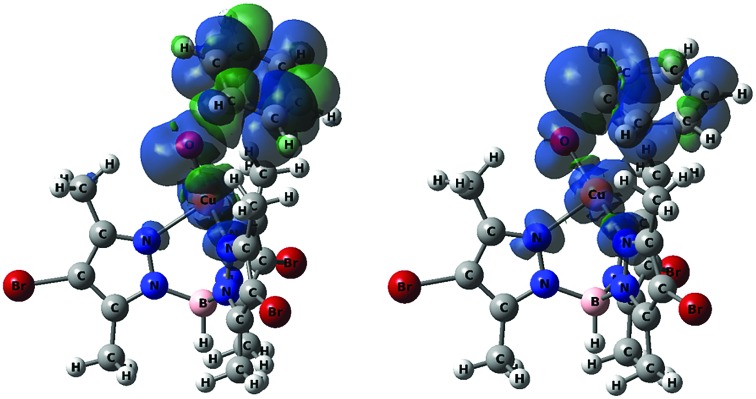
Spin density plots of **T-TS1** (left) and **T-TS3** (right), both plotted at isovalue = 0.001. Color code: blue (α), and green (β).

The data given in Table 4 reflect the spin density redistribution along the triplet state stage of the reaction pathway. In the initial addition step, the evolution of the local spin densities from **T-I1** (*ρ*(O) = 1.11, *ρ*(C_6_H_6_) = 0.00) to **T-I2** (*ρ*(O) = 0.14, *ρ*(C_6_H_6_) = 0.95) shows that one of the π electrons of benzene pairs with the single electron on oxygen (oxyl) yielding the C–O bond. The spin density plot of the addition transition state, **T-TS1** (Fig. 4), shows the pairing of α and β electron densities along the incipient C···O bond. The metal centre does not participate in this electron rearrangement, as shown by the high but invariant values of *ρ*(Cu) on **T-I1**, **T-TS1** and **T-I2**. A similar role is played by the Tp^*,Br^ ligand, which has low but also invariant *ρ* values, consistent with a redox-innocent behaviour.

**Table 4:** Selected local charges (*q*) and spin densities (*ρ*), in a.u. for the species involved in the EAS pathway^*a*^

Species	|*q*|^*b*^	*ρ*(Cu)	*ρ*(O)	*ρ*(C_6_H_6_)	*ρ*(Tp^*,Br^)
**T-I1**	0.00	0.79	1.11	0.00	0.10
**T-TS1**	0.18	0.78	0.78	0.36	0.08
**T-I2**	0.29	0.81	0.14	0.95	0.10
**S-I3**	0.74	0.00	0.00	0.00	0.00

^*a*^ *q* and *ρ* are not given for **MECP1** due to different values in the singlet and triplet states.

^*b*^Absolute values of the local charges of the C_6_H_6_ (>0) and CuOTp^*,Br^ (<0) fragments; *q*(C_6_H_6_) = –*q*(CuOTp^*,Br^).

The generation of phenol from **T-I2** (Fig. 3) requires transferring the hydrogen atom from the *ipso* carbon to oxygen. Despite numerous attempts, no transition state could be located for this process. In contrast, the re-optimization of **T-I2** in the singlet state yielded cyclohexadienone as an oxidation product. This suggested that triplet to singlet spin-crossover, which is needed to recover the Cu(i) catalyst in its ground state, may play a key role by triggering the oxidation of the substrate. The minimum energy crossing point (MECP) associated with this process, MECP1 (Fig. 3), was optimized. This point of the potential energy surface has the distinct property of having the same energy in the singlet and triplet states. The full optimization of MECP1 in the triplet and singlet states yielded intermediates **T-I2** and **S-I3**, respectively. Single point calculations revealed that these species are higher in energy in the singlet (47.9, closed-shell, and 53.7, open-shell, kcal mol^–1^ for S-I2) and triplet (57.9, open-shell, kcal mol^–1^ for T-I3) states.

The comparison of MECP1 to **T-I2** revealed two interesting features in the former geometry; *i.e.* the C–O bond is shorter, 1.30 Å (1.40 Å in **T-I2**), and the H bound to the *ipso* C is shifted towards the *ortho* C, *d*(C–H) = 1.19 and 1.83 Å (1.10 and 2.10 Å in **T-I2**). The flow of electron density along this spin crossover process takes place from the organic fragment to the metal complex, *q* = *q*(C_6_H_6_) = –*q*(Tp^*,Br^CuO) = 0.29 (**T-I2**) and 0.74 (**S-I3**), which is consistent with an EAS mechanism.^28^ MECP1 lies at a rather high energy level, 30.6 kcal mol^–1^ above reactants. This energy may be lowered by factors not considered in this model due to their complexity, including tunneling and explicit solvation of the H transferred. Furthermore, the low conversions observed experimentally in the oxidation of benzene are consistent with high-energy reaction pathways.

The optimization of **MECP1** yielded **T-I2** in the triplet state and **S-I3** in the singlet state (Fig. 3). In **S-I3**, 1,3-cyclohexadienone is formed and coordinated to copper in a η^1^-O fashion, *d*(Cu–O) = 2.06 and *d*(C–O) = 1.23 Å (Table 4). Triplet-to-singlet spin crossover is strongly exoergic, with **S-I3** lying 30.4 kcal mol^–1^ below the reactants (Fig. 3). Cyclohexadienone decoordination and isomerization to phenol make the overall reaction exoergic by 44.1 kcal mol^–1^. The keto–enol tautomerization taking place in the last step has been well characterized experimentally^29^ as an extremely fast and exoergic process in polar solvents.

It is noteworthy to mention that we also explored the activity of superoxo (Fig. S14†) and hydroperoxo species (Fig. S15†) in the benzene oxidation. The barriers associated to benzene oxidation by the superoxo (32.8 and 41.4 kcal mol^–1^) and hydroperoxo (47.3 kcal mol^–1^) species are all higher than that of the oxyl species (30.6 kcal mol^–1^; Fig. 3). These results suggest that the superoxo and hydroperoxo complexes are less reactive in the C–H oxidation of benzene, which is in agreement with similar systems previously reported.^30^ In this regard, the potential role of ˙OOH radicals in yielding active copper-hydroperoxo species was excluded.

### Electrophilic aromatic substitution on the substituted benzenes

The EAS mechanism was fully recomputed for the *para*-substituted benzenes, Ph–X (X = CF_3_, NO_2_, Cl, Me, OMe and NMe_2_). The energy profiles (see ESI†) showed that the most demanding step is the triplet-to-singlet spin crossover, as in the case of benzene (**T-I2** → **MECP1** → **S-I3**), except for –NMe_2_. The energy barrier associated with the MECP (Table 5), Δ*G*_MECP1_, is very sensitive to the nature of the substituent, ranging from a minimum of 18.8 kcal mol^–1^ (X = NMe_2_) to a maximum of 33.4 kcal mol^–1^ (X = CF_3_). Furthermore, for X = OMe and NMe_2_, we also computed the EAS mechanism for the *ortho* and *meta* positions. In both cases, the *ortho* gave the lowest energy **MECP1**, though with a very small difference compared to the *meta* and *para*, in line with the composition of the product mixtures observed experimentally. These results also showed that the relative orientation of the electrophile does not have a significant impact on the mechanism.

**Table 5:** Energies, in kcal mol^–1^, and selected bond distances, in Å, for **MECP1** in the EAS pathway with *para*-substituted Ph–X

X	Δ*G*_MECP1_	Cu–O	C_*ipso*_–O	C_*ipso*_–H	C_*ortho*_–H	C_*ipso*_–C_*ortho*_	C_*ortho*_–O
–NMe_2_	18.8^*a*^	1.95	1.35	1.12	2.05	1.49	2.38
–OMe	21.1^*b*^	1.98	1.32	1.15	1.93	1.48	2.41
–Me	27.4	1.94	1.41	1.09	2.18	1.47	1.91
–Cl	29.2	1.94	1.41	1.09	2.19	1.47	1.86
–CF_3_	33.4	1.93	1.42	1.09	2.19	1.47	1.83
–NO_2_	31.6	1.92	1.29	1.28	1.45	1.46	2.42

^*a*^17.3 (*ortho*) and 19.6 (*meta*) kcal mol^–1^.

^*b*^21.0 (*ortho*) and 22.8 (*meta*) kcal mol^–1^.

The apparent trend is that the reaction is accelerated by electron-donating groups (Δ*G*_MECP1_ < 28 kcal mol^–1^ with X = Me, OMe and NMe_2_) and slowed down by electron-withdrawing groups (Δ*G*_MECP1_ > 28 kcal mol^–1^ with X = Cl, NO_2_ and CF_3_). Interestingly, this signature feature of the EAS mechanism is preserved despite of the unusual two-spin-state nature of the reaction pathway found in this system. The observation of this trend in both the experiments and the calculations supports the EAS as the mechanism operating in this reaction.

The structure of **MECP1** also depends strongly on the nature of the substituent X (Table 5). With X = NO_2_, OMe and NMe_2_, the spin crossover triggers the same chemical transformations observed for benzene (Fig. 3), *i.e.* H migration from the *ipso* to the *ortho* C yielding 1,3-cyclohexadienone. In contrast, with X = Me, Cl and CF_3_, the oxygen atom adds to the *ortho* C yielding benzene oxide, which is another tautomer of phenol,^31^ (Fig. S22†); *e.g.* with X = CF_3_, *d*(C_*ipso*_–O) = 1.42 Å and *d*(C_*ortho*_–O) = 1.83 Å. Nonetheless, these benzene oxides are much less stable than their 1,3-cyclohexadienone isomers, by *ca.* 30 kcal mol^–1^, and it is well known from the literature^32^ that they rapidly isomerize to phenols through dienone intermediates. Thus, for the sake of comparison, the energies of the **S-I3** intermediate with X = Me, Cl and CF_3_ (see ESI†) correspond to the ketone form. The Wheland-type intermediate found for benzene, **T-I2**, is also formed with all substituted Ph–X substrates. The local charges and spin densities on the C_6_H_5_X fragment (Table S1†) show its radical-cation nature, with values ranging from 0.26 to 0.30 (*q*) and from 0.93 to 0.97 (*ρ*). Like benzene, the formation of **T-I2** is moderately endoergic for most X substituents, as shown by the Gibbs energy of this intermediate relative to reactants, *G*_T-I2_.

### Oxygen rebound on benzene

**Fig. 5:**
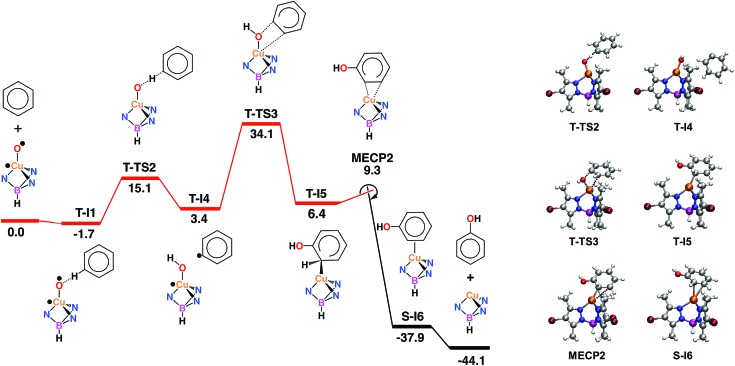
Left: Gibbs energy profile in solution, in kcal mol^–1^, of the rebound mechanism with crossing triplet (red) and singlet (black) state reaction pathways. Right: optimized stationary points on the rebound pathway. Color code: orange (Cu), blue (N), red (O), purple (B), maroon (Br), grey (C) and white (H).

The radical oxyl character of the Cu–O moiety in **T-1** (*ρ*(O) = 1.11, Table 4) suggests that benzene may also be oxidized through a rebound mechanism initiated by H abstraction.^33^ The calculations showed that the oxyl abstracts one H from benzene by following a single-step pathway in the triplet state, **T-1** → **T-TS2** → **T-4** (Fig. 5). C–H cleavage in the transition state **T-TS2** (*d*(C_*ipso*_···H) = 1.32 Å, *d*(O···H) = 1.14 Å; Table 6) yields a phenyl fragment, C_6_H_5_, which is weakly bound to a hydroxo complex, [Tp^*,Br^Cu(OH)], in intermediate **T-I4** (*d*(O–H) = 0.95 Å). The C_6_H_5_ fragment in **T-I4** is well-defined as a neutral phenyl radical by its local charge, *q*(C_6_H_5_) = –0.01, and spin density, *ρ*(C_6_H_5_) = 1.00 (Table 7). The values of *ρ*(O) and *ρ*(C_6_H_5_) in **T-TS2**, 0.62 and 0.56, respectively, show the homolytic nature of the C–H cleavage, with a single electron being transferred from the phenyl ring to the oxyl. Conversely, *ρ* values for copper and the Tp^*,Br^ ligand are almost invariant, thus showing their spectator role in this reaction. The Δ*G* for H abstraction, 15.1 kcal mol^–1^, suggests that the phenyl radical is thermally accessible, in line with the Ph–Cl and Ph–Br products observed experimentally in the presence of halogenation reagents. Nonetheless, the endoergic nature of this step undermines the possibility of having free diffusing radicals at concentrations allowing for the formation of homocoupling products, which are not observed in the experiments. This step is more endoergic than the addition of benzene to the Cu-oxyl moiety in the EAS mechanism (Δ*G* = 3.3 kcal mol^–1^; Fig. 3). In contrast, the energy barrier, Δ*G*^‡^ = 16.8 kcal mol^–1^, is significantly lower than that of the addition (Δ*G*^‡^ = 21.9 kcal mol^–1^).

**Table 6:** Selected bond distances, in Å, for the species involved in the rebound pathway

Species	Cu–O	C_*ipso*_–H	O–H	C_*ipso*_–O	Cu–C_*ortho*_	Cu–C_*meta*_
**T-I1**	1.81	1.08	2.58	3.39	4.13	5.12
**T-TS2**	1.81	1.32	1.14	2.46	4.04	5.38
**T-I4**	1.81	4.09	0.95	4.39	6.56	6.67
**T-TS3**	1.91	2.30	0.96	1.79	2.44	3.33
**T-I5**	3.14	1.91	0.95	1.37	2.03	2.95
**MECP2**	2.87	1.92	0.96	1.38	1.98	2.99
**S-I6**	3.90	1.91	0.95	1.35	2.36	2.49

**Table 7:** Selected local charges (*q*) and spin densities (*ρ*), in a.u., for the species involved in the rebound pathway^*a*^

Species	*q*(Cu)	*q*(O)	*q*(C_6_H_5_)	*ρ*(Cu)	*ρ*(O)	*ρ*(C_6_H_5_)	*ρ*(Tp^*,Br^)
**T-I1**	0.79	1.111	0.00	0.79	1.11	0.00	0.10
**T-TS2**	0.80	0.62	0.56	0.80	0.62	0.56	0.09
**T-I4**	0.83	0.09	1.00	0.83	0.09	1.00	0.08
**T-TS3**	0.83	0.17	0.91	0.83	0.17	0.91	0.08
**T-I5**	1.00	0.03	0.90	1.00	0.03	0.90	0.07
**S-I6**	0.00	0.00	0.00	0.00	0.00	0.00	0.00

^*a*^ *q* and *ρ* are not given for **MECP1** due to different values in the singlet and triplet states.

Hydrogen abstraction is followed by the rebound of the phenyl radical to the hydroxo ligand, **T-I4** → **T-TS3** → **T-I5** (Fig. 5). In the transition state, the cleavage of the Cu–O bond, 1.91 Å (1.81 Å in **T-I4**; Table 6), is counterbalanced by the formation of the C_*ipso*_–O bond, 1.79 Å (4.39 Å in **T-I4**). The full optimization of **T-TS3** towards the products side yields intermediate **T-I5**, where the phenyl ring appears hydroxylated (*d*(C_*ipso*_–O) = 1.37 Å). Interestingly, the spin density distribution does not change to any significant extent in this reaction, with one unpaired electron located on copper and the other located on the C_6_H_5_ fragment, *e.g.*, in **T-TS3**, *ρ*(Cu) = 0.83 and *ρ*(C_6_H_5_) = 0.91 (Table 7 and Fig. 5). The oxygen rebound step is endoergic and involves the highest energy barrier of the mechanism, with **T-I5** and **T-TS3** standing 6.4 and 34.1 kcal mol^–1^ above the reactants, respectively. The rebound mechanism is thus less favourable than the EAS, in which the most energy-demanding step, the triplet-to-singlet spin crossover, involves an MECP 30.6 kcal mol^–1^ above the reactants (**MECP1**; Fig. 3).

The intermediate yielded by the rebound step, **T-I5** (Fig. 5), showed an unexpected structural feature, *i.e.* the hydroxylated phenyl ring does not appear coordinated to the metal as a neutral phenol molecule. Instead, it coordinates as a formally anionic ligand through a single Cu–C_*ortho*_ covalent bond (*d* = 2.03 Å), in which copper has a formal oxidation state of +2. This suggests that, like the EAS mechanism (Fig. 3), the reaction can only be completed by spin crossover to the singlet state. The MECP found for this process, **MECP2**, lies 9.3 kcal mol^–1^ above the reactants (Fig. 5). When the structure of **MECP2** is fully optimized in the singlet state, the system evolves towards the products side of the reaction by yielding intermediate **S-I6**. The formation of this species is exoergic by 37.9 kcal mol^–1^. In **S-I6**, phenol is formed and η^2^-coordinated to the metal through the C_*ortho*_ = C_*meta*_ moiety (*d*(Cu–C) = 2.36 and 2.49 Å). The exoergic dissociation of **S-I6** yields phenol and recovers the catalyst.

### Oxygen rebound on the substituted benzenes

The rebound pathway was recalculated for all substituted Ph–X substrates X = CF_3_, NO_2_, Cl, Me, OMe and NMe_2_ (see profiles in ESI†). The reaction pathway has the same essential features observed with benzene, *i.e.* H-abstraction yields a neutral phenyl radical, which undergoes rebound followed by triplet-to-singlet spin crossover. The bond distances (Table 8) and local spin densities (Table S2†) found for the key transition state, **T-TS3**, show the rebound of the phenyl radical to the hydroxyl through C_*ipso*_, accompanied by the binding of C_*ortho*_ to copper; *e.g.*, for X = NMe_2_, *d*(C_*ipso*_–O) = 1.79 Å and *d*(Cu–C_*ortho*_) = 2.39 Å, *ρ*(O) = 0.18 and *ρ*(C_6_H_4_X) = 0.91.

**Table 8:** Energies, in kcal mol^–1^, and selected bond distances, in Å, for **T-TS3** in the rebound pathway with Ph–X

X	Δ*G*_T-TS3_	Cu–O	C_*ipso*_–H	O–H	C_*ipso*_–O	Cu–C_*ortho*_	Cu–C_*meta*_
–NMe_2_	38.6	1.92	2.30	0.96	1.79	2.39	3.30
–OMe	34.9	1.92	2.31	0.96	1.78	2.39	3.31
–Me	36.3	1.92	2.31	0.96	1.79	2.41	3.32
–Cl	31.1	1.91	2.29	0.96	1.77	2.57	3.48
–CF_3_	29.8	1.90	2.31	0.96	1.79	2.63	3.51
–NO_2_	20.8	1.88	2.31	0.96	1.82	2.63	3.77

As for benzene, the rebound **T-TS3** barrier involves the highest energy barrier (Δ*G*_T-TS3_) with all X substituents. Interestingly, the dependence of this barrier on the donor/acceptor nature of X follows a trend opposite to that observed in the EAS mechanism; *i.e.*, the reaction is accelerated by electron-withdrawing groups and slowed down by electron-donating groups. Whereas the **T-TS2** H-abstraction barrier is almost unaffected by the nature of X (Fig. S2†), the value of Δ*G*_T-TS3_ ranges from a minimum of 20.8 kcal mol^–1^ with X = NO_2_ to a maximum of 38.6 kcal mol^–1^ with X = NMe_2_. This trend is consistent with the electron density flow observed in the critical rebound step. For X = H, the largest variations on the local charges are given by *q*(C_6_H_5_), which evolves from –0.01 (**T-I4**) to –0.20 (**T-TS3**) and –0.37 (**T-I5**), and by *q*(O), which evolves from –1.10 (**T-I4**) to –0.94 (**T-TS3**) and –0.74 (**T-I5**). These charges show an electrophilic attack of the phenyl ring to the hydroxyl. Furthermore, the most electron-withdrawing groups of the series, X = CF_3_ and NO_2_, yield barriers that are lower than those observed for the EAS mechanism. With X = NO_2_, the observation of radical species should be hampered by fast rebound, which, in this case, involves a low barrier of 20.8 kcal mol^–1^. We note that the yields obtained with X = CF_3_ and NO_2_ (Fig. 1) are somewhat lower than those expected from the calculated rebound barriers which do not agree with the Hammett equations derived experimentally (Fig. 1). This suggests that the reaction either follows a different mechanism or generates radical species from intermediate **T-I4** (Fig. 5) giving rise to undesired homo-coupling by-products and detrimental side reactions hampering the access to **T-TS3**. Furthermore, the accuracy of the DFT calculations may not be high enough in these two particular cases.

## Conclusions

Two competitive pathways have been found for the benzene (and substituted benzenes) hydroxylation reaction, with an unprecedented dual behavior of a copper-oxyl intermediate. Using hydrogen peroxide as the oxidant, in the absence of acids, and using Tp^*x*^Cu (Tp^*x*^ = hydrotrispyrazolylborate) as the catalyst, experimental evidences have allowed first discarding Fenton-like mechanisms and later proposing the plausible competition between an electrophilic aromatic substitution pathway with the alternative rebound (hydrogen abstraction) route. Both pathways involve a common copper-oxyl species of type Tp^*x*^Cu–O˙. Based on the theoretical calculations, the EAS mechanism proposed in this work is not the conventional one, since it requires spin crossover after the addition step. Nonetheless, it follows the expected trend of being accelerated by electron-donating groups and decelerated by electron-withdrawing groups, which is in line with the Hammett plots derived experimentally. In contrast, the rebound mechanism follows the opposite trend, although it accounts for the formation of halobenzenes derived from the C_6_H_5_˙ radical intermediate. In the rebound mechanism, this radical is generated in the initial H abstraction step, which is reversible and faster than the addition step in the EAS mechanism. The observation of two competitive pathways and the dual behavior of the Cu-oxyl species may serve for future design of novel catalysts toward the direct functionalization of benzene into phenol.

## Conflicts of interest

There are no conflicts to declare.

## Supplementary Material

Supplementary information
