# Supplementary material for: A competing, dual mechanism for catalytic direct benzene hydroxylation from combined experimental-DFT studies
Source: Chem Sci. 2017 Oct 5;8(12):8373–83. doi: 10.1039/c7sc02898a (PMC5863614; doi:10.1039/c7sc02898a)
Supplement: Supplementary file 1 [file SC-008-C7SC02898A-s001.pdf]

Electronic Supplementary Material (ESI) for Chemical Science.
This journal is © The Royal Society of Chemistry 2017

Electronic Supporting Information for

A competing, dual mechanism for the catalytic direct benzene hydroxylation from combined experimental -DFT studies

Laia Vilella,[a] Ana Conde,[b] David Balcells,*[,c] M. Mar Díaz-Requejo,*[,b] Agustí Lledós,*[,a] and Pedro J. Pérez.*[,b]

[a]Departament de Química, Universitat Autònoma de Barcelona, 08193 Bellaterra, Spain. [b]Laboratorio de Catálisis Homogénea, Departamento de Química y Ciencia de los Materiales, Unidad Asociada al CSIC, Centro de Investigación en Química Sostenible (CIQSO), Universidad de Huelva, Campus de El Carmen 21007 Huelva, Spain. [c]Centre for Theoretical and Computational Chemistry, Department of Chemistry, University of Oslo. P.O. Box 1033 Blindern, N-0315 Oslo, Norway.

Content:

1. General information. S2
2. Catalytic Procedure of Toluene Oxidation. S2
3. Catalytic Procedure Oxidation of Benzene in the Presence of CCl_4 or $CBrCl_3$. S3
4. Catalytic Procedure Using Oxone as Oxidant. S4
5. Catalytic Procedure Oxidation of Substituted Benzenes. S5
6. Catalytic Procedure to Determine the Kinetic Isotopic Effect. S10
7. Computational Details S11
8. Reactivity of hydroperoxo and superoxo species. S11
9. Energy profiles for the EAS on the substituted benzenes. S13
10. 1,3-cyclohexadienone and benzene oxide tautomers. S16
11. Selected local charges and spin densities for T-I2 in the EAS pathway. S16
12. Energy profiles for the rebound mechanism on the substituted benzenes. S17
13. Selected spin densities for T-TS3 in the rebound pathway. S20
14. References S20
15. XYZ coordinates of all the optimized structures. S21

1. General information.

All air– and moisture–sensitive manipulations were carried out with standard Schlenck techniques under nitrogen atmosphere. All solvents and reagents were purchased from commercial sources in anhydrous packages and used without any further purification. The Tp^x ligands[1] and the complexes $[Tp^xCu(NCMe)]$[2] were prepared according to the literature procedures. All products were purchased and employed as reference in the analyses of the reaction mixtures. GC analyses were performed on Varian 3800 and 3900 chromatographs equipped with 60 m x 0.25 mm x 1.00 μm (MS detector) and ZB – 1MS 30 m x 0.25 mm x 0.25 μm (FID detector) columns.

2. Catalytic Procedure of Toluene Oxidation.

The reactions were performed in a 25 mL round-bottomed flask equipped with a reflux condenser and a magnetic stirrer bar. In a typical experiment, 0.01 mmol of catalyst was dissolved in 1 mL of acetonitrile and 1 mmol (106 μL) of toluene and 10 mmol (1 mL) of an aqueous commercial solution of hydrogen peroxide (30% v/v) were added. The mixture was stirred for 8 h at 75 °C. Additional dichloromethane (2 × 2.5 mL) was then added to extract the organic products. Styrene was added as internal standard and a sample of the mixture was directly analyzed by GC/GCMS.

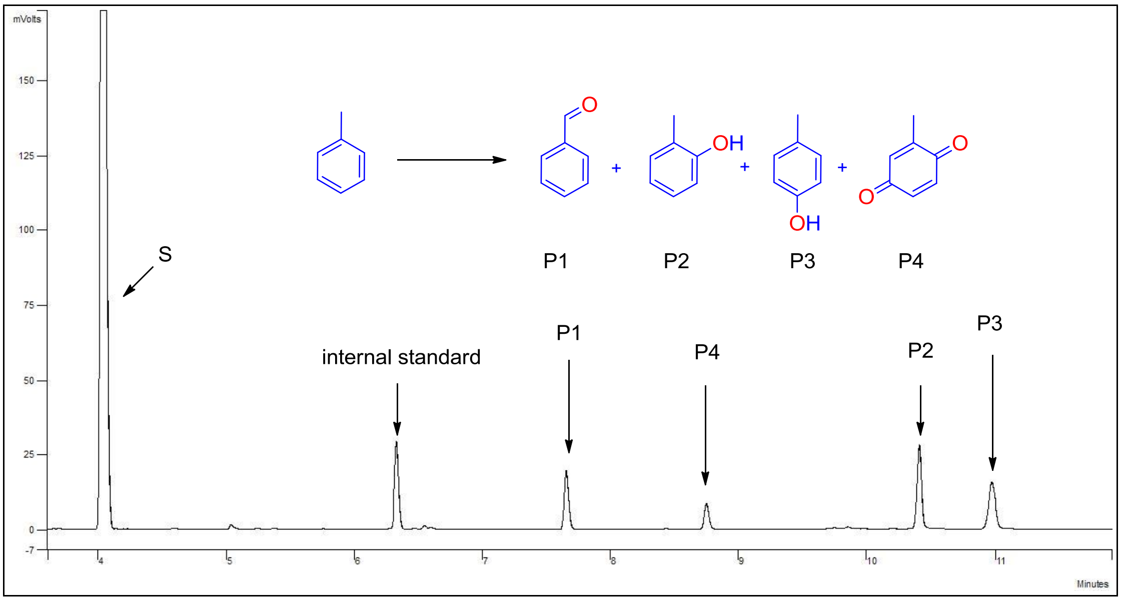

Figure S1. GC trace of the final reaction mixture of toluene oxidation.

3. Catalytic Procedure Oxidation of Benzene in the Presence of CCl_4 or $CBrCl_3$.

The reaction was performed in a 25 mL round-bottomed flask equipped with a reflux condenser and a magnetic stirrer bar. $Tp^{*,Br}Cu$ (0.01 mmol) was dissolved in 2.5 mL of acetonitrile and 0.5 mL of CCl_4 and 1 mmol of benzene and 5 mmol (ca. 0.5 mL) of an aqueous commercial solution of hydrogen peroxide (30% v/v) were added. The mixture was stirred for 4 h at 80 °C. After cooling at room temperature, additional dichloromethane (2.5 mL) was added to extract the organic products. Analysis of the final reaction mixture was done by GC with cycloheptanone as internal standard. The reaction with $CBrCl_3$ was performed in a similar manner using 2.5 mL of acetonitrile and 0.5 mL of $CBrCl_3$.

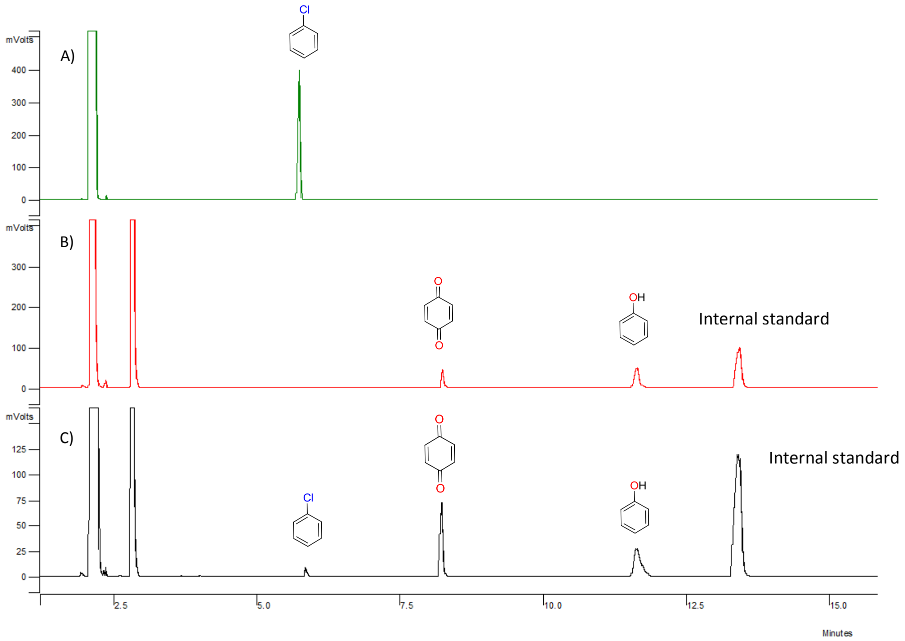

Figure S2. A) GC trace of commercial Ph-Cl. B) GC trace of the benzene oxidation reaction. C) GC trace of the benzene oxidation reaction in the presence of CCl_4 as additive.

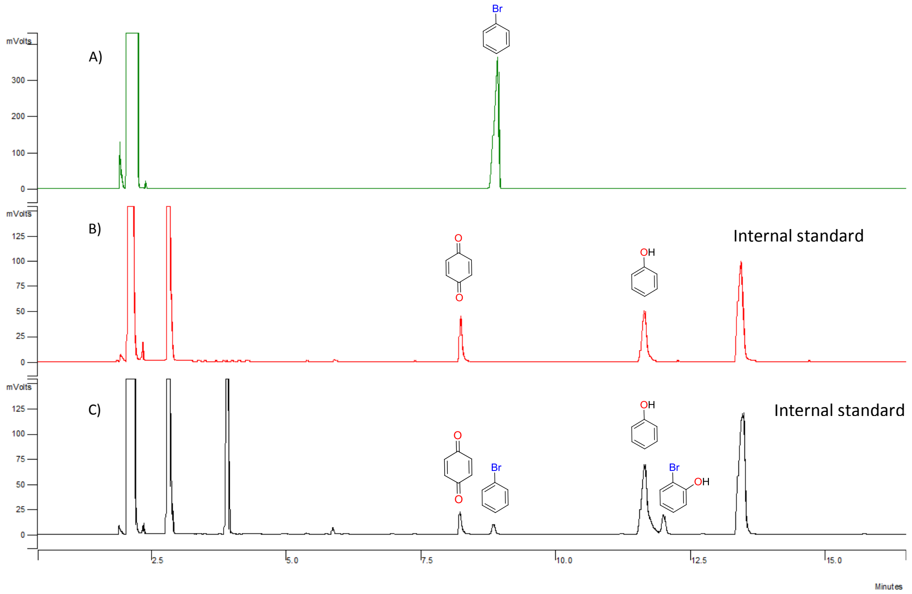

Figure S3. A) GC trace of commercial Ph-Br. B) GC trace of the benzene oxidation reaction. C) GC trace of benzene oxidation reaction in the presence of CCl_3Br as additive.

4. Catalytic Procedure Using Oxone as Oxidant.

The reactions were performed in an ampule. In a typical experiment, 0.01 mmol of catalyst $Tp^{*,Br}Cu(NCMe)$ was dissolved in 3 mL of acetonitrile and 5 mmol of benzene. A solution of 1 mmol of oxone and 1 mmol of $NaHCO_3$ in 3 mL of water was added in one portion. The mixture was stirred for 5h at 80 °C. After cooling at room temperature, the aqueous phase was extracted with $CDCl_3$ (2.5 mL) followed by treatments of the organic phase with $MgSO_4$. An exactly weighted amount of diethyl malonate was added as internal standard and the mass balance in the organic phase was then determined by ^1H NMR.

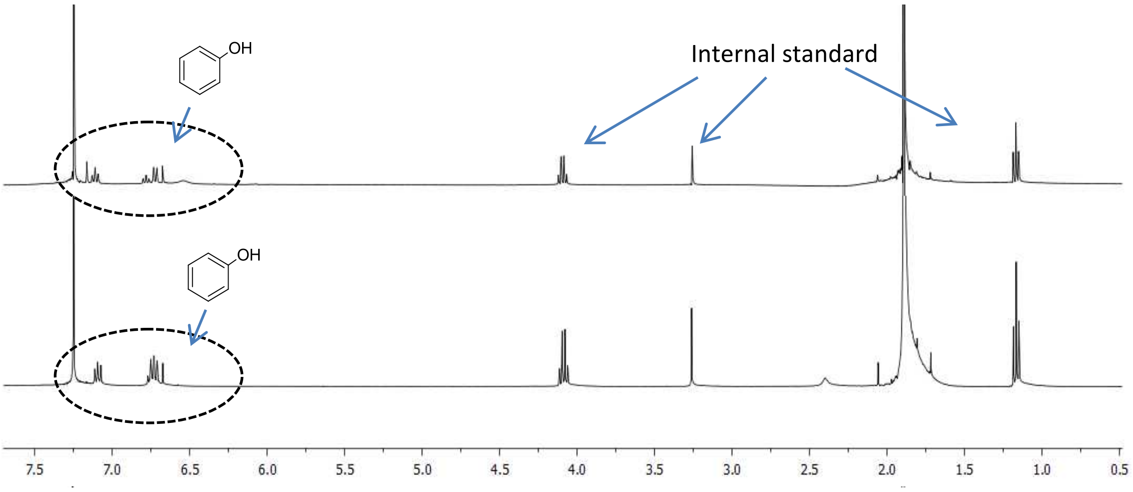

Figure S4. NMR spectra of the reaction mixtures of benzene oxidation with Oxone (above) or H_2O_2 (below) as oxidant.

5. Catalytic Procedure Oxidation of Substituted Benzenes.

The reactions were performed in round-bottom flasks of 25 ml which has equipped a reflux condenser and a magnetic bar. In a typical experiment, 0.01 mmol of catalyst $Tp^xCu(NCMe)$ ($Tp^x = Tp^{*,Br}$, Tp^{Br3}) was dissolved in 3 mL of MeCN and 1 mmol of substrate was added. H_2O_2 (5mmol) was added and the solution was stirred for 4h at 80 ° C. After cooling at room temperature, additional dichloromethane (2.5 mL) was added to extract the organic products. Analysis of the final reaction mixture was done by GC using cycloheptanone as internal standard to determine the conversion and the ratio of products formed.

Chromatographic methods.

Chromatographic separation of the various products shown in this work was performed using the following chromatographic methods:

◆ For benzene oxidation reactions:

- Column: Zebron ZB-5Msi (30m L; 0.25mm ID; 0.25µm df)
- Detector: FID
- Initial column temperature: 60 ºC
- Final column temperature: 250 ºC
- Temperature ramp: 1 ºC/min
- Injector temperature: 225 ºC
- Detector temperature: 255 ºC

◆ For substrates as Ph-NMe_2, Ph-OMe, Ph-Cl, Ph-CO_2Me y Ph-CF_3:

- Column: Zebron ZB-5Msi (30m L; 0.25mm ID; 0.25µm df)
- Detector: FID
- Initial column temperature: 80 ºC
- Final column temperature: 250 ºC
- Temperature ramp: 2 ºC/min
- Injector temperature: 225 ºC
- Detector temperature: 255 ºC

◆ For substrates as Ph-Me and Ph-NO_2:

- Column: Zebron ZB-5Msi (30m L; 0.25mm ID; 0.25µm df)
- Detector: FID
- Initial column temperature: 60 ºC
- Final column temperature: 250 ºC
- Temperature ramp: 12 ºC/min
- Injector temperature: 225 ºC
- Detector temperature: 255 ºC

Analysis of the final reaction mixture was carried out using cycloheptanone as internal standard in all cases, except for toluene for which 1,2-dichlorobenzene was used.

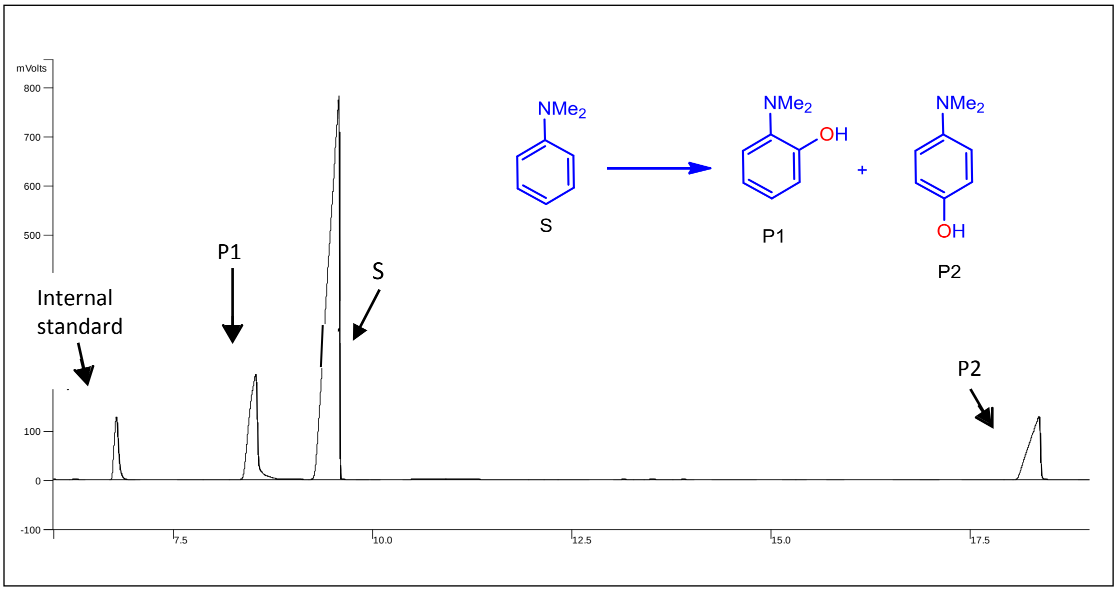

Figure S5. GC trace of the reaction mixture of the N,N-dimethylaniline oxidation reaction

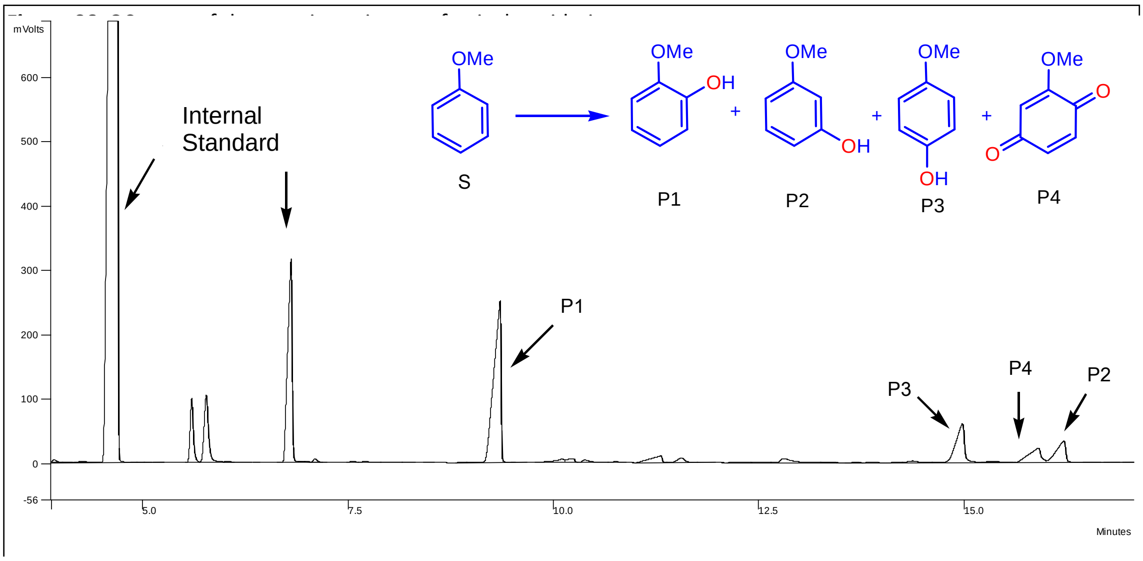

Figure S6. GC trace of the reaction mixture of anisole oxidation. The peaks at 5.5 min have not been identified.

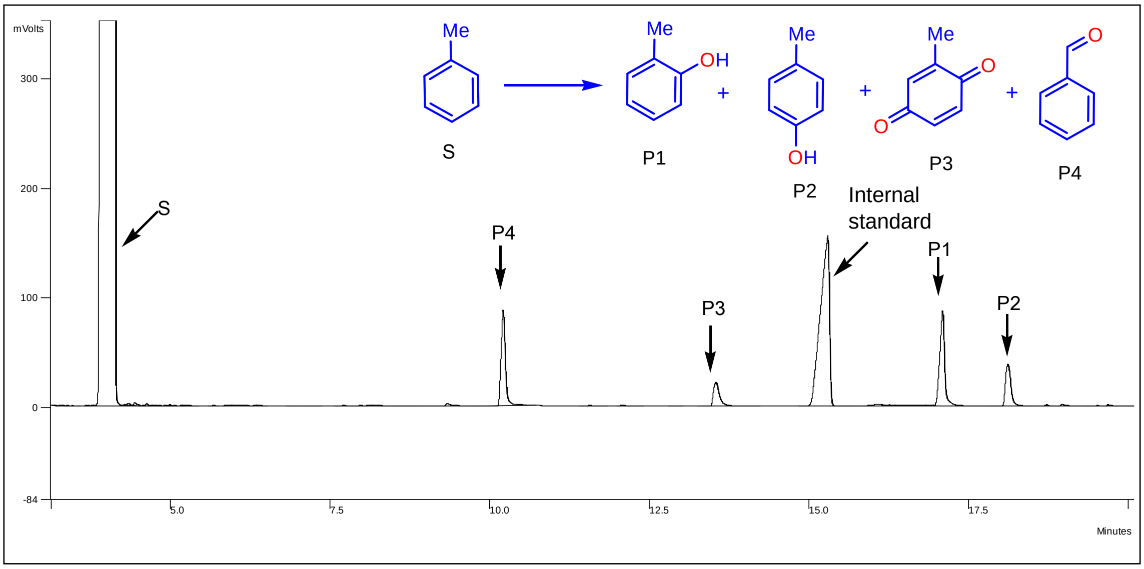

Figure S7. GC trace of the final reaction mixture of toluene oxidation.

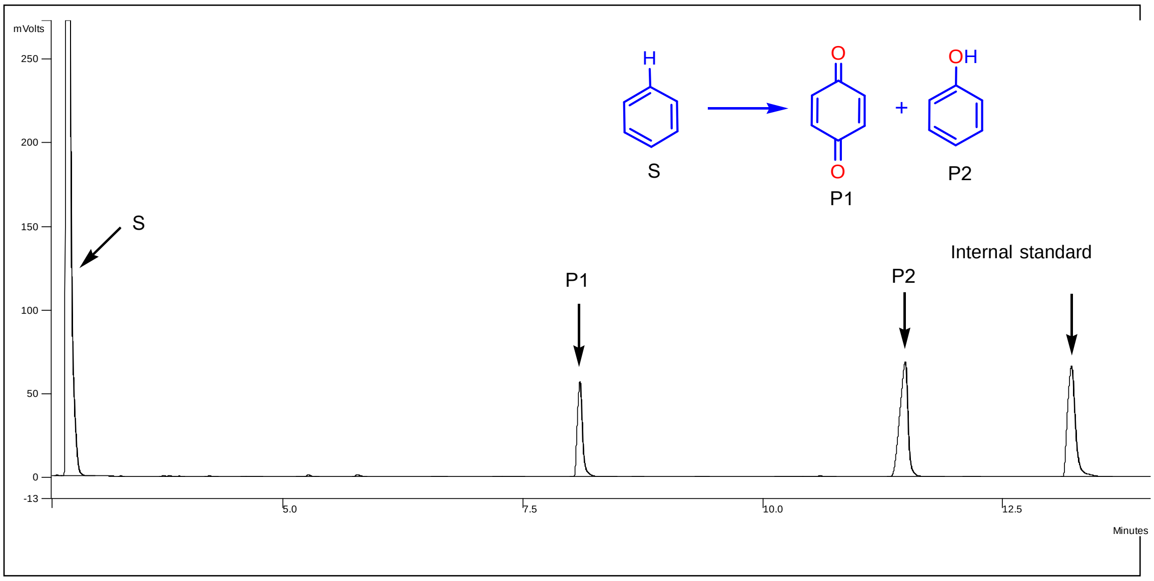

Figure S8. GC trace of the final reaction mixture of benzene oxidation.

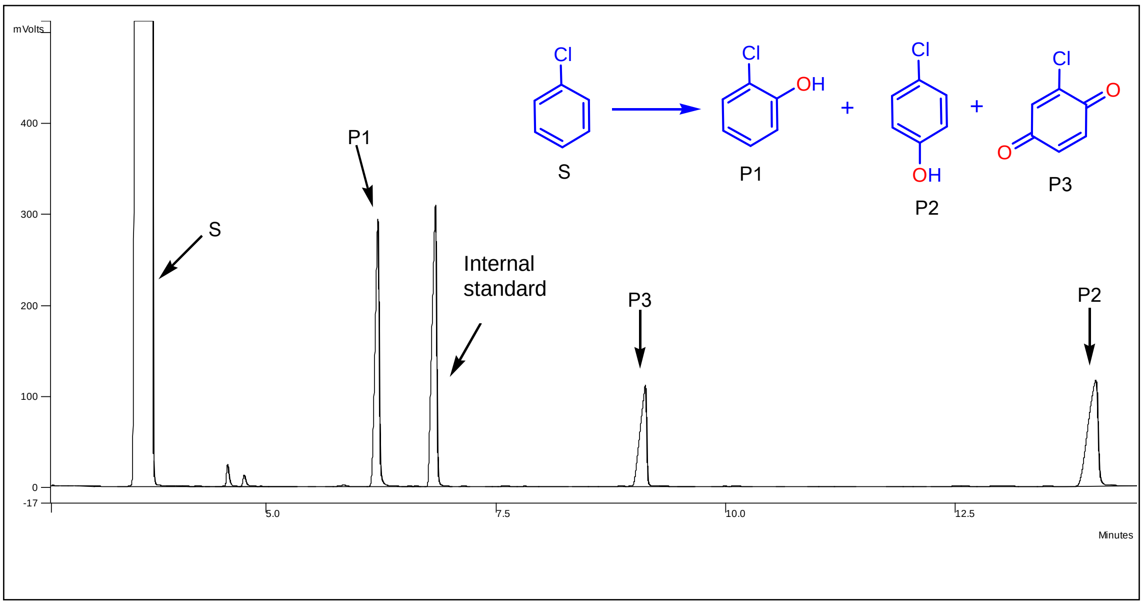

Figure S9. GC trace of the final reaction mixture of chlorobenzene oxidation.

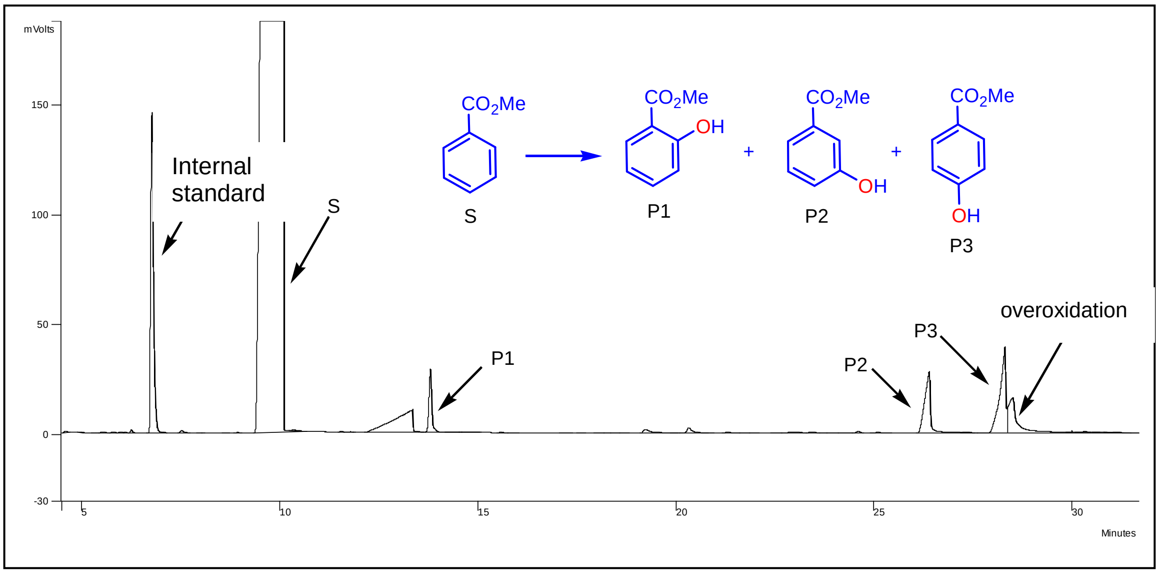

Figure S10. GC trace of the final reaction mixture of methyl-benzoate oxidation.

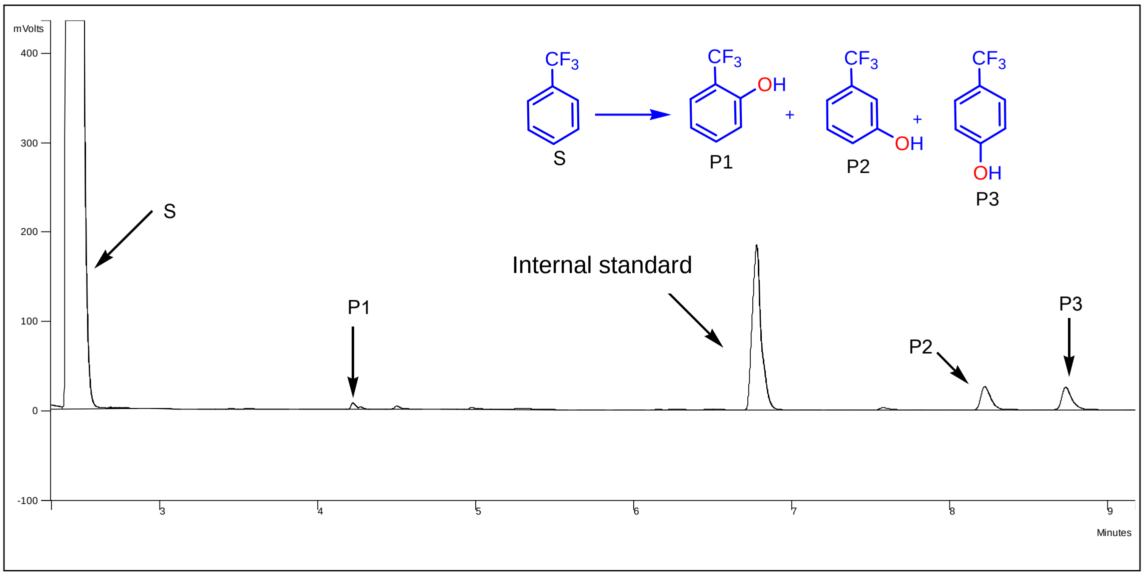

Figure S11. GC trace of the final reaction mixture of (trifluoromethyl)benzene oxidation.

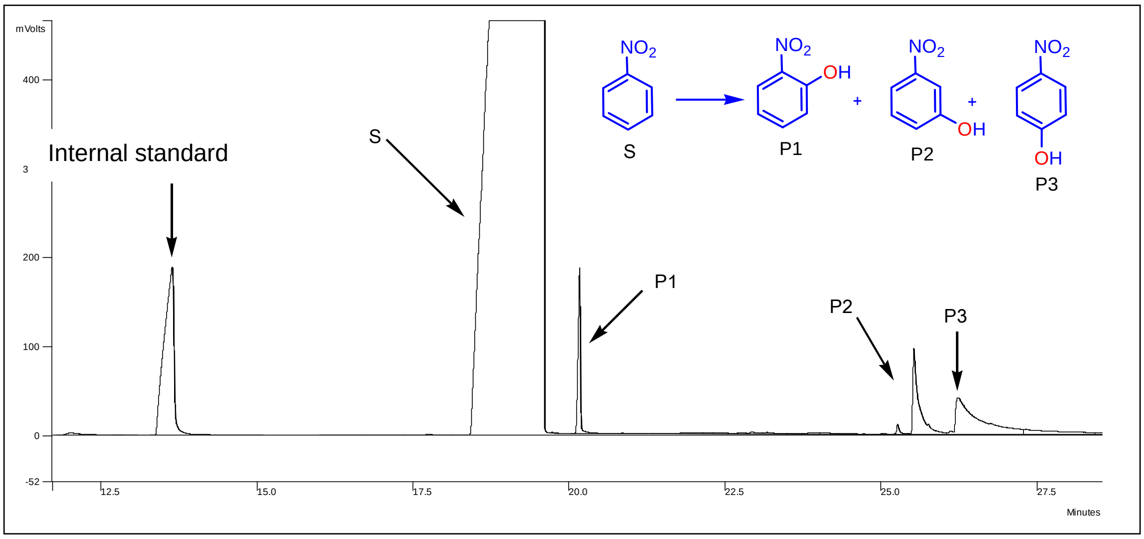

Figure S12. GC trace of the final reaction mixture of nitrobenzene oxidation.

6. Catalytic Procedure to Determine the Kinetic Isotopic Effect.

The reaction was performed in a 25 mL round-bottomed flask equipped with a reflux condenser and a magnetic stirrer bar. $Tp^{*,Br}Cu(NCMe)$ (0.005 mmol) was dissolved in 2 mL of acetonitrile and an equimolar mixture of benzene with deuterated benzene (1:1 mmol) and 1.5 mmol of an aqueous commercial solution of hydrogen peroxide (30% v/v) were added. The mixture was stirred for 4 h at 80 °C. After cooling at room temperature, additional dichloromethane (2.5 mL) was added to extract the organic products. Analysis of the final reaction mixture was performed by GC-MS.

Reaction mixture

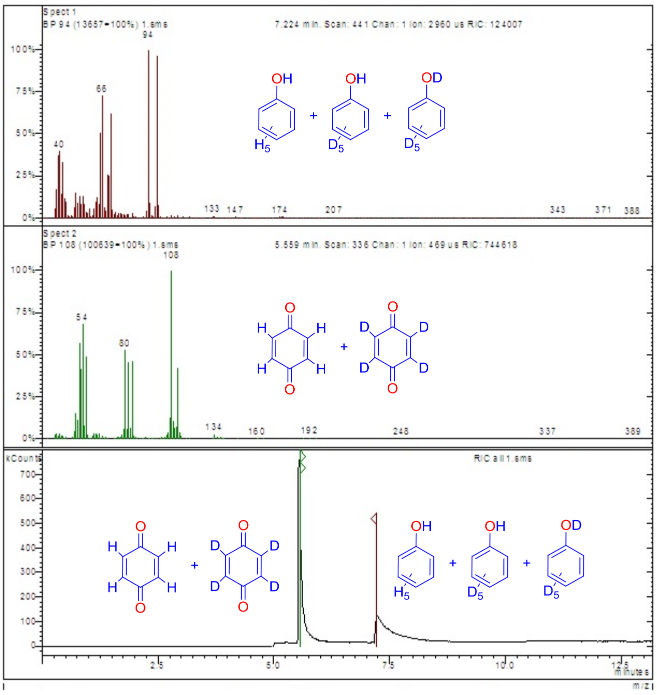

RIC and separation of ions by selecting molecular ion

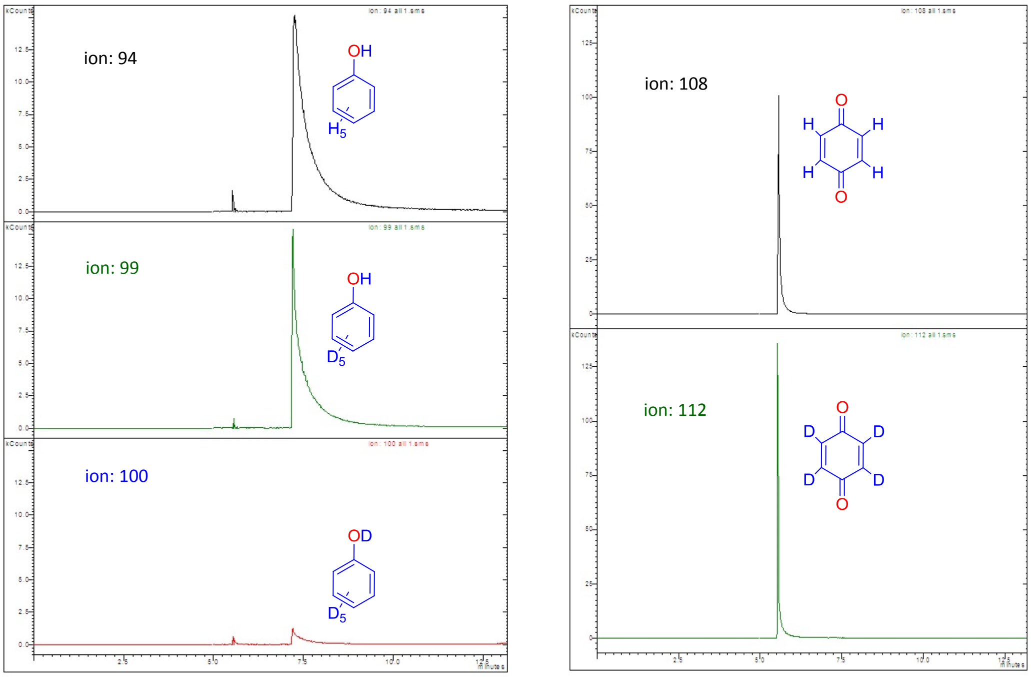

Figure S13. GC and GCMS traces for the competition experiments between C_6H_6 and C_6D_6.

7. Computational Details.

All the calculations presented in this work were carried out at the Density Functional Theory (DFT) level using the BHandHLYP exchange-correlation functional[3], by means of the Gaussian09 software. This functional was selected based on previous studies from Sodupe and Rodríguez-Santiago[4] on a series of copper-aqua complexes. Therein, the accuracy of several DFT functionals was assessed, giving the BHandHLYP functional the most accurate results compared to benchmark CCSD(T) calculations.

The H, B, C, N and O atoms were described with a double-ζ 6-31G(d,p) basis set, whereas for the Br and Cu atoms the Stuttgart-Bonn scalar relativistic ECP[5] was used, with its associated basis set. The structures of all the stationary points involved in the reaction were fully optimized in gas phase without any geometry or symmetry constraints. From these geometries, vibrational frequencies were computed analytically in order to characterize them as either energy minima (reactants, intermediates and products) or saddle points (transition states). The located transition states were further confirmed to connect the corresponding reactants and products by means of Intrinsic Reaction Coordinate (IRC) calculations. Frequency calculations were also used to determine the difference between the potential and Gibbs energies, $(G - E)_g$, which includes the zero-point energy and the thermal and entropy corrections. Solvent effects (acetonitrile, ε = 35.688), E_{sol}, were introduced through single point calculations on the gas phase optimized structures using the SMD[6] solvation model with the larger triple-ζ 6-311+G** basis set for the H, B, C, N and O atoms. All the energies reported throughout this work correspond to Gibbs energies in acetonitrile, G_{solv}, calculated as follows:

$$G_{sol} = (G - E)_g + E_{sol}$$

In order to change the standard state from the gas phase (1 atm) to solution (1M) a correction of 1.9 kcal mol^{-1} was applied to all G values. This implies that ΔG_{sol} values were corrected by 1.90 kcal mol^{-1} when one species is generated from two, i.e. one unit of transition metal catalyst and a molecule of benzene.[7] Therefore, all the energies reported throughout this work correspond to Gibbs energies in solution (G_{sol}) at the temperature of 298.15 K and 1 M.

Local charges and spin densities were obtained from Natural Population Analysis (NPA) calculations.[8] The MECPs were located with the program developed by Harvey et al.[9] In order to infer how the MECPs relate to the crossing potential energy surfaces, their structures were optimized for the two spin states involved. The Gibbs energies in solution of the MECPs were obtained by using the energy corrections found for the stationary points on the reactants side.

8. Reactivity of hydroperoxo and superoxo species.

The superoxo species, which is a triplet in its ground state, can be added to one carbon atom of benzene (EAS) or abstract one hydrogen atom, giving rise to the phenyl radical (rebound) (Figure S14). In contrast, the hydroperoxo species, which is a doubled in its ground state, evolves through a copper-oxygen addition into a C=C bond from benzene (Figure S15).

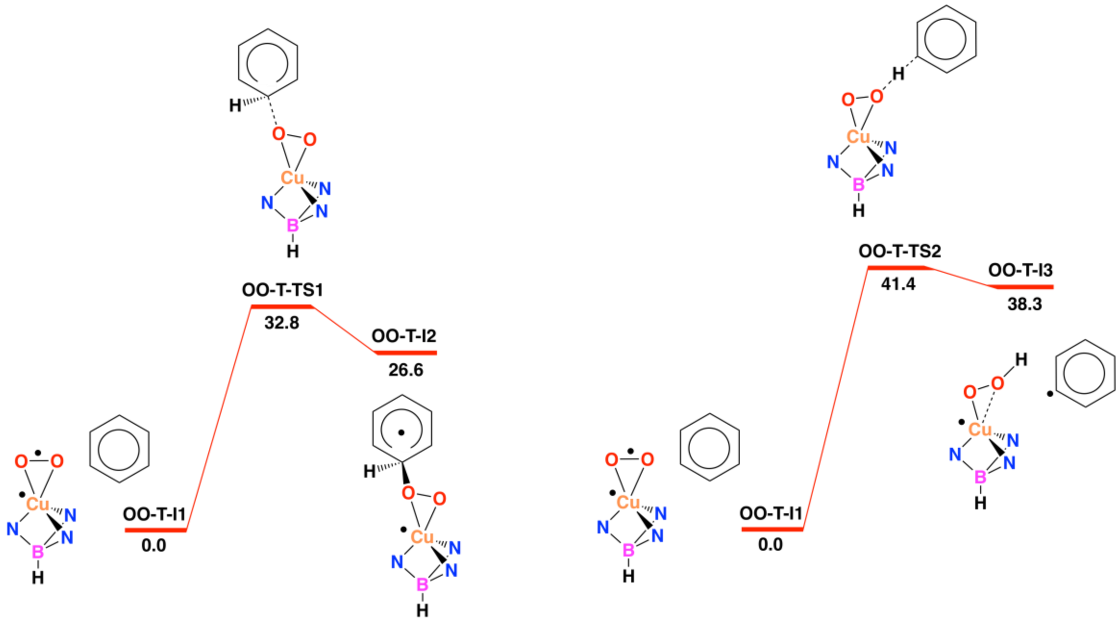

Figure S14 . Gibbs energy profile in solution (G_{sol}) profiles, in kcal.mol-1, for the oxidation of benzene by superoxo species by means of EAS (left) and rebound (right) mechanisms. The triplet spin state of the stationary points is depicted in red.

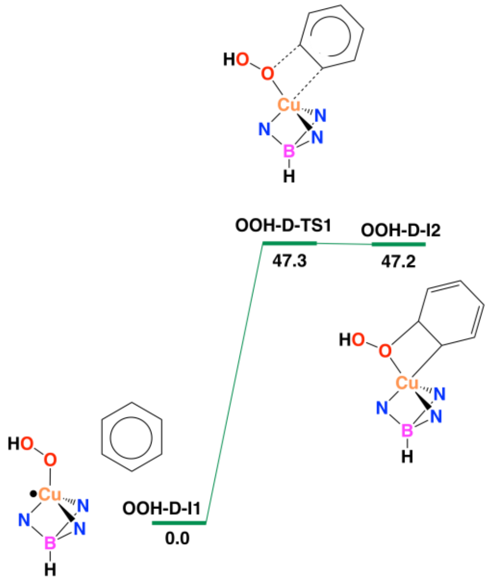

Figure S15. Gibbs energy profile in solution (Gsol) profile, in kcal.mol-1, for the oxidation of benzene by hydroperoxo species via copper-oxygen addition into the C=C bond. The doublet spin state of the stationary points is depicted in green.

9. Energy profiles for the EAS on the *para*-substituted benzenes.

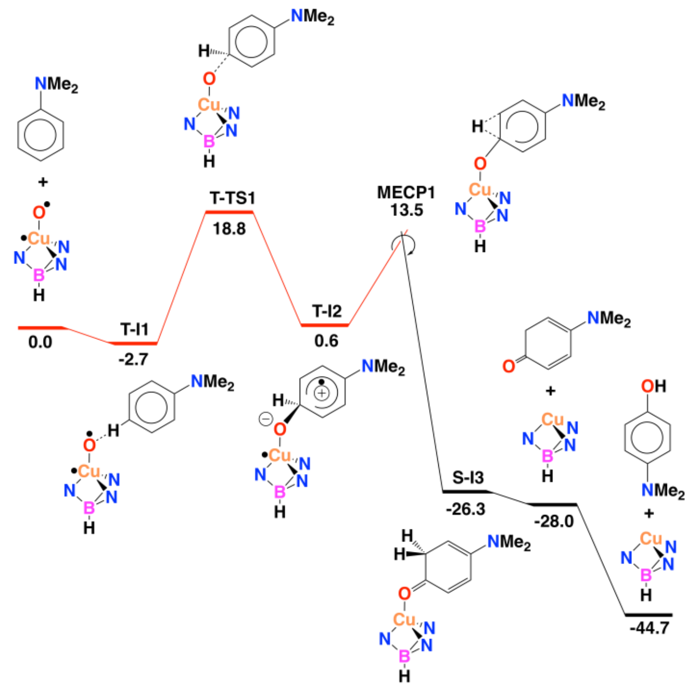

Figure S16. Gibbs energy profile in solution, in kcal mol^{-1}, of the electrophilic aromatic substitution (EAS) mechanism with the *para*-substituted benzene Ph-NMe_2.

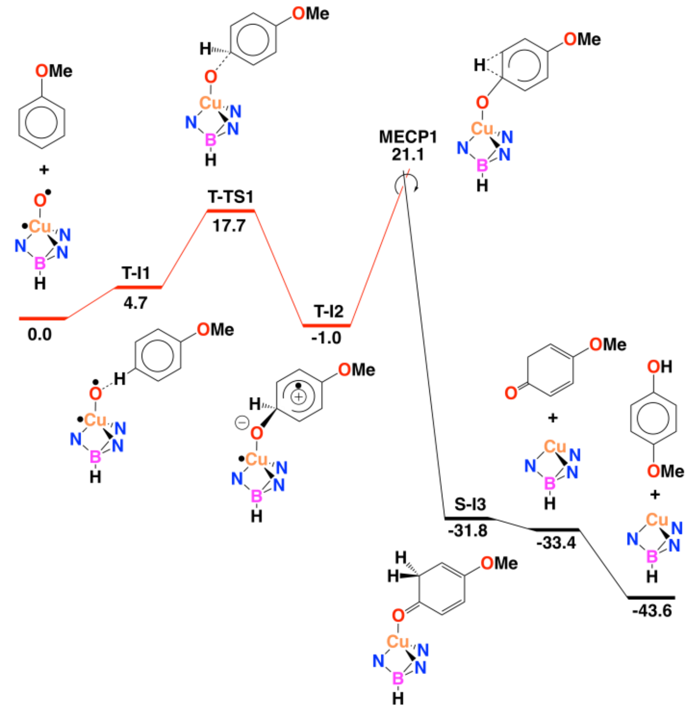

Figure S17. Gibbs energy profile in solution, in kcal mol^{-1}, of the electrophilic aromatic substitution (EAS) mechanism with the *para*-substituted benzene Ph-OMe.

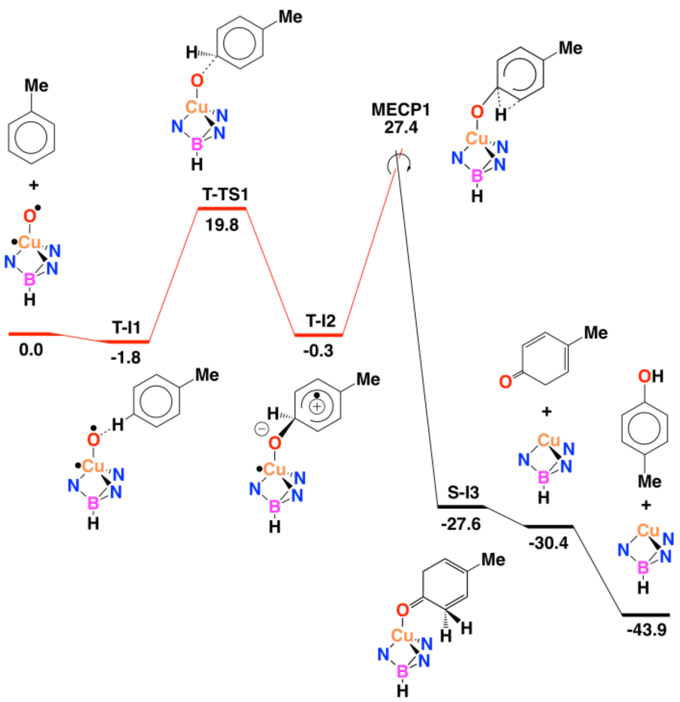

Figure S18. Gibbs energy profile in solution, in kcal mol^{-1}, of the electrophilic aromatic substitution (EAS) mechanism with the *para*-substituted benzene Ph-Me.

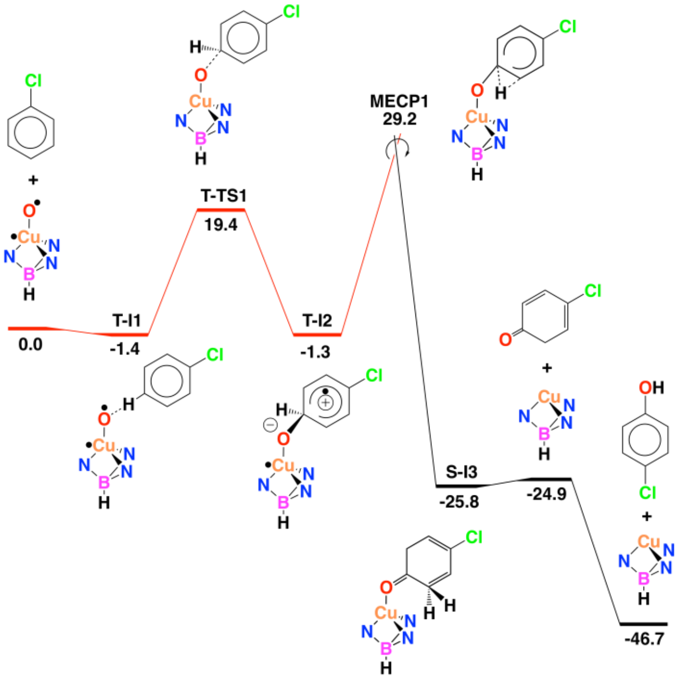

Figure S19. Gibbs energy profile in solution, in kcal mol^{-1}, of the electrophilic aromatic substitution (EAS) mechanism with the *para*-substituted benzene Ph-Cl.

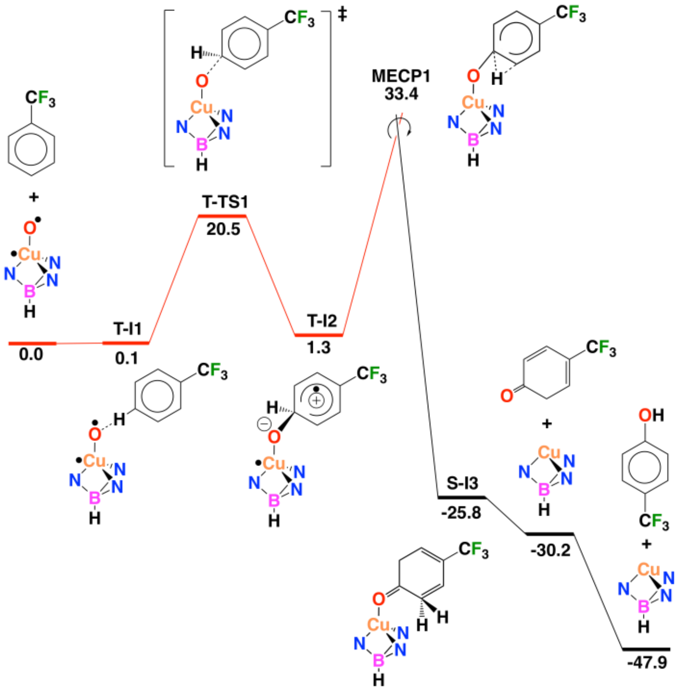

Figure S20. Gibbs energy profile in solution, in kcal mol^{-1}, of the electrophilic aromatic substitution (EAS) mechanism with the *para*-substituted benzene Ph-CF_3.

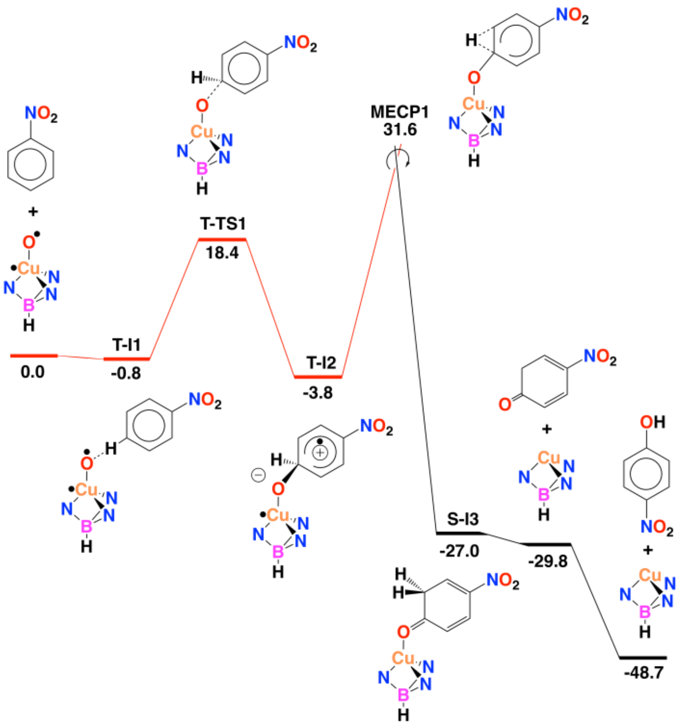

Figure S21. Gibbs energy profile in solution, in kcal mol^{-1}, of the electrophilic aromatic substitution (EAS) mechanism with the *para*-substituted benzene Ph-NO_2.

10. 1,3-cyclohexadienone and benzene oxide tautomers.

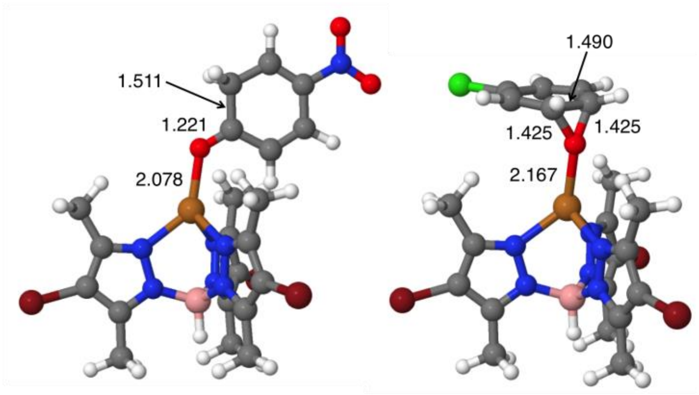

Figure S22. Optimized geometries of O-S-I4 for $-NO_2$ (left) and -Cl (right), which gave rise to 1,3-cyclohecadienone and benzene oxide respectively in the SEAr pathway. Color code: orange = Cu, blue = N, red = O, pink = B, maroon = Br, grey = C, white = H, green = Cl.

11. Selected local charges and spin densities for T-I2 in the EAS pathway.

Table S1. Energies, in kcal mol^{-1}, and selected local charges (q) and spin densities (ρ), in a.u., for **T-I2** in the EAS pathway with Ph-X.[a]

X	$G_{T\text{-}I2}$	| q |[b]	ρ(Cu)	ρ(O)	ρ(C_6H_6)	ρ(Tp*,Br)
$-NMe_2$	0.6	0.30	0.81	0.15	0.95	0.09
-OMe	-1.0	0.29	0.81	0.15	0.95	0.09
-Me	-0.3	0.28	0.82	0.14	0.95	0.09
-Cl	-1.3	0.27	0.82	0.14	0.95	0.09
$-CF_3$	1.3	0.26	0.84	0.15	0.93	0.08
$-NO_2$	-3.8	0.26	0.82	0.12	0.97	0.09

[a]q and ρ are are not given for **MECP1** due to different values in the singlet and triplet states. [b]Absolute values of the local charges of the C_6H_5X (> 0) and CuOTp*,Br (< 0) fragments; q(C_6H_5X) = -q(CuOTp*,Br).

12. Energy profiles for the rebound mechanism on the substituted benzenes.

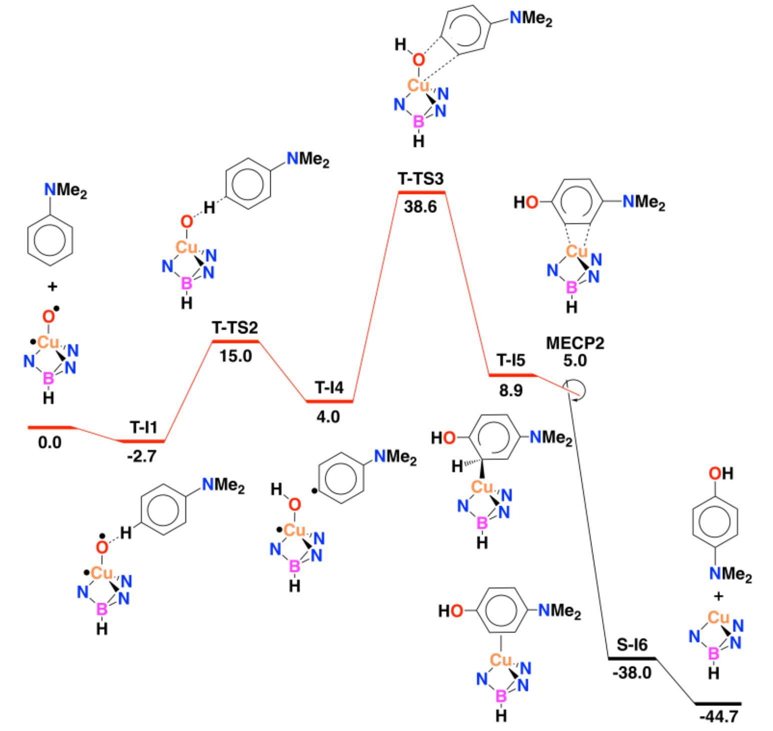

Figure S23. Gibbs energy profile in solution, in kcal mol^{-1}, of the rebound mechanism with the *para*-substituted benzene Ph-NMe_2.

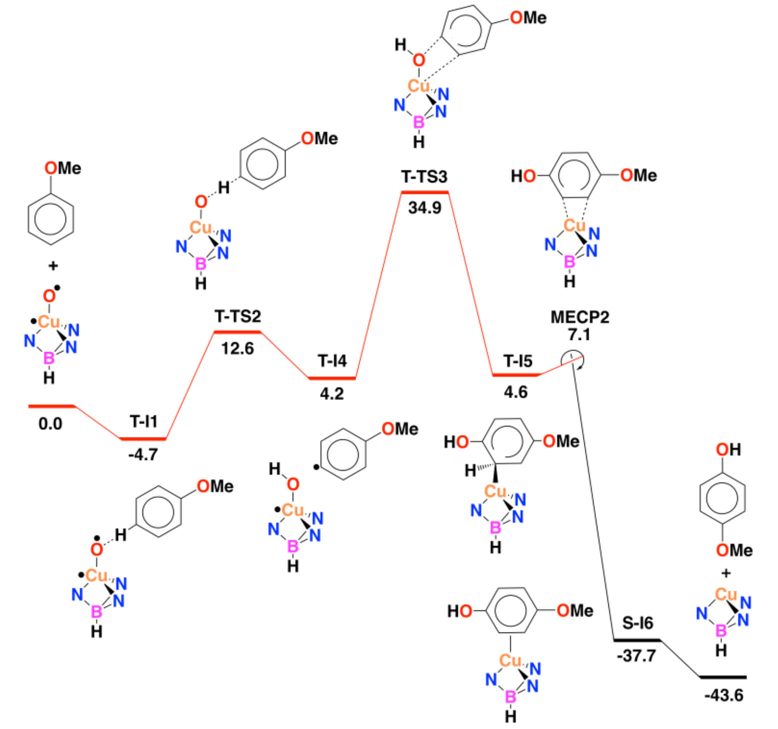

Figure S24. Gibbs energy profile in solution, in kcal mol^{-1}, of the rebound mechanism with the *para*-substituted benzene Ph-OMe.

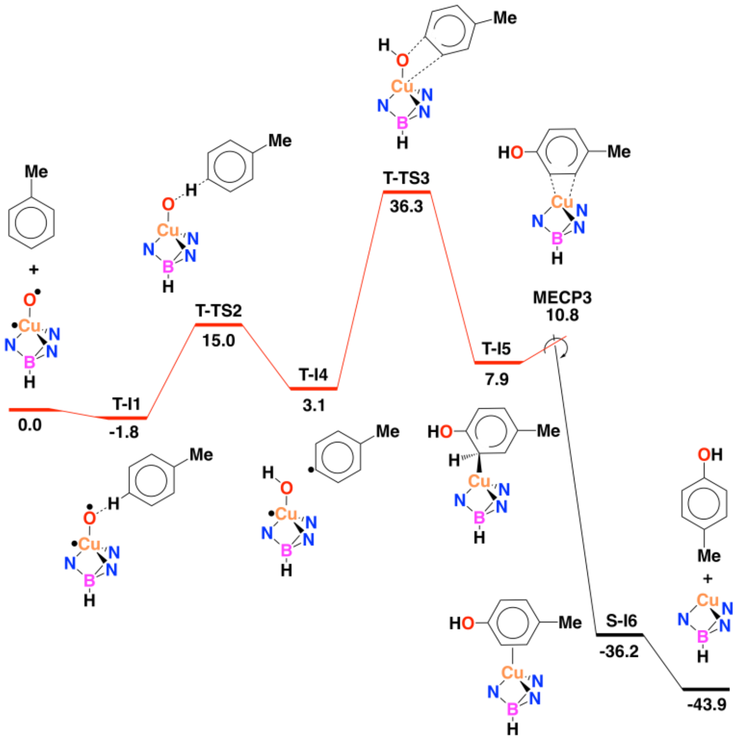

Figure S25. Gibbs energy profile in solution, in kcal mol^{-1}, of the rebound mechanism with the *para*-substituted benzene Ph-Me.

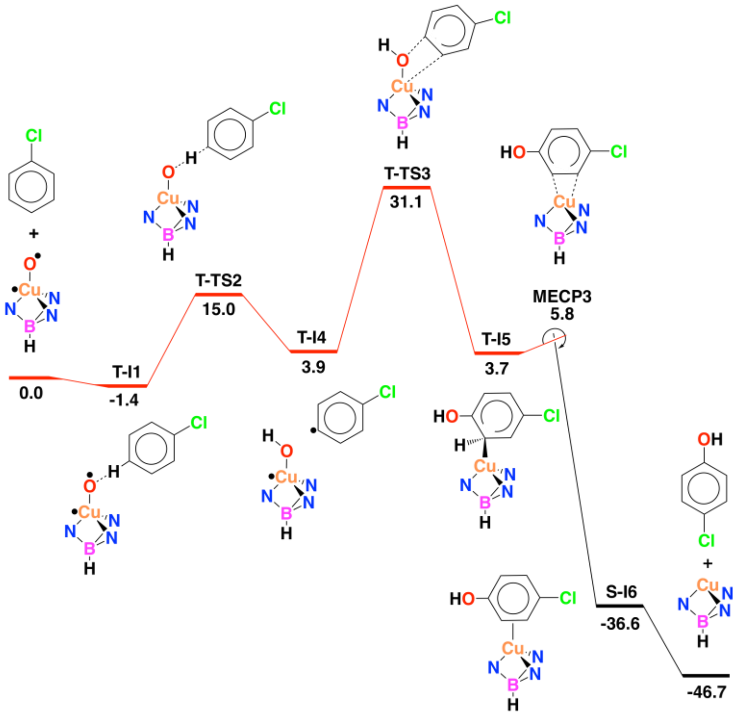

Figure S26. Gibbs energy profile in solution, in kcal mol^{-1}, of the rebound mechanism with the *para*-substituted benzene Ph-Cl.

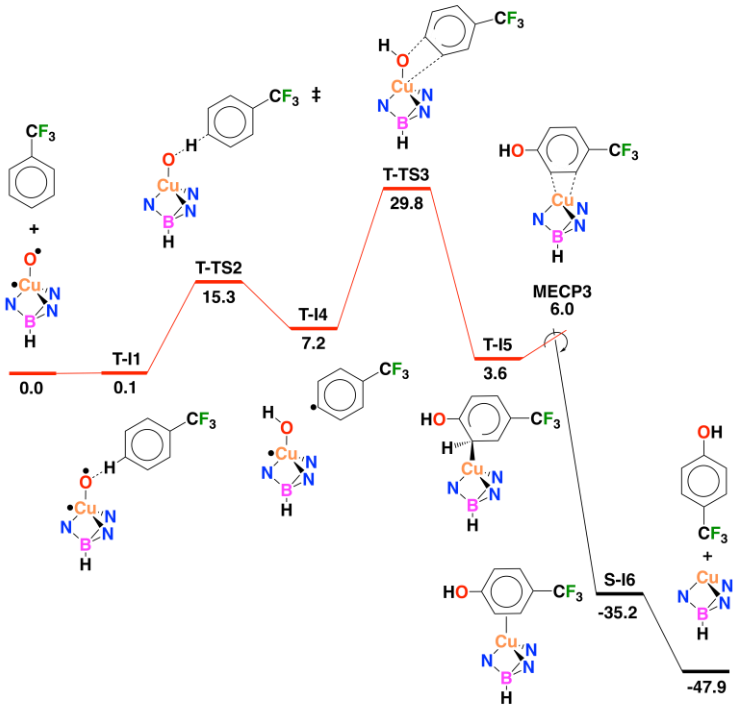

Figure S27. Gibbs energy profile in solution, in kcal mol^{-1}, of the rebound mechanism with the *para*-substituted benzene Ph-CF_3.

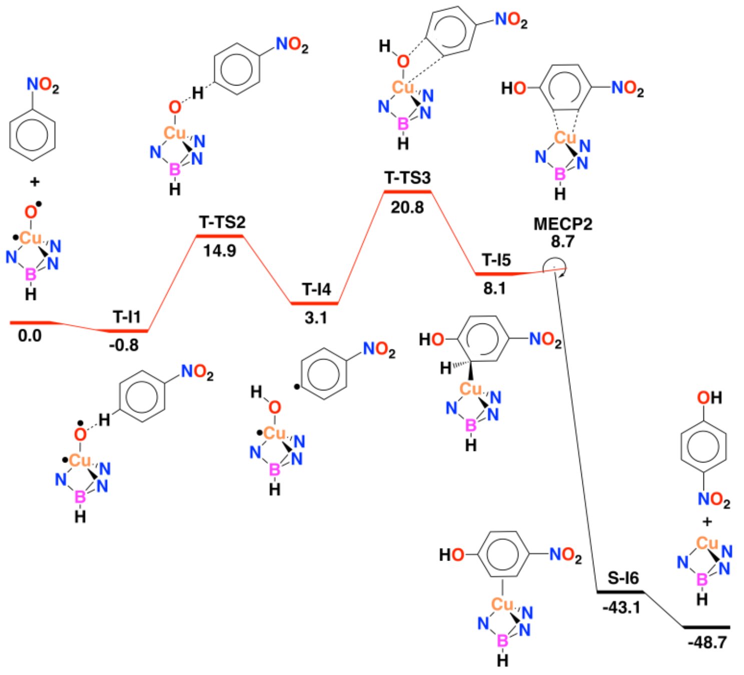

Figure S28. Gibbs energy profile in solution, in kcal mol^{-1}, of the rebound mechanism with the *para*-substituted benzene Ph-NO_2.

Table S2. **T-TS2** Gibbs energies in solution in kcal mol^{-1}.

-X	$G_{T\text{-}TS2}$
$-NMe_2$	15.0
-OMe	12.6
-Me[d]	15.0
-Cl	15.0
$-CF_3$	15.3
$-NO_2$	14.9

13. Selected spin densities for T-TS3 in the rebound pathway.

Table S3. Selected spin densities (ρ), in a.u., for in the rebound pathway with Ph-X.

ρ	ρ(Cu)	ρ(O)	ρ(H)	$\rho(C_6H_4X)$	ρ(Tp*,Br)
$-NMe_2$	0.82	0.18	0.01	0.91	0.08
-OMe	0.82	0.18	0.01	0.91	0.08
-Me[d]	0.82	0.18	0.01	0.91	0.08
-Cl	0.84	0.17	0.01	0.89	0.09
$-CF_3$	0.85	0.16	0.00	0.90	0.09
$-NO_2$	0.86	0.17	-0.01	0.87	0.11

14. References.

1- (*a*) S. Trofimenko in *Scorpionates*, *The Coordination Chemistry of Polypyrazolylborate Ligands;* Imperial College Press: London, 1999; (*b*) C. Pettinari in *Scorpionates II: Chelating Borate Ligands*; Imperial College Press; River Edge NJ 2008.

2- (*a*) C. Mealli, C. S. Arcus, J. L. Wilkinson, T. J. Marks and J. A. Ibers, *J. Am. Chem. Soc.* 1976, **98**, 711-718; (*b*) J. L. Schneider, S. M. Carrier, C. E. Ruggiero, V. G. Jr.Young and W. B. Tolman, *J. Am. Chem. Soc.* 1998, **120**, 11408-11418; (*c*) M. A. Mairena, J. Urbano, J. Carbajo, J. J. Maraver, E. Alvarez, M. M. Díaz-Requejo and P. J. Pérez, *Inorg. Chem.* 2007, **46**, 7428-7435.

3- *(a)* C. Lee, W. Yang, R. G. Parr, *Phys. Rev. B*, 1988, **37**, 785-789; *(b)* A. D. Becke, *Phys. Rev. A*, 1988, **38**, 3098-3100.

4- *(a)* J. Poater. M. Solà, A. Rimola, L. Rodríguez-Santiago, and M. Sodupe, *J. Phys. Chem. A*, 2004, **108**, 6072-6078; *(b)* R. Rios-Font, M. Sodupe, L. Rodríguez-Santiago and P. R. Taylor, *J. Phys. Chem. A*, 2010, **114**, 10857-10863.

5- D. Andrae, U. Häußermann, M. Dolg, H. Stoll and H. Preuß, *Theor. Chim. Acta*, 1990, **77**, 123-141.

6- A. V. Marenich, C. J. Cramer and D. G. Truhlar, *J. Phys. Chem. B*, 2009, **113**, 6378-6396.

7- (*a*) A. Ben-Naim and Y. Marcus, *J. Chem. Phys.,* 1984, **81**, 2016-2027; (*b*) V. S. Bryantsev, M. S. Diallo, W. A. Goddard III, *J. Phys. Chem. B,* 2008, **112**, 9709-9719.

8- A.E. Reed, L. A. Curtiss and F. Weinhold, *Chem. Rev.,* 1988, **88**, 899-926.

9- J. N. Harvey, M. Aschi, H. Schwarz, W. Koch, *Theor. Chem. Acc.*, 1998, **99**, 95-99.

15. XYZ coordinates of all the optimized structures.

-H

CuOTp*,Br

Cu	-0.000343	-0.000237	1.956367
B	0.000397	-0.000272	-1.101322
H	0.000602	-0.000035	-2.287690
C	2.142833	-1.502792	-1.237596
C	2.959461	-2.075892	-0.283982
C	2.440787	-1.713084	0.963009
N	1.372951	-0.964383	0.760289
N	1.188591	-0.834110	-0.570922
N	-1.520749	-0.708242	0.759859
C	-2.703365	-1.258276	0.962243
C	-3.277272	-1.524432	-0.284860
C	-2.372434	-1.103463	-1.238183
N	-1.315922	-0.612455	-0.571309
N	0.148600	1.670539	0.760236
C	0.263337	2.969707	0.962852
C	0.317905	3.600197	-0.284177
C	0.229437	2.606322	-1.237668
N	0.127895	1.445649	-0.570932
O	-0.000556	0.002585	3.664482
C	2.933446	-2.059192	2.321730
H	2.947912	-3.136424	2.463255
H	3.948105	-1.698459	2.466872
H	2.295632	-1.617246	3.079207
C	2.232580	-1.564329	-2.720871
H	1.339568	-2.005720	-3.153255
H	2.354659	-0.574611	-3.150950
H	3.084803	-2.168239	-3.007701
C	-2.471456	-1.149056	-2.721530
H	-1.670695	-1.741848	-3.153585
H	-3.416950	-1.593184	-3.008054
H	-2.417046	-0.153770	-3.152630
C	-3.249781	-1.512689	2.320697
H	-4.186502	-0.981172	2.464605
H	-3.451258	-2.570943	2.462637
H	-2.545722	-1.187992	3.078798
C	0.237626	2.714955	-2.720925
H	1.069458	2.166805	-3.153275
H	-0.678316	2.321255	-3.151492
H	0.329112	3.755543	-3.007366
C	0.317398	3.569539	2.321472
H	1.244536	4.118118	2.463414
H	-0.500440	4.270219	2.465817
H	0.251063	2.796603	3.079124
Br	0.480804	5.444454	-0.581332
Br	-4.956378	-2.304455	-0.582077
Br	4.475522	-3.138578	-0.580858

C_6H_6

C	-1.756066	0.322303	0.000062
C	-0.369365	0.322217	0.000540
C	0.324018	1.522979	-0.000049
C	-0.369304	2.723896	-0.001027
C	-1.755903	2.723982	-0.001468
C	-2.449337	1.523131	-0.000956
H	-2.295040	-0.611321	0.000476
H	0.169507	-0.611465	0.001269
H	1.402043	1.523014	0.000255

H	0.169784	3.657455	-0.001479
H	-2.294884	3.657601	-0.002229
H	-3.527361	1.523225	-0.001304

T-I1

Cu	0.287444	-0.034663	-1.317993
B	-1.143532	-0.167240	1.362550
H	-1.675752	-0.238733	2.420001
C	-3.251118	-1.369319	0.373217
C	-3.596532	-1.784920	-0.896438
C	-2.515000	-1.480235	-1.730149
N	-1.575290	-0.921038	-0.993483
N	-2.016043	-0.848559	0.282773
N	0.993585	-0.941737	0.302123
C	2.101860	-1.606424	0.585816
C	2.040169	-1.986299	1.927534
C	0.839595	-1.514638	2.423486
N	0.226327	-0.884901	1.412408
N	-0.265192	1.621667	-0.162451
C	-0.257508	2.933310	-0.298363
C	-0.911544	3.489046	0.806465
C	-1.316521	2.436088	1.601119
N	-0.908738	1.313776	0.985860
O	0.431520	0.463791	-3.048494
C	4.785048	-0.384124	-3.588637
C	5.962586	-0.420842	-2.856401
C	6.167439	0.483756	-1.824646
C	5.194397	1.426783	-1.527601
C	4.017320	1.461871	-2.261003
C	3.808395	0.555939	-3.291751
H	4.627375	-1.087467	-4.390555
H	6.720740	-1.150996	-3.090339
H	7.084130	0.456983	-1.257653
H	5.355120	2.134143	-0.729784
H	3.261903	2.197135	-2.034376
H	2.880634	0.575500	-3.839481
C	-4.032464	-1.442678	1.636744
H	-4.240128	-0.452561	2.032341
H	-4.977499	-1.938036	1.450041
H	-3.498453	-1.999659	2.400675
C	-2.352189	-1.705185	-3.190507
H	-2.188951	-2.758423	-3.406097
H	-3.246186	-1.397874	-3.724243
H	-1.510615	-1.133172	-3.566696
C	0.365379	3.608595	-1.466974
H	-0.289273	4.385078	-1.850379
H	1.303810	4.083014	-1.188957
H	0.550159	2.891241	-2.259676
C	-2.061919	2.455973	2.888132
H	-2.226096	3.481501	3.195845
H	-3.029494	1.971201	2.793592
H	-1.511124	1.946498	3.672892
C	3.173549	-1.854727	-0.412540
H	2.862274	-1.549099	-1.404737
H	4.073386	-1.299169	-0.164404
H	3.431668	-2.909275	-0.436976
C	0.267863	-1.639544	3.790603
H	0.086451	-0.664410	4.232238
H	-0.674783	-2.178378	3.776073
H	0.960235	-2.179039	4.425166
Br	3.348175	-2.952894	2.857769
Br	-5.199515	-2.604456	-1.421130
Br	-1.175953	5.317237	1.129739

T-TS1

Cu	-0.561990	0.479072	1.096711
B	0.799127	-0.616653	-1.447209
H	1.275604	-0.998004	-2.464556
C	2.296079	-2.384739	-0.223129
C	2.445486	-2.730782	1.102447
C	1.562251	-1.924016	1.833779
N	0.921567	-1.143473	0.988991
N	1.361856	-1.417062	-0.258611
N	-1.490279	-0.401097	-0.442476
C	-2.743588	-0.759167	-0.668806
C	-2.792609	-1.393080	-1.911322
C	-1.503196	-1.402622	-2.406272
N	-0.736207	-0.793695	-1.491630
N	0.665457	1.546645	-0.162223
C	1.209520	2.751407	-0.154044
C	2.023361	2.871181	-1.282741
C	1.949310	1.668692	-1.955825
N	1.118443	0.885495	-1.251805
O	-0.739354	0.869682	2.849511
C	-2.518439	1.812083	4.425446
C	-3.858927	1.598525	4.226869
C	-4.475074	2.013302	3.040788
C	-3.726682	2.656919	2.059670
C	-2.376483	2.879583	2.247366
C	-1.711246	2.391140	3.403917
H	-2.043820	1.501477	5.340768
H	-4.447303	1.124888	4.996133
H	-5.531301	1.853651	2.897354
H	-4.207968	3.008823	1.161103
H	-1.815649	3.428257	1.509456
H	-0.785863	2.854733	3.694744
C	2.986094	-2.921421	-1.426798
H	3.548660	-2.147559	-1.941420
H	3.676523	-3.702071	-1.131001
H	2.278921	-3.342625	-2.135532
C	1.313491	-1.875519	3.298907
H	0.903393	-2.818472	3.652239
H	2.240605	-1.703178	3.838999
H	0.616774	-1.076291	3.529963
C	0.964907	3.743442	0.925074
H	1.888700	4.252515	1.180695
H	0.252778	4.503120	0.610799
H	0.586651	3.245944	1.809888
C	2.631481	1.246645	-3.208422
H	3.187969	2.081831	-3.615964
H	3.326058	0.431955	-3.025353
H	1.917109	0.912324	-3.954120
C	-3.845085	-0.507499	0.295258
H	-3.465437	-0.088858	1.219477
H	-4.573151	0.183674	-0.120994
H	-4.366521	-1.433621	0.519783
C	-0.986665	-1.961413	-3.684035
H	-0.473074	-1.203182	-4.266759
H	-0.287704	-2.773332	-3.505555
H	-1.811695	-2.345761	-4.271328
Br	-4.326652	-2.092310	-2.729927
Br	3.601736	-4.028829	1.809897
Br	3.021398	4.381392	-1.771341

T-I2

Cu	0.474694	0.335000	-1.180317
B	-0.971711	-0.266161	1.460110
H	-1.500282	-0.435179	2.508692
C	-2.324886	-2.327391	0.578343
C	-2.389668	-2.951380	-0.648852
C	-1.527162	-2.252456	-1.504814

N	-0.980464	-1.266305	-0.824048
N	-1.460101	-1.306617	0.438392
N	1.382248	-0.166471	0.566247
C	2.627416	-0.384257	0.954146
C	2.603523	-0.742585	2.304022
C	1.280620	-0.732153	2.697713
N	0.562508	-0.377704	1.622103
N	-0.771486	1.579148	-0.233499
C	-1.260032	2.777791	-0.506013
C	-2.127705	3.134061	0.527018
C	-2.134478	2.081629	1.422946
N	-1.301210	1.153968	0.933082
O	1.069586	0.148020	-2.879790
C	3.185301	-0.287274	-3.890479
C	4.505384	-0.029107	-3.705700
C	4.943070	1.216948	-3.203321
C	3.991343	2.198156	-2.849409
C	2.661131	1.980823	-3.021198
C	2.106817	0.721783	-3.621161
H	2.851301	-1.245886	-4.253842
H	5.236618	-0.790331	-3.929692
H	5.993998	1.407139	-3.064771
H	4.332058	3.132636	-2.430600
H	1.945600	2.744910	-2.758328
H	1.674871	0.994750	-4.597866
C	-3.030864	-2.653228	1.846462
H	-3.648089	-1.824905	2.182437
H	-3.670900	-3.513845	1.694240
H	-2.329735	-2.887356	2.642497
C	-1.212064	-2.493546	-2.937802
H	-0.824635	-3.499007	-3.080688
H	-2.106768	-2.402438	-3.548655
H	-0.473267	-1.775876	-3.278116
C	-0.892663	3.539299	-1.728598
H	-1.773354	3.997196	-2.167337
H	-0.191212	4.336866	-1.495193
H	-0.441663	2.883795	-2.465296
C	-2.892152	1.928825	2.693514
H	-3.460845	2.830144	2.886920
H	-3.583697	1.093037	2.641853
H	-2.224921	1.756418	3.532226
C	3.794405	-0.246780	0.046234
H	3.480551	-0.224653	-0.989550
H	4.343685	0.667375	0.256625
H	4.478036	-1.078524	0.185388
C	0.689479	-1.046196	4.025946
H	0.124500	-0.205280	4.416372
H	0.018740	-1.898290	3.967915
H	1.480430	-1.282544	4.727203
Br	4.094997	-1.154211	3.362603
Br	-3.427317	-4.447057	-1.104581
Br	-3.090441	4.736742	0.656111

MECP1

Cu	-0.486835	0.099630	-1.292092
B	1.113007	-0.049419	1.313829
H	1.734476	-0.084773	2.325356
C	2.688273	-1.970983	0.503633
C	2.783356	-2.686162	-0.670257
C	1.788459	-2.191291	-1.524343
N	1.137614	-1.236306	-0.895285
N	1.678101	-1.097469	0.332747
N	0.607134	1.669690	-0.454907
C	0.886502	2.922067	-0.754937
C	1.715966	3.443223	0.243938
C	1.919535	2.429414	1.156015

N	1.231379	1.367180	0.701997
N	-1.299381	-0.372434	0.646476
C	-2.455827	-0.695553	1.189198
C	-2.266426	-0.897700	2.560692
C	-0.928482	-0.679613	2.808735
N	-0.371111	-0.361855	1.626489
O	-1.433381	-0.094233	-3.004172
C	-3.365988	1.355424	-2.808758
C	-4.733571	1.469234	-2.810330
C	-5.528692	0.376307	-3.156586
C	-4.960471	-0.836102	-3.546743
C	-3.596153	-0.984950	-3.557927
C	-2.686906	0.129283	-3.249160
H	-2.727826	2.183106	-2.544511
H	-5.196183	2.398436	-2.522265
H	-6.601699	0.476228	-3.139009
H	-5.593924	-1.664481	-3.816515
H	-3.122080	-1.903479	-3.861960
H	-2.879132	0.487475	-4.368279
C	2.726890	2.433402	2.405555
H	3.174557	3.410319	2.544179
H	2.115283	2.210964	3.275028
H	3.523115	1.695829	2.365435
C	0.356758	3.583322	-1.976863
H	-0.111418	2.845471	-2.620114
H	-0.374019	4.348858	-1.723670
H	1.153670	4.070039	-2.531974
C	1.441182	-2.598439	-2.911471
H	2.284938	-2.457098	-3.582304
H	1.169371	-3.650474	-2.950861
H	0.606127	-2.004306	-3.267498
C	3.497893	-2.083132	1.746941
H	4.246289	-2.857037	1.623315
H	4.006063	-1.151013	1.976353
H	2.880113	-2.340636	2.602516
C	-3.708793	-0.801842	0.396380
H	-4.214722	-1.742905	0.593662
H	-4.402431	-0.001695	0.644966
H	-3.480892	-0.746892	-0.661946
C	-0.183576	-0.764173	4.093778
H	0.596113	-1.519101	4.052579
H	0.286867	0.182136	4.343561
H	-0.868336	-1.025971	4.891695
Br	-3.591696	-1.372240	3.805803
Br	4.001781	-4.060787	-1.067127
Br	2.406778	5.189045	0.311598

S-I3

Cu	-0.560823	0.000022	-1.210895
B	1.189832	-0.000176	1.241437
H	1.885683	-0.000292	2.203624
C	3.427750	0.000700	-0.100739
C	3.736243	0.001196	-1.443655
C	2.518486	0.001163	-2.134786
N	1.538704	0.000674	-1.255419
N	2.084133	0.000392	-0.020111
N	-0.572193	1.524608	0.275029
C	-1.158567	2.674940	0.537077
C	-0.647396	3.173368	1.740746
C	0.283219	2.257731	2.182019
N	0.304527	1.269135	1.269427
N	-0.571338	-1.525346	0.274071
C	-1.157212	-2.676073	0.535518
C	-0.645958	-3.174820	1.739016
C	0.284176	-2.258952	2.180825
N	0.305137	-1.269927	1.268694

O	-1.813619	0.000620	-2.844518
C	-3.890099	-0.000407	-1.742476
C	-5.226869	-0.000571	-1.877571
C	-5.890923	0.000266	-3.167790
C	-5.177190	0.001180	-4.292396
C	-3.687838	0.001299	-4.274621
C	-3.036728	0.000479	-2.914230
H	-3.408058	-0.001050	-0.779087
H	-5.843835	-0.001327	-0.992440
H	-6.967496	0.000224	-3.192837
H	-5.661773	0.001884	-5.255695
H	-3.302090	-0.861191	-4.820316
H	-3.302218	0.864553	-4.819186
C	1.129344	2.291404	3.405312
H	0.908205	3.187285	3.973291
H	0.944697	1.430093	4.040449
H	2.186710	2.297254	3.157449
C	-2.180726	3.272007	-0.362751
H	-2.279513	2.669658	-1.258873
H	-3.150160	3.334074	0.126174
H	-1.900099	4.280829	-0.653240
C	2.268324	0.001576	-3.600205
H	2.707228	0.877601	-4.070974
H	2.707235	-0.874177	-4.071477
H	1.200551	0.001630	-3.790697
C	4.339333	0.000517	1.075100
H	5.368082	0.000821	0.734929
H	4.185581	0.877113	1.697647
H	4.185923	-0.876521	1.697109
C	-2.178981	-3.273203	-0.364711
H	-1.897919	-4.281813	-0.655511
H	-3.148482	-3.335812	0.124012
H	-2.277825	-2.670579	-1.260643
C	1.130164	-2.292792	3.404209
H	2.187564	-2.297933	3.156479
H	0.944963	-1.431908	4.039765
H	0.909440	-3.189083	3.971702
Br	-1.143473	-4.783799	2.571828
Br	5.448665	0.001813	-2.216523
Br	-1.145560	4.781733	2.574355

CuTp^{*,Br}

Cu	-0.002833	-0.000701	2.024107
B	-0.001410	-0.000180	-0.822462
H	-0.001601	-0.000094	-2.008152
C	1.032921	2.385451	-1.065437
C	1.450677	3.348726	-0.172301
C	1.226884	2.833312	1.108946
N	0.705186	1.630077	0.988804
N	0.583927	1.351143	-0.330812
N	1.058601	-1.426568	0.988534
C	1.841575	-2.478699	1.108472
C	2.177345	-2.928984	-0.172855
C	1.550516	-2.086481	-1.065824
N	0.876866	-1.182424	-0.331037
N	-1.767081	-0.204396	0.988085
C	-3.069872	-0.355353	1.107741
C	-3.627503	-0.420012	-0.173740
C	-2.584152	-0.299178	-1.066451
N	-1.464362	-0.169386	-0.331392
Br	-5.448728	-0.631078	-0.575152
Br	3.272408	-4.399370	-0.574397
Br	2.180467	5.030642	-0.573547
C	1.497457	3.457941	2.430167
H	2.551906	3.696721	2.539569
H	0.940628	4.384126	2.544805

H	1.211446	2.781580	3.228589
C	1.046289	2.415911	-2.552704
H	1.463663	3.356835	-2.890504
H	1.647730	1.609390	-2.961277
H	0.044494	2.321080	-2.961095
C	-3.746970	-0.433644	2.428463
H	-4.476353	0.364126	2.540141
H	-4.276200	-1.376214	2.539632
H	-3.018163	-0.350025	3.227572
C	-2.617101	-0.303081	-2.553745
H	-3.639751	-0.421030	-2.891268
H	-2.227738	0.624625	-2.962391
H	-2.026837	-1.117906	-2.962277
C	2.247630	-3.025210	2.429631
H	1.815251	-2.431714	3.228103
H	1.916482	-4.053888	2.544442
H	3.328774	-3.017452	2.538777
C	1.570795	-2.112567	-2.553094
H	2.190934	-2.934068	-2.891049
H	0.573742	-2.247396	-2.961808
H	1.973657	-1.190442	-2.961196

C_6H_6O

O	-1.814283	0.001033	-2.823761
C	-3.894431	-0.000538	-1.741911
C	-5.226859	-0.000554	-1.871400
C	-5.888545	0.000202	-3.167782
C	-5.177864	0.001133	-4.293051
C	-3.686436	0.001216	-4.282557
C	-3.019798	-0.000286	-2.917605
H	-3.408208	-0.000893	-0.780822
H	-5.847927	-0.001052	-0.988778
H	-6.965716	0.000200	-3.193986
H	-5.669510	0.001920	-5.253509
H	-3.304526	-0.861495	-4.829860
H	-3.304632	0.865072	-4.828141

C_6H_5OH

C	-1.751534	1.214711	-0.002616
C	-0.365778	1.211252	-0.011452
C	0.324131	2.413534	-0.019880
C	-0.365787	3.615849	-0.012914
C	-1.751512	3.612416	-0.004062
C	-2.448058	2.413551	-0.001466
H	-2.286987	0.278957	0.005061
H	0.191869	0.289300	-0.002187
H	0.191935	4.537766	-0.004821
H	-2.286998	4.548161	0.002476
H	-3.525706	2.413577	0.006410
O	1.697014	2.413656	0.013074
H	2.039225	2.411461	-0.877270

T-TS2

Cu	0.563817	0.136509	-1.103835
B	-1.347994	-0.029888	1.285198
H	-2.045706	-0.058492	2.244239
C	-3.353708	-1.045516	-0.059709
C	-3.516574	-1.392320	-1.384010
C	-2.315067	-1.081649	-2.035160
N	-1.485066	-0.575340	-1.146389
N	-2.108197	-0.550900	0.052302
N	0.883064	-0.969341	0.583918
C	1.806754	-1.816260	1.003467
C	1.416034	-2.298361	2.255393

C	0.210023	-1.698864	2.556424
N	-0.088534	-0.896390	1.523629
N	-0.051251	1.682246	-0.010372
C	0.152212	2.987222	-0.075754
C	-0.579656	3.587116	0.949705
C	-1.231453	2.570361	1.622412
N	-0.889531	1.426075	1.015074
O	1.310391	-0.174653	-2.728114
C	3.750415	0.110764	-2.711429
C	4.349117	1.174163	-2.070333
C	5.737418	1.245534	-2.064104
C	6.487438	0.263099	-2.693102
C	5.858903	-0.795836	-3.332419
C	4.472034	-0.879249	-3.344333
H	2.432233	0.002571	-2.773742
H	3.761145	1.937080	-1.586459
H	6.230069	2.068021	-1.570285
H	7.563630	0.323381	-2.686137
H	6.446336	-1.556146	-3.822104
H	3.969655	-1.695601	-3.836740
C	-1.939186	-1.250481	-3.463729
H	-2.561874	-0.628504	-4.101951
H	-0.899267	-0.978310	-3.611181
H	-2.082052	-2.280983	-3.777299
C	-4.307802	-1.162905	1.075413
H	-5.244133	-1.576285	0.720745
H	-3.921210	-1.815147	1.853364
H	-4.510574	-0.195353	1.525361
C	1.024983	3.614199	-1.102817
H	0.495950	4.410991	-1.617063
H	1.907828	4.053292	-0.644899
H	1.339355	2.882153	-1.839316
C	-2.148718	2.646612	2.790361
H	-2.244213	3.677026	3.110134
H	-3.137134	2.273313	2.539485
H	-1.774274	2.061101	3.624382
C	3.023452	-2.143306	0.216408
H	2.956122	-1.735079	-0.784224
H	3.912998	-1.740302	0.693841
H	3.149878	-3.219706	0.147621
C	-0.649850	-1.862288	3.759014
H	-0.812675	-0.913496	4.260995
H	-1.621710	-2.270585	3.497681
H	-0.172159	-2.540672	4.455279
Br	2.365150	-3.524131	3.309387
Br	-5.044825	-2.135953	-2.178666
Br	-0.649927	5.421712	1.323883

T-I4

Cu	-0.412197	-0.057917	1.737334
B	-0.966308	-0.015146	-1.265733
H	-1.221877	-0.014154	-2.424081
C	0.017232	2.390198	-1.610676
C	0.590994	3.305941	-0.753180
C	0.562387	2.737628	0.526693
N	-0.000296	1.548346	0.432564
N	-0.332215	1.331810	-0.859797
N	0.351568	-1.436716	0.342036
C	1.220301	-2.430247	0.339839
C	1.438101	-2.819313	-0.985521
C	0.655551	-2.001046	-1.773755
N	0.006880	-1.171821	-0.940024
N	-2.202680	-0.251423	0.904466
C	-3.424224	-0.421977	1.380834
C	-4.299306	-0.503401	0.297174
C	-3.532661	-0.371114	-0.845662

N	-2.263649	-0.219634	-0.442964
O	0.580372	0.188797	3.226974
C	4.926430	-0.239858	2.767474
C	6.122135	-0.485312	2.144126
C	6.186614	-0.202861	0.781388
C	5.074761	0.301433	0.120757
C	3.888585	0.529608	0.805267
C	3.796857	0.257523	2.170514
H	0.941555	-0.597606	3.623917
H	6.976141	-0.876685	2.672655
H	7.103862	-0.376927	0.241039
H	5.133530	0.518323	-0.933475
H	3.027631	0.918988	0.285222
H	2.873984	0.426662	2.703960
C	1.049739	3.285862	1.819990
H	0.475226	4.163485	2.105958
H	0.968584	2.534441	2.597947
H	2.088797	3.591717	1.735069
C	-0.204580	2.477776	-3.078864
H	0.133729	3.441468	-3.439672
H	0.342950	1.704471	-3.610174
H	-1.255994	2.369061	-3.327712
C	-3.715001	-0.500663	2.836469
H	-4.427028	0.266353	3.127218
H	-4.149548	-1.463383	3.091134
H	-2.809473	-0.365364	3.419028
C	-3.953490	-0.381983	-2.271855
H	-5.025704	-0.522638	-2.332891
H	-3.701845	0.552755	-2.763650
H	-3.470115	-1.185897	-2.818625
C	1.827700	-2.970714	1.584189
H	1.230949	-2.696341	2.447005
H	1.894994	-4.052854	1.538783
H	2.830568	-2.575930	1.724580
C	0.504472	-1.980697	-3.253426
H	-0.528165	-2.140083	-3.548739
H	0.825980	-1.031253	-3.671767
H	1.110090	-2.765671	-3.689648
Br	2.575558	-4.192889	-1.564255
Br	1.277614	4.993880	-1.195971
Br	-6.154298	-0.745066	0.397725
Br	-6.154298	-0.745066	0.397725

T-I5

Cu	-0.252347	-0.155015	1.394852
B	0.687025	0.075069	-1.525496
H	1.066731	0.128515	-2.647772
C	2.508898	-1.771879	-1.156961
C	2.880384	-2.534118	-0.066279
C	2.044625	-2.165032	0.989291
N	1.218607	-1.231169	0.545550
N	1.497876	-0.991200	-0.752640
N	-1.433421	-0.548927	-0.319915
C	-2.678404	-0.902328	-0.585121
C	-2.849382	-0.897044	-1.973417
C	-1.641690	-0.526044	-2.523779
N	-0.801006	-0.322534	-1.496603
N	0.641506	1.596296	0.479863
C	0.936334	2.842122	0.806261
C	1.387168	3.505473	-0.339421
C	1.350897	2.589837	-1.368793
N	0.896067	1.441743	-0.840718
O	-0.839976	-1.660033	4.084121
C	-1.802172	-0.737282	3.789007
C	-3.103225	-0.899120	4.125159
C	-4.059954	0.103152	3.801905

C	-3.636301	1.253740	3.116240
C	-2.328743	1.432766	2.764466
C	-1.276005	0.425758	3.050583
H	-1.222265	-2.369116	4.596763
H	-3.418361	-1.794521	4.643198
H	-5.090279	-0.025873	4.084368
H	-4.359354	2.016590	2.870808
H	-2.033907	2.337332	2.256502
H	-0.456227	0.863119	3.635696
Br	1.939301	5.295464	-0.433657
Br	-4.427657	-1.324155	-2.892254
Br	4.240876	-3.820913	0.006385
C	2.025402	-2.670374	2.387078
H	1.964854	-3.755070	2.394950
H	2.940980	-2.394311	2.904263
H	1.180282	-2.271752	2.937197
C	3.066239	-1.759801	-2.535605
H	3.870638	-2.481540	-2.607832
H	2.308283	-2.016574	-3.269423
H	3.459953	-0.781131	-2.792870
C	0.802609	3.385421	2.183661
H	1.736796	3.837703	2.504413
H	0.039015	4.157898	2.223492
H	0.536996	2.603689	2.882807
C	1.724297	2.763651	-2.798212
H	2.059170	3.780572	-2.962852
H	2.525679	2.089246	-3.084670
H	0.880260	2.572698	-3.454379
C	-3.671604	-1.243028	0.463899
H	-4.452146	-0.489640	0.515229
H	-4.146201	-2.193659	0.236895
H	-3.204740	-1.303511	1.438061
C	-1.267723	-0.364516	-3.954515
H	-2.128366	-0.573500	-4.578184
H	-0.931514	0.646333	-4.165314
H	-0.469016	-1.044588	-4.235577

MECP2

Cu	-0.18653407	-0.16383234	1.41793012
B	0.71612047	0.05992571	-1.51256883
H	1.09174307	0.12076860	-2.63511553
C	2.53837423	-1.78810168	-1.15591439
C	2.92151261	-2.54578358	-0.06510895
C	2.10370267	-2.16501355	0.99952949
N	1.27639191	-1.22872324	0.55980109
N	1.53885471	-0.99925050	-0.74330480
N	-1.38816711	-0.59597530	-0.29732388
C	-2.63865179	-0.93556352	-0.55621893
C	-2.82229987	-0.90559129	-1.94318258
C	-1.61859189	-0.53261110	-2.49933636
N	-0.76648154	-0.35177143	-1.47761016
N	0.67350779	1.56440874	0.50515848
C	0.93851428	2.81605221	0.83831811
C	1.36374673	3.49508492	-0.30750961
C	1.34067054	2.58493366	-1.34257572
N	0.91897017	1.42308236	-0.81902066
O	-0.95254714	-1.75376085	3.68172107
C	-1.86806701	-0.72750447	3.64408936
C	-3.08911505	-0.80016892	4.20325436
C	-4.01846679	0.27761077	4.03396254
C	-3.68545153	1.33186457	3.15612209
C	-2.46583001	1.40825816	2.55401516
C	-1.33924267	0.46350446	2.90557724
H	-1.32169905	-2.50012109	4.15049272
H	-3.39455988	-1.69053962	4.73693909
H	-4.98805805	0.22844595	4.49792589

H	-4.43687710	2.07360322	2.92642317
H	-2.26549150	2.19405471	1.84234329
H	-0.69342410	0.98818422	3.63193686
Br	1.87303276	5.29721149	-0.39595050
Br	-4.41111013	-1.30878831	-2.85379728
Br	4.27523137	-3.83880713	-0.00521318
C	2.10477639	-2.66011533	2.40126179
H	2.04511122	-3.74465509	2.41662066
H	3.02773803	-2.38022067	2.90267731
H	1.26599327	-2.26168638	2.96074651
C	3.07403185	-1.78485407	-2.54326713
H	3.87498586	-2.50900199	-2.62558862
H	2.30318110	-2.04212770	-3.26310470
H	3.46527678	-0.80813193	-2.81093457
C	0.79695630	3.34918035	2.21850426
H	1.72351237	3.81621762	2.54038455
H	0.01969668	4.10751148	2.25924634
H	0.54073710	2.56057478	2.91289384
C	1.69744168	2.77455942	-2.77409067
H	2.02079911	3.79588108	-2.93415918
H	2.50201374	2.10964329	-3.07335900
H	0.84842949	2.58117521	-3.42317842
C	-3.63313813	-1.28584345	0.48924806
H	-3.17100495	-1.35976209	1.46458494
H	-4.40791737	-0.52732802	0.54874190
H	-4.11269060	-2.23012427	0.24660612
C	-1.25783781	-0.35301874	-3.93127367
H	-2.12294363	-0.55862248	-4.54974255
H	-0.92794403	0.66149910	-4.13461793
H	-0.45897862	-1.02716305	-4.22614696

S-I6

Cu	-0.264566	-0.049599	1.395884
B	0.374617	0.120473	-1.518653
H	0.605850	0.179344	-2.680507
C	0.420429	-2.448265	-1.900187
C	0.246450	-3.546933	-1.088872
C	0.002546	-3.060457	0.200152
N	0.028675	-1.742194	0.165014
N	0.282341	-1.367387	-1.110997
N	-1.526982	0.852151	-0.022592
C	-2.694984	1.458825	-0.116208
C	-2.891448	1.846940	-1.445479
C	-1.780563	1.432295	-2.146498
N	-0.972257	0.831298	-1.255498
N	1.556517	0.857169	0.592331
C	2.674432	1.462785	0.947516
C	3.374781	1.829288	-0.206610
C	2.619238	1.403341	-1.276623
N	1.524686	0.818274	-0.758198
O	-0.585680	-2.383276	4.506609
C	-0.962728	-1.098830	4.337606
C	-2.188443	-0.616350	4.758874
C	-2.488108	0.732917	4.607783
C	-1.581089	1.598109	4.030105
C	-0.349765	1.109388	3.596676
C	-0.032557	-0.239374	3.739948
H	-1.292402	-2.890057	4.899337
H	-2.898665	-1.281844	5.225295
H	-3.440829	1.100442	4.952671
H	-1.813951	2.643292	3.920944
H	0.385547	1.790749	3.205715
H	0.953100	-0.619595	3.526104
Br	5.025496	2.723257	-0.268608
Br	-4.391094	2.751303	-2.123477
Br	0.318051	-5.354735	-1.592560

C	-0.255789	-3.837871	1.439446
H	-1.197831	-4.377081	1.365746
H	0.525107	-4.576984	1.597353
H	-0.293154	-3.187315	2.303613
C	0.707070	-2.396339	-3.359291
H	0.766821	-3.404937	-3.750629
H	-0.071815	-1.866553	-3.899609
H	1.649093	-1.895707	-3.562590
C	3.071813	1.687239	2.364130
H	4.139778	1.537048	2.485832
H	2.851809	2.704115	2.683424
H	2.556867	0.999939	3.024669
C	2.902122	1.527256	-2.732234
H	3.836744	2.056789	-2.873683
H	2.987343	0.553654	-3.205697
H	2.118193	2.076077	-3.245364
C	-3.606810	1.647852	1.041440
H	-3.253101	1.082905	1.894155
H	-3.671912	2.696434	1.323565
H	-4.611430	1.317792	0.792281
C	-1.474980	1.583038	-3.594760
H	-2.278276	2.126535	-4.077704
H	-0.550398	2.130515	-3.751128
H	-1.374118	0.618438	-4.083357

-CF3

$C_6H_5CF_3$

C	-1.676546	1.311420	-0.027687
C	-0.289797	1.305623	-0.017338
C	0.400756	2.505356	0.003006
C	-0.293005	3.705530	0.013521
C	-1.679182	3.707374	0.001737
C	-2.373936	2.509972	-0.018569
H	0.241726	0.369468	-0.033507
H	1.478164	2.503147	0.007428
H	0.246224	4.638619	0.027694
H	-3.450391	2.503032	-0.035618
H	-2.219063	4.639763	0.005169
C	-2.424179	0.016476	0.005051
F	-1.751001	-0.961661	-0.602384
F	-2.650716	-0.391732	1.257050
F	-3.614214	0.114012	-0.589135

T-I1

Cu	-0.252525	-0.018740	-1.256680
B	-1.805455	-0.172576	1.353117
H	-2.384748	-0.250140	2.384969
C	-3.863128	-1.374669	0.263988
C	-4.148358	-1.789024	-1.020885
C	-3.030169	-1.480091	-1.802968
N	-2.126891	-0.919204	-1.023189
N	-2.626742	-0.850108	0.231426
N	0.383563	-0.930744	0.390001
C	1.474827	-1.602648	0.720468
C	1.346018	-2.000471	2.051925
C	0.122993	-1.532821	2.493310
N	-0.438219	-0.887816	1.461538
N	-0.871055	1.626045	-0.126409
C	-0.868433	2.938177	-0.259944
C	-1.570485	3.486795	0.818246
C	-1.999316	2.428922	1.594023
N	-1.558330	1.311073	0.994115
O	-0.021224	0.489079	-2.976228

C	4.205517	-0.400543	-3.528468
C	5.250879	-0.521430	-2.627242
C	5.339756	0.356049	-1.557001
C	4.398327	1.362051	-1.392768
C	3.355747	1.477001	-2.296247
C	3.251154	0.591385	-3.359936
H	4.133274	-1.083224	-4.358956
H	4.484191	2.046330	-0.565678
H	2.620731	2.254806	-2.173007
H	2.415164	0.667705	-4.033768
C	-4.701494	-1.453154	1.490056
H	-4.929653	-0.464628	1.878223
H	-5.635645	-1.950317	1.258784
H	-4.201897	-2.010965	2.276310
C	-2.800401	-1.703166	-3.254537
H	-2.651542	-2.759127	-3.466860
H	-3.659191	-1.372777	-3.830833
H	-1.928148	-1.149993	-3.586218
C	-0.205657	3.619739	-1.402758
H	-0.838442	4.411477	-1.791277
H	0.732538	4.076019	-1.094582
H	-0.013289	2.909135	-2.199927
C	-2.796991	2.440478	2.849390
H	-2.979203	3.464046	3.153399
H	-3.757177	1.950921	2.713469
H	-2.275981	1.931179	3.654288
C	2.590907	-1.855713	-0.226772
H	2.418436	-1.361132	-1.176392
H	3.531779	-1.496696	0.177743
H	2.699033	-2.922050	-0.407636
C	-0.515624	-1.674380	3.828760
H	-0.717061	-0.704874	4.274057
H	-1.457109	-2.211129	3.760516
H	0.143903	-2.223799	4.489258
Br	2.606352	-2.980867	3.030690
Br	-5.722767	-2.611774	-1.621250
Br	-1.862056	5.312045	1.133650
H	5.994182	-1.290437	-2.751936
C	6.403905	0.165449	-0.526011
F	6.766252	1.315401	0.041486
F	5.983240	-0.634367	0.465328
F	7.500123	-0.398600	-1.031735

T-TS1

Cu	0.299320	0.390759	-0.825092
B	-1.715469	-0.579528	1.288308
H	-2.451126	-0.935174	2.147916
C	-3.193450	-1.963787	-0.373700
C	-3.120336	-2.195024	-1.730537
C	-1.981828	-1.522617	-2.194890
N	-1.407546	-0.927635	-1.169836
N	-2.137430	-1.190519	-0.062823
N	0.731955	-0.746006	0.761624
C	1.827629	-1.357610	1.185085
C	1.510725	-2.046105	2.356487
C	0.169123	-1.825207	2.599823
N	-0.275366	-1.033628	1.614643
N	-0.980506	1.599842	0.264570
C	-1.339432	2.872117	0.241234
C	-2.347669	3.060339	1.189474
C	-2.586453	1.829634	1.765585
N	-1.743107	0.963641	1.183325
O	0.802481	0.962106	-2.474356
C	3.066747	1.552876	-3.483483
C	4.220846	1.028801	-2.969759
C	4.553061	1.241449	-1.623890

C	3.726266	2.004447	-0.810027
C	2.561180	2.541677	-1.322555
C	2.148751	2.259207	-2.650997
H	2.810722	1.396745	-4.517265
H	4.008480	2.194974	0.210861
H	1.953563	3.179238	-0.704570
H	1.449905	2.925629	-3.122340
C	-4.201625	-2.433637	0.614009
H	-4.724502	-1.600514	1.075178
H	-4.933221	-3.058375	0.116015
H	-3.740163	-3.014575	1.407198
C	-1.431451	-1.432096	-3.573164
H	-1.088955	-2.405007	-3.917309
H	-2.194988	-1.093131	-4.267842
H	-0.600253	-0.735108	-3.596141
C	-0.748461	3.866056	-0.692340
H	-1.522846	4.525001	-1.071713
H	-0.009980	4.490331	-0.194131
H	-0.284503	3.358131	-1.529119
C	-3.570412	1.457171	2.817051
H	-4.057613	2.350434	3.188624
H	-4.334765	0.791357	2.426589
H	-3.087872	0.956056	3.650050
C	3.125404	-1.291267	0.466530
H	2.997847	-0.873235	-0.524591
H	3.846508	-0.683131	1.005369
H	3.551516	-2.284653	0.367397
C	-0.685111	-2.334597	3.705399
H	-1.164404	-1.520742	4.240233
H	-1.464983	-2.991230	3.330934
H	-0.076039	-2.894109	4.404751
Br	2.693692	-3.073179	3.383865
Br	-4.305538	-3.211260	-2.771323
Br	-3.205639	4.679829	1.584562
H	4.887657	0.461655	-3.598180
C	5.804480	0.628735	-1.091621
F	6.886538	1.046676	-1.750212
F	5.993003	0.901689	0.200680
F	5.782157	-0.705441	-1.209180

T-I2

Cu	0.155587	0.263974	-0.933086
B	-1.920580	-0.180926	1.274432
H	-2.690634	-0.299393	2.168714
C	-3.352687	-1.939190	-0.034729
C	-3.241086	-2.492091	-1.292348
C	-2.110207	-1.917480	-1.888313
N	-1.578717	-1.068757	-1.032520
N	-2.327621	-1.077511	0.092440
N	0.556081	-0.467404	0.910538
C	1.630672	-0.920571	1.535637
C	1.249275	-1.315075	2.819706
C	-0.106916	-1.079993	2.924791
N	-0.500398	-0.564905	1.751076
N	-1.049618	1.672578	-0.182082
C	-1.267525	2.948374	-0.456692
C	-2.278511	3.404628	0.389472
C	-2.655333	2.331665	1.175164
N	-1.891137	1.295857	0.804167
O	1.058980	0.106686	-2.502489
C	3.234842	-0.606956	-3.131507
C	4.474776	-0.652223	-2.585956
C	4.958105	0.424334	-1.807547
C	4.143929	1.557764	-1.578005
C	2.900608	1.634091	-2.111661
C	2.299006	0.554032	-2.961719

H	2.857724	-1.435028	-3.709238
H	4.527836	2.360878	-0.970686
H	2.292845	2.508710	-1.940682
H	2.135832	0.984076	-3.961974
C	-4.366630	-2.189142	1.024536
H	-4.900529	-1.280010	1.285910
H	-5.087360	-2.917160	0.672365
H	-3.907787	-2.575282	1.930273
C	-1.527360	-2.150598	-3.236202
H	-1.272493	-3.199071	-3.367492
H	-2.241743	-1.892247	-4.013807
H	-0.633512	-1.548794	-3.360298
C	-0.518868	3.687573	-1.506785
H	-1.192439	4.319868	-2.076116
H	0.238860	4.331344	-1.066093
H	-0.036771	2.996411	-2.189145
C	-3.695579	2.257987	2.235389
H	-4.137176	3.236545	2.378741
H	-4.485425	1.563449	1.964733
H	-3.273159	1.929274	3.179715
C	2.975075	-0.970390	0.907366
H	2.901294	-0.862080	-0.167212
H	3.619624	-0.183394	1.288491
H	3.458031	-1.917197	1.127359
C	-1.020005	-1.329044	4.072170
H	-1.523560	-0.418603	4.382033
H	-1.781972	-2.060394	3.819003
H	-0.450221	-1.708288	4.911614
Br	2.379233	-2.025950	4.134912
Br	-4.373864	-3.767666	-2.073094
Br	-2.977248	5.142581	0.437164
H	5.103361	-1.515193	-2.730393
C	6.261486	0.303191	-1.101854
F	7.121014	-0.474306	-1.760505
F	6.846087	1.488784	-0.920577
F	6.114192	-0.237883	0.118447

MECP1

Cu	0.40809126	0.02475882	-0.81628028
B	-1.79568020	-0.23409766	1.25810147
H	-2.62815222	-0.33488956	2.09741998
C	-3.22865293	-1.93312411	-0.11402390
C	-3.09036302	-2.50009969	-1.36273435
C	-1.88460470	-2.02177384	-1.89026158
N	-1.33265767	-1.21771780	-1.00502998
N	-2.14463207	-1.15892644	0.07273086
N	0.68639379	-0.60583929	1.09161279
C	1.69551832	-1.04780470	1.82001419
C	1.21123100	-1.36825545	3.09057182
C	-0.14156129	-1.09897915	3.08006043
N	-0.42906254	-0.63865880	1.85253131
N	-0.89379544	1.60645902	-0.21501773
C	-1.12978132	2.87489773	-0.49052156
C	-2.14822974	3.33319953	0.35042075
C	-2.51747761	2.26506549	1.13974127
N	-1.73943406	1.23326760	0.77179320
O	1.33055056	0.05514848	-2.51577774
C	2.89958249	-0.26054999	-3.40680167
C	4.05820842	-0.52611844	-2.65033698
C	4.56219493	0.43888875	-1.83085902
C	3.94996227	1.73629369	-1.73253489
C	2.83104705	2.02250337	-2.41339722
C	2.13873512	0.98217800	-3.21879911
H	2.55655302	-0.95304333	-4.15208725
H	4.43720704	2.48971021	-1.13559937
H	2.42109302	3.01752966	-2.39507101

H	1.62055639	1.38419917	-4.08335549
C	-4.31936668	-2.09806515	0.88400904
H	-4.81709588	-1.15495753	1.09024133
H	-5.05664160	-2.79530557	0.50388305
H	-3.93858074	-2.48317726	1.82526275
C	-1.25522460	-2.30389616	-3.20714133
H	-1.02148657	-3.36083337	-3.30862527
H	-1.92964216	-2.04238778	-4.01874499
H	-0.34343569	-1.72610723	-3.30881164
C	-0.39905976	3.61845487	-1.54889509
H	-1.08304323	4.23912132	-2.11931092
H	0.35826047	4.27321620	-1.12263965
H	0.08085095	2.92069784	-2.22470789
C	-3.56159884	2.19412748	2.19683501
H	-4.00744701	3.17270916	2.32932205
H	-4.34861213	1.49453445	1.93093628
H	-3.14376705	1.87652611	3.14715224
C	3.08089481	-1.14879294	1.29771839
H	3.09009620	-0.99833302	0.22451417
H	3.72818236	-0.40482434	1.75197112
H	3.50301351	-2.12553614	1.51612415
C	-1.14446141	-1.26454594	4.16531149
H	-1.63302343	-0.32389222	4.40113461
H	-1.91556124	-1.97627254	3.88519467
H	-0.65279610	-1.62830320	5.05956528
Br	-4.27319737	-3.68860357	-2.20747754
Br	-2.86672827	5.06707476	0.37974150
H	4.55119225	-1.47730958	-2.74465429
C	5.81788614	0.21515706	-1.05317865
F	6.31511595	-1.00543682	-1.22606701
F	6.76187752	1.09024589	-1.40622957
F	5.61581682	0.38562138	0.25723030

S-I3

Cu	-0.098530	0.000041	-1.276123
B	-1.895804	-0.000441	1.137001
H	-2.628144	-0.000651	2.070930
C	-4.083264	-0.000834	-0.284735
C	-4.348605	-0.000967	-1.636794
C	-3.110055	-0.000996	-2.288839
N	-2.158540	-0.000909	-1.378133
N	-2.743098	-0.000804	-0.159017
N	-0.140623	-1.560786	0.214392
C	0.373861	-2.748919	0.464508
C	-0.181050	-3.236754	1.652902
C	-1.068681	-2.279122	2.093667
N	-1.020224	-1.275492	1.197958
N	-0.141927	1.561139	0.214072
C	0.371846	2.749592	0.464120
C	-0.183148	3.237037	1.652638
C	-1.070057	2.278808	2.093563
N	-1.021130	1.275243	1.197811
O	1.578044	0.000843	-2.470333
C	3.826102	0.000638	-3.080683
C	5.105328	0.000575	-2.681362
C	5.480831	0.000763	-1.277112
C	4.553552	0.001033	-0.321855
C	3.104552	0.001164	-0.645580
C	2.748055	0.000886	-2.108214
H	3.549494	0.000479	-4.120920
H	4.837595	0.001178	0.717118
H	2.610882	-0.853575	-0.180681
H	2.611178	0.856318	-0.181130
C	-5.034013	-0.000767	0.859567
H	-4.901888	0.876229	1.486393
H	-6.050386	-0.000749	0.484316

H	-4.901936	-0.877731	1.486451
C	-2.816923	-0.001139	-3.745984
H	-3.241607	-0.877238	-4.229146
H	-3.242036	0.874608	-4.229403
H	-1.744369	-0.000903	-3.907367
C	1.358594	3.400867	-0.439590
H	1.026878	4.398034	-0.714992
H	2.330180	3.510007	0.038180
H	1.472449	2.818565	-1.347731
C	-1.940325	2.288507	3.300259
H	-1.745528	3.183555	3.878977
H	-2.992763	2.279608	3.031754
H	-1.754086	1.425193	3.931745
C	1.361212	-3.399537	-0.439017
H	1.474737	-2.817245	-1.347205
H	2.332825	-3.507886	0.038875
H	1.030277	-4.396979	-0.714357
C	-1.939269	-2.289489	3.300126
H	-1.754509	-1.425579	3.931218
H	-2.991644	-2.282305	3.031301
H	-1.743323	-3.183962	3.879347
Br	0.215147	-4.882141	2.466958
Br	-6.034421	-0.001113	-2.464096
Br	0.212141	4.882661	2.466660
H	5.896451	0.000372	-3.414047
C	6.941502	0.000615	-0.965783
F	7.540807	1.070750	-1.491255
F	7.180882	0.000903	0.340639
F	7.540476	-1.069967	-1.490723

$C_6H_5(CF_3)O$

O	1.912547	-0.000281	-2.619626
C	4.044774	-0.001600	-3.594120
C	5.366497	-0.001554	-3.401409
C	5.952629	0.000170	-2.065618
C	5.189954	0.001839	-0.976065
C	3.704139	0.002099	-1.065926
C	3.109503	0.000134	-2.463467
H	3.609145	-0.002937	-4.578812
H	5.640638	0.003070	0.002317
H	3.298781	-0.860238	-0.535732
H	3.299327	0.866390	-0.538489
H	6.038625	-0.002845	-4.244536
C	7.442029	-0.000077	-1.982553
F	7.957343	1.069240	-2.594000
F	7.883831	0.001613	-0.728218
F	7.956819	-1.071395	-2.590930

$C_6H_4(CF_3)OH$

C	-1.665317	1.301985	-0.039709
C	-0.276306	1.266133	-0.003042
C	0.454030	2.434466	-0.047188
C	-0.204957	3.656599	-0.128043
C	-1.592045	3.697728	-0.166476
C	-2.317407	2.520452	-0.121012
H	0.232625	0.318555	0.051939
H	1.530027	2.424898	-0.024004
H	-3.392722	2.551574	-0.156762
H	-2.103769	4.645395	-0.234801
C	-2.441894	0.032199	0.060282
F	-1.846499	-0.965665	-0.597322
F	-2.576538	-0.375968	1.326405
F	-3.674423	0.160390	-0.435208
O	0.556859	4.768559	-0.170147

H	0.006149	5.545367	-0.224738

T-TS2

Cu	0.026469	0.125117	-0.855387
B	-2.358125	0.005268	1.057322
H	-3.249541	-0.006775	1.839646
C	-4.043614	-0.958172	-0.701819
C	-3.921219	-1.297596	-2.032454
C	-2.600182	-1.013723	-2.403361
N	-1.972910	-0.529228	-1.350979
N	-2.841932	-0.493487	-0.316451
N	-0.050935	-0.990624	0.855625
C	0.733514	-1.867092	1.458293
C	0.064467	-2.343857	2.588185
C	-1.161337	-1.710546	2.623417
N	-1.203103	-0.894021	1.559791
N	-0.767212	1.685146	0.088347
C	-0.519367	2.984319	0.081957
C	-1.439932	3.600903	0.929661
C	-2.249615	2.600345	1.434602
N	-1.814943	1.449131	0.904917
O	1.100253	-0.245964	-2.274907
C	3.496624	-0.038689	-1.777456
C	4.002158	1.028690	-1.067263
C	5.363205	1.058797	-0.796534
C	6.177381	0.027327	-1.240763
C	5.646209	-1.037191	-1.956300
C	4.287013	-1.074365	-2.229548
H	2.201931	-0.107721	-2.096405
H	3.363762	1.826374	-0.725319
H	5.789120	1.874933	-0.237796
H	3.857974	-1.894441	-2.780692
C	-1.926284	-1.187956	-3.717120
H	-2.374649	-0.542901	-4.468676
H	-0.871280	-0.949909	-3.630779
H	-2.029118	-2.211703	-4.066251
C	-5.225284	-1.056187	0.196239
H	-6.070950	-1.444284	-0.358410
H	-5.033868	-1.721401	1.033455
H	-5.498529	-0.085730	0.600230
C	0.572574	3.590843	-0.723983
H	0.191998	4.411265	-1.324891
H	1.351078	3.993496	-0.080691
H	1.012910	2.856174	-1.390228
C	-3.396425	2.698279	2.376192
H	-3.528493	3.729107	2.681195
H	-4.317234	2.358576	1.911433
H	-3.229922	2.095586	3.263599
C	2.083591	-2.225611	0.952843
H	2.228799	-1.846944	-0.051182
H	2.859804	-1.814866	1.593702
H	2.207804	-3.304171	0.941234
C	-2.268953	-1.854953	3.605566
H	-2.511929	-0.904329	4.070306
H	-3.170193	-2.233051	3.131991
H	-1.975320	-2.550675	4.382022
Br	0.724277	-3.601451	3.811358
Br	-5.254521	-2.002482	-3.147857
Br	-1.540486	5.434877	1.298109
H	6.290232	-1.833737	-2.289188
C	7.651398	0.086066	-0.993112
F	8.300659	0.670036	-2.003530
F	7.943371	0.782017	0.106805
F	8.178625	-1.130848	-0.849717

T-I4

Cu	0.288018	0.044418	-0.959582
B	-1.883913	-0.052404	1.196761
H	-2.703719	-0.047568	2.053845
C	-3.816645	-0.725064	-0.432300
C	-3.889260	-0.903729	-1.797665
C	-2.608059	-0.658298	-2.304244
N	-1.814280	-0.352259	-1.296804
N	-2.543799	-0.390546	-0.159159
N	0.292269	-1.192093	0.718588
C	1.063874	-2.158063	1.179336
C	0.462434	-2.681149	2.329511
C	-0.708837	-1.978071	2.523677
N	-0.783005	-1.079473	1.528516
N	-0.282096	1.593610	0.159599
C	0.070592	2.866744	0.244022
C	-0.678736	3.454283	1.263842
C	-1.497654	2.467583	1.777955
N	-1.234856	1.350197	1.087640
O	1.199313	-0.805639	-2.280158
C	6.583302	-1.770197	-2.301793
C	7.091625	-0.705378	-1.608020
C	6.191356	0.295484	-1.254322
C	4.855616	0.174209	-1.608761
C	4.384491	-0.928992	-2.315815
C	5.273747	-1.935076	-2.674266
H	0.671051	-1.439015	-2.755010
H	8.133405	-0.632399	-1.344099
H	3.332466	-0.995895	-2.559101
H	4.936647	-2.800728	-3.219805
Br	-0.581758	5.242470	1.816062
Br	1.135136	-4.069592	3.396352
Br	-5.401776	-1.376182	-2.801546
C	-2.135520	-0.700741	-3.713891
H	-2.282610	-1.689084	-4.141787
H	-2.692406	0.002009	-4.327830
H	-1.082746	-0.447556	-3.766933
C	-4.883724	-0.856954	0.595699
H	-5.814348	-1.138009	0.117749
H	-4.634930	-1.617236	1.330275
H	-5.040780	0.077353	1.126536
C	1.099558	3.486589	-0.630492
H	0.795179	4.489132	-0.912364
H	2.053878	3.562280	-0.116292
H	1.251570	2.904144	-1.530666
C	-2.500042	2.551346	2.873416
H	-2.498264	3.551329	3.289511
H	-3.500250	2.333676	2.510801
H	-2.274430	1.847916	3.668845
C	2.338154	-2.540129	0.516654
H	3.190434	-2.114636	1.041131
H	2.457866	-3.619086	0.518956
H	2.341517	-2.178544	-0.504926
C	-1.736017	-2.126867	3.589128
H	-1.425982	-2.895742	4.286326
H	-1.873364	-1.200801	4.139226
H	-2.698798	-2.412113	3.174752
H	6.528725	1.161116	-0.710374
C	3.878978	1.241411	-1.241012
F	4.453078	2.278270	-0.628226
F	3.234052	1.722479	-2.305000
F	2.926353	0.789740	-0.399711

T-TS3

Cu	0.045945	0.068479	-1.202849

B	-1.585112	-0.106127	1.365192
H	-2.224272	-0.123260	2.362816
C	-3.764774	-0.873481	0.149427
C	-4.097664	-1.053812	-1.176839
C	-2.957717	-0.748445	-1.927846
N	-1.994406	-0.404902	-1.089693
N	-2.482225	-0.477961	0.169260
N	0.529406	-1.156344	0.508500
C	1.456244	-2.015147	0.897977
C	1.107189	-2.499175	2.162895
C	-0.074354	-1.882555	2.513303
N	-0.399675	-1.074754	1.491154
N	-0.328558	1.630679	0.016738
C	-0.088937	2.937445	0.029853
C	-0.651234	3.471341	1.188289
C	-1.248663	2.423696	1.862189
N	-1.039975	1.325323	1.128434
O	0.381948	-0.978783	-2.752792
C	1.913020	-0.271828	-3.340847
C	2.963181	-1.204027	-3.425667
C	4.151842	-0.945807	-2.788389
C	4.321712	0.212157	-2.014777
C	3.299318	1.141931	-1.949555
C	2.095605	0.921137	-2.612483
H	0.471430	-1.921959	-2.614618
H	2.857771	-2.090946	-4.031534
H	3.462766	2.062021	-1.411635
H	1.391296	1.732194	-2.723859
Br	-0.611956	5.265869	1.727885
Br	2.075297	-3.742987	3.178278
Br	-5.757830	-1.593611	-1.860960
C	-2.791448	-0.758132	-3.405515
H	-3.236196	-1.654023	-3.828245
H	-3.299357	0.094265	-3.851285
H	-1.741092	-0.726244	-3.666305
C	-4.597966	-1.062413	1.366971
H	-5.589745	-1.389301	1.079325
H	-4.170315	-1.811391	2.026964
H	-4.692573	-0.138941	1.930457
C	0.638017	3.667703	-1.043036
H	0.569064	4.734157	-0.867493
H	1.689176	3.400966	-1.064464
H	0.209938	3.461335	-2.018975
C	-1.995952	2.438910	3.148236
H	-1.973805	3.437556	3.566624
H	-3.033234	2.151054	3.005892
H	-1.556280	1.755405	3.867521
C	2.652900	-2.345658	0.084315
H	3.523975	-1.793205	0.426452
H	2.882024	-3.403929	0.160956
H	2.494541	-2.094243	-0.956023
C	-0.884361	-2.032216	3.752073
H	-0.419585	-2.765243	4.400031
H	-0.956663	-1.094553	4.295346
H	-1.893699	-2.364849	3.529187
H	4.975918	-1.634607	-2.888039
C	5.573403	0.406338	-1.241766
F	6.645990	-0.055882	-1.891333
F	5.800892	1.691592	-0.956513
F	5.552613	-0.239466	-0.060578

T-I5

Cu	-0.027396	-0.232455	-1.219121
B	-1.461478	0.230113	1.448074
H	-2.044318	0.381765	2.469256

C	-3.694147	-0.758122	0.495233
C	-4.054015	-1.276577	-0.734089
C	-2.920085	-1.218736	-1.545652
N	-1.937126	-0.692641	-0.832176
N	-2.403097	-0.412884	0.402963
N	0.501118	-1.116042	0.621531
C	1.441448	-1.919773	1.087604
C	1.283024	-2.022721	2.473034
C	0.198542	-1.242003	2.810750
N	-0.257304	-0.705293	1.667638
N	-0.397283	1.692397	-0.303824
C	-0.127516	2.967697	-0.523593
C	-0.556919	3.708512	0.581535
C	-1.101674	2.810508	1.474219
N	-0.991947	1.597401	0.908922
O	0.588812	-1.679242	-3.994676
C	1.710187	-1.247058	-3.355846
C	2.896140	-1.898801	-3.424125
C	4.034777	-1.393969	-2.746891
C	3.903886	-0.204880	-2.000965
C	2.723630	0.472127	-1.927241
C	1.488104	-0.019708	-2.570626
H	0.791756	-2.450758	-4.519560
H	2.979768	-2.809319	-4.000119
H	2.676715	1.391667	-1.369791
H	1.044786	0.741625	-3.222076
Br	-0.417072	5.566986	0.786943
Br	2.349997	-3.038293	3.632623
Br	-5.733862	-1.935904	-1.237966
C	-2.762196	-1.640051	-2.962323
H	-3.092836	-2.667048	-3.089502
H	-3.373546	-1.020869	-3.613986
H	-1.728661	-1.566320	-3.282103
C	-4.513667	-0.584207	1.723892
H	-5.519195	-0.943819	1.542954
H	-4.095660	-1.139450	2.558028
H	-4.570633	0.460335	2.014903
C	0.519390	3.471641	-1.763831
H	-0.044234	4.305635	-2.171429
H	1.525305	3.829891	-1.559853
H	0.577197	2.694001	-2.514018
C	-1.708009	3.059967	2.809507
H	-1.659305	4.118092	3.035925
H	-2.749144	2.752325	2.837051
H	-1.181878	2.520000	3.590989
C	2.460140	-2.571717	0.227136
H	3.433124	-2.108603	0.360622
H	2.553533	-3.621954	0.487557
H	2.192972	-2.490735	-0.818733
C	-0.404924	-0.996259	4.147961
H	0.155490	-1.536501	4.901152
H	-0.387778	0.059225	4.402698
H	-1.437680	-1.329921	4.185109
H	4.979417	-1.902924	-2.808834
C	5.090497	0.275180	-1.234817
F	6.212906	0.211188	-1.956548
F	4.962645	1.534184	-0.813926
F	5.308764	-0.473845	-0.140785

S-I6

Cu	0.261877	-0.358978	-1.043191
B	-1.235338	0.471336	1.391602
H	-1.832872	0.792998	2.363877
C	-3.566377	-0.253821	0.511363
C	-4.019000	-0.846672	-0.646857
C	-2.893875	-1.080228	-1.444753

N	-1.824112	-0.651423	-0.800875
N	-2.231688	-0.149087	0.387587
N	0.692662	-1.081352	0.887204
C	1.512866	-1.896001	1.523582
C	1.181272	-1.892150	2.882205
C	0.116267	-1.028800	3.026022
N	-0.155010	-0.553675	1.798645
N	0.105645	1.680487	-0.382415
C	0.522589	2.903888	-0.656597
C	0.098124	3.761447	0.363258
C	-0.599741	2.986153	1.264536
N	-0.578017	1.732202	0.782747
O	0.114112	-3.148420	-3.850077
C	1.139525	-2.423204	-3.372208
C	2.365634	-2.976586	-3.051172
C	3.392575	-2.164332	-2.588069
C	3.199744	-0.807183	-2.442749
C	1.969123	-0.241278	-2.785484
C	0.933003	-1.041734	-3.242736
H	0.333226	-4.077150	-3.868558
H	2.529629	-4.035756	-3.173508
H	1.853940	0.826546	-2.773270
H	0.017436	-0.615406	-3.616049
Br	0.412278	5.609847	0.481005
Br	2.025515	-2.874307	4.242844
Br	-5.797900	-1.265808	-1.080413
C	-2.841138	-1.684229	-2.802609
H	-3.612932	-2.439649	-2.909375
H	-3.018235	-0.933406	-3.570745
H	-1.880624	-2.147987	-2.988189
C	-4.341531	0.200074	1.697594
H	-5.392912	-0.010879	1.543047
H	-4.020385	-0.308602	2.601570
H	-4.229072	1.267706	1.861550
C	1.308794	3.244324	-1.874801
H	1.350049	4.319506	-2.004684
H	2.331350	2.882125	-1.806074
H	0.848987	2.821020	-2.763674
C	-1.267232	3.392483	2.530889
H	-1.128783	4.455752	2.685682
H	-2.333256	3.187649	2.503815
H	-0.851553	2.867493	3.385780
C	2.597662	-2.643233	0.834703
H	3.469081	-2.010196	0.687785
H	2.900283	-3.502251	1.423204
H	2.262637	-2.986490	-0.137540
C	-0.631098	-0.657384	4.257847
H	-0.218957	-1.193036	5.104595
H	-0.560349	0.406985	4.461232
H	-1.684284	-0.908554	4.174852
H	4.346446	-2.599336	-2.344050
C	4.276857	0.059057	-1.880666
F	5.485710	-0.471958	-2.056655
F	4.280640	1.270804	-2.443229
F	4.122939	0.249792	-0.566389

-NO2

$C_6H_5(NO_2)$

C	-1.377197	1.425719	0.000706
C	-0.001831	1.500756	-0.165497
C	0.631419	2.730697	-0.167684
C	-0.138126	3.868084	-0.001366

C	-1.510308	3.817540	0.165936
C	-2.128866	2.580096	0.165840
H	-1.864715	0.464668	0.001512
H	0.578026	0.602244	-0.293428
H	1.694341	2.823604	-0.293906
H	-2.063110	4.730263	0.291318
H	-3.196310	2.517568	0.294608
N	0.521356	5.168033	-0.002515
O	-0.171458	6.149148	0.144267
O	1.722175	5.188249	-0.150195

T-I1

Cu	-0.339035	-0.084966	-1.274447
B	-2.120551	0.061264	1.175712
H	-2.843358	0.111415	2.114176
C	-0.798122	-1.796296	2.464026
C	0.297093	-2.574119	2.148524
C	0.674986	-2.230949	0.846168
N	-0.154939	-1.303344	0.408938
N	-1.050510	-1.034184	1.386733
N	-0.602723	1.609013	-0.091765
C	-0.105614	2.829287	-0.028658
C	-0.642880	3.459698	1.098502
C	-1.480626	2.545987	1.705189
N	-1.436144	1.430251	0.958400
N	-2.298204	-0.390692	-1.283191
C	-3.186708	-0.693699	-2.215018
C	-4.435988	-0.787325	-1.601212
C	-4.241621	-0.524486	-0.257642
N	-2.932920	-0.287251	-0.096846
O	1.232616	0.008305	-2.168238
C	4.427153	-0.170205	-2.775751
C	5.610421	-0.435788	-3.450517
C	6.818621	-0.402832	-2.777108
C	6.812273	-0.100701	-1.426593
C	5.645041	0.168561	-0.732788
C	4.445572	0.131413	-1.420794
H	3.478459	-0.194093	-3.284789
H	5.594274	-0.669200	-4.502132
H	7.751750	-0.603742	-3.270427
H	3.514670	0.332918	-0.919655
C	1.789907	-2.759963	0.017522
H	1.591404	-3.783105	-0.292027
H	1.929513	-2.146442	-0.865888
H	2.716686	-2.765645	0.583115
C	-1.591269	-1.748492	3.721348
H	-1.206758	-2.481461	4.419967
H	-1.533227	-0.770001	4.189342
H	-2.638854	-1.966988	3.537686
C	-2.821791	-0.882608	-3.643477
H	-3.082223	-1.882921	-3.977614
H	-3.357506	-0.178106	-4.273319
H	-1.756951	-0.736729	-3.793676
C	-5.231296	-0.492459	0.851613
H	-6.216974	-0.720611	0.465224
H	-4.986334	-1.221198	1.618273
H	-5.265182	0.485980	1.321128
C	0.853860	3.351220	-1.036944
H	1.243619	2.539078	-1.641513
H	0.375322	4.077417	-1.689502
H	1.685058	3.850605	-0.548803
C	-2.297499	2.688157	2.940010
H	-3.349875	2.509322	2.741530
H	-1.980614	1.987486	3.707159
H	-2.189281	3.691949	3.332280
Br	-0.283703	5.211820	1.659433

Br	1.130902	-3.850686	3.238514
Br	-6.052834	-1.192843	-2.455098
H	5.694001	0.398896	0.315682
N	8.079435	-0.065003	-0.710253
O	8.052283	0.199059	0.471126
O	9.088519	-0.301768	-1.336393

T-TS1

Cu	0.434074	0.388258	-0.861928
B	-1.493213	-0.590691	1.316094
H	-2.198274	-0.952761	2.198064
C	-2.982544	-2.028192	-0.288789
C	-2.942492	-2.275448	-1.644324
C	-1.833774	-1.583552	-2.149131
N	-1.244705	-0.961836	-1.148155
N	-1.936917	-1.226839	-0.017156
N	0.939453	-0.711595	0.722248
C	2.061394	-1.291607	1.121666
C	1.795326	-1.968298	2.312309
C	0.456448	-1.773649	2.592171
N	-0.035526	-1.008243	1.609058
N	-0.834513	1.593702	0.249659
C	-1.219122	2.858815	0.228287
C	-2.202953	3.034976	1.203654
C	-2.400691	1.804211	1.795306
N	-1.558038	0.950214	1.194928
O	0.881909	0.958117	-2.534935
C	3.083364	1.635702	-3.643980
C	4.273093	1.136293	-3.190135
C	4.624670	1.338862	-1.852918
C	3.818284	2.055042	-0.978187
C	2.626282	2.566893	-1.441650
C	2.180255	2.301335	-2.764186
H	2.793296	1.492529	-4.670356
H	4.150206	2.222245	0.030031
H	2.021973	3.174303	-0.791393
H	1.442156	2.951201	-3.196412
C	-3.951038	-2.509163	0.732691
H	-4.474788	-1.682413	1.204014
H	-4.685641	-3.151053	0.261728
H	-3.454590	-3.075752	1.515054
C	-1.325810	-1.500337	-3.543974
H	-0.976499	-2.471308	-3.886494
H	-2.115352	-1.183313	-4.219612
H	-0.508058	-0.789656	-3.601168
C	-0.674823	3.858334	-0.727563
H	-1.470169	4.507849	-1.078469
H	0.074487	4.492055	-0.258197
H	-0.237214	3.355084	-1.581156
C	-3.345631	1.421280	2.878324
H	-3.844019	2.307070	3.252838
H	-4.103810	0.732165	2.517568
H	-2.827715	0.942839	3.703411
C	3.334213	-1.209551	0.360452
H	3.154107	-0.851584	-0.647067
H	4.048640	-0.544905	0.838333
H	3.795017	-2.190526	0.299183
C	-0.352851	-2.284876	3.730190
H	-0.839539	-1.474265	4.263181
H	-1.124070	-2.969696	3.389705
H	0.290326	-2.814307	4.422344
Br	3.029679	-2.953552	3.318847
Br	-4.132271	-3.331549	-2.638444
Br	-3.080467	4.640311	1.612032
H	4.947877	0.604657	-3.835503
N	5.875409	0.800135	-1.365911

O	6.102934	0.886278	-0.175685
O	6.622774	0.288101	-2.169809

T-I2

Cu	0.313146	0.225878	-0.932894
B	-1.791394	-0.120465	1.253291
H	-2.581043	-0.199870	2.134320
C	-3.165584	-1.973616	0.016680
C	-3.021433	-2.591992	-1.206814
C	-1.889978	-2.030746	-1.813313
N	-1.388644	-1.126939	-0.995826
N	-2.158233	-1.087892	0.114921
N	0.701185	-0.372083	0.970060
C	1.767988	-0.753659	1.652484
C	1.360511	-1.085232	2.946095
C	-0.004430	-0.885388	2.998189
N	-0.376992	-0.451934	1.785003
N	-0.909998	1.657770	-0.286645
C	-1.121619	2.918962	-0.626696
C	-2.150498	3.413641	0.174578
C	-2.542746	2.379142	1.003742
N	-1.770892	1.327592	0.699374
O	1.294155	-0.085543	-2.434144
C	3.468977	-0.935106	-2.906458
C	4.746586	-0.899459	-2.463880
C	5.255343	0.298199	-1.914171
C	4.453106	1.452869	-1.765094
C	3.175160	1.433521	-2.205718
C	2.556052	0.256127	-2.899675
H	3.050342	-1.844576	-3.305021
H	4.881357	2.318255	-1.293049
H	2.554180	2.308894	-2.097773
H	2.444362	0.554384	-3.956660
C	-4.194572	-2.184838	1.069811
H	-4.748604	-1.272846	1.272499
H	-4.896010	-2.943470	0.744432
H	-3.746119	-2.514116	2.002759
C	-1.281063	-2.327250	-3.136909
H	-1.023285	-3.380421	-3.212235
H	-1.980985	-2.108226	-3.939384
H	-0.385445	-1.730989	-3.272808
C	-0.347410	3.608214	-1.692072
H	-1.011032	4.179557	-2.333217
H	0.371024	4.303121	-1.263832
H	0.184787	2.888500	-2.304573
C	-3.603815	2.355582	2.045381
H	-4.058895	3.335458	2.121447
H	-4.379874	1.636459	1.800674
H	-3.197346	2.088945	3.015964
C	3.132860	-0.791234	1.068471
H	3.090623	-0.720839	-0.011036
H	3.739432	0.028105	1.446246
H	3.632635	-1.716879	1.336142
C	-0.943782	-1.093348	4.132468
H	-1.470298	-0.177602	4.383157
H	-1.686464	-1.849743	3.896509
H	-0.390687	-1.419842	5.004588
Br	2.470903	-1.682847	4.331776
Br	-4.118034	-3.926950	-1.937178
Br	-2.851057	5.150154	0.121538
H	5.386153	-1.762444	-2.498224
N	6.609431	0.330411	-1.447988
O	7.014594	1.364663	-0.951661
O	7.278074	-0.677277	-1.574578

MECP1

Cu	0.18580757	0.18660623	-0.99046661
B	-1.85611352	-0.11994285	1.21466007
H	-2.66055981	-0.20368482	2.08196290
C	-3.21834691	-1.99045246	-0.00515282
C	-3.08021538	-2.62238860	-1.22274233
C	-1.95828027	-2.06099427	-1.84365400
N	-1.45581122	-1.14478422	-1.04094678
N	-2.21681792	-1.09625478	0.07509658
N	0.64156110	-0.39919984	0.98800401
C	1.68466724	-0.76054942	1.71242245
C	1.24560200	-1.05137221	3.00774639
C	-0.11804135	-0.84875150	3.01853993
N	-0.45508242	-0.45317218	1.77923899
N	-1.00828189	1.67148947	-0.33593333
C	-1.20876775	2.94399510	-0.62985450
C	-2.20491911	3.43576861	0.21643921
C	-2.58996002	2.38616333	1.02739657
N	-1.84618729	1.32982425	0.66594681
O	1.42407937	-0.19496820	-2.40462547
C	3.62572958	-1.03448884	-2.79473569
C	4.95905574	-0.89033323	-2.61325451
C	5.48954369	0.32347494	-2.11241736
C	4.67620841	1.39427257	-1.79202776
C	3.30114307	1.26458241	-1.95626822
C	2.69091649	0.04128044	-2.46886543
H	3.19752490	-1.95300119	-3.15696044
H	5.10778679	2.30929074	-1.43196955
H	2.62890201	2.06782519	-1.70189069
H	2.98545473	0.94207964	-3.33119746
C	-4.23643721	-2.20473903	1.05787476
H	-4.79049466	-1.29454315	1.26698839
H	-4.93860346	-2.96503359	0.73767216
H	-3.77821071	-2.53464110	1.98599877
C	-1.36170018	-2.36714984	-3.17055781
H	-1.13499412	-3.42643510	-3.25510303
H	-2.05338635	-2.12013270	-3.97231406
H	-0.44746774	-1.80020968	-3.30645324
C	-0.45546132	3.64784234	-1.70035184
H	-1.13327486	4.16637609	-2.37185517
H	0.21749375	4.39265449	-1.28221125
H	0.12400264	2.93778082	-2.28190364
C	-3.61249319	2.35406105	2.10679690
H	-4.06811276	3.33191687	2.20524778
H	-4.39419755	1.63283775	1.88879772
H	-3.17008246	2.08507065	3.06130571
C	3.06662096	-0.81925665	1.16880646
H	3.04811640	-0.82171656	0.08578499
H	3.65884961	0.02915228	1.50382280
H	3.57118659	-1.71969034	1.50537617
C	-1.08440254	-1.02100018	4.13637132
H	-1.60622917	-0.09452998	4.35654970
H	-1.83050763	-1.77503083	3.90337498
H	-0.55341912	-1.33338776	5.02760015
Br	2.32006943	-1.60946870	4.44092056
Br	-4.17381302	-3.97277905	-1.93082121
Br	-2.87641400	5.18650383	0.23079453
H	5.64414649	-1.68939346	-2.83286647
N	6.90269952	0.44262108	-1.93371572
O	7.33174365	1.49847542	-1.50505319
O	7.59288001	-0.51973085	-2.21829494

S-I3

Cu	0.131777	0.000838	1.187017
B	-1.791275	-0.002103	-1.114382
H	-2.568145	-0.003145	-2.011801

C	-3.905072	-0.004938	0.414921
C	-4.101277	-0.003799	1.778826
C	-2.831173	-0.000392	2.366808
N	-1.927112	0.000448	1.408975
N	-2.573184	-0.002287	0.221692
N	0.039419	1.532559	-0.301506
C	0.589300	2.690133	-0.609426
C	-0.023560	3.183578	-1.766368
C	-0.978517	2.258529	-2.130076
N	-0.914354	1.269160	-1.220367
N	0.039376	-1.534819	-0.297992
C	0.593257	-2.690264	-0.606675
C	-0.014526	-3.182690	-1.766741
C	-0.970602	-2.259244	-2.131566
N	-0.911971	-1.271763	-1.219416
O	1.760158	0.001964	2.477006
C	3.956009	0.004264	3.327667
C	5.387093	0.004863	2.934625
C	5.728225	0.003525	1.649476
C	4.756008	0.001464	0.575164
C	3.440899	0.000943	0.831504
C	2.949652	0.002353	2.200886
H	3.745941	0.867310	3.961084
H	5.129473	0.000368	-0.433349
H	2.705541	-0.000555	0.044448
H	3.747110	-0.857762	3.962865
C	-4.912697	-0.008663	-0.679499
H	-4.811103	-0.886584	-1.310705
H	-5.908679	-0.009709	-0.253034
H	-4.814276	0.867272	-1.313934
C	-2.464252	0.001978	3.807196
H	-2.865680	0.877629	4.310708
H	-2.862238	-0.874097	4.312695
H	-1.384753	0.004220	3.913142
C	1.676674	-3.298797	0.210612
H	1.440041	-4.329312	0.459765
H	2.623365	-3.305142	-0.325055
H	1.799574	-2.741679	1.132916
C	-1.912421	-2.286115	-3.283015
H	-1.738529	-3.179320	-3.871123
H	-2.946444	-2.292648	-2.950942
H	-1.778433	-1.421879	-3.926708
C	1.674125	3.299507	0.205361
H	1.799993	2.742187	1.127138
H	2.619455	3.307043	-0.332693
H	1.437001	4.329664	0.455480
C	-1.924250	2.285655	-3.278313
H	-1.791306	1.422480	-3.923623
H	-2.957162	2.290373	-2.942738
H	-1.753584	3.179924	-3.865749
Br	0.386708	4.796829	-2.636217
Br	-5.742812	-0.006210	2.691179
Br	0.402459	-4.793162	-2.638544
H	6.148358	0.006437	3.695231
N	7.142914	0.004258	1.289504
O	7.406901	0.003044	0.109905
O	7.952178	0.005982	2.186260

$C_6H_5(NO_2)O$

O	1.754491	0.001676	2.458165
C	3.956435	0.004343	3.334442
C	5.387012	0.004911	2.934716
C	5.726330	0.003593	1.649303
C	4.754818	0.001531	0.569843
C	3.444978	0.000938	0.831864
C	2.934321	0.002547	2.208556

H	3.751447	0.867694	3.968362
H	5.131221	0.000482	-0.437113
H	2.708692	-0.000641	0.046440
H	3.752655	-0.858045	3.970074
H	6.152943	0.006354	3.690784
N	7.140201	0.004156	1.290800
O	7.407447	0.002977	0.110781
O	7.953469	0.005942	2.185261

$C_6H_4(NO_2)OH$

C	-1.473853	1.471915	-0.119566
C	-0.093352	1.512793	-0.291003
C	0.571992	2.720447	-0.234245
C	-0.151379	3.877160	-0.006236
C	-1.527305	3.850706	0.166310
C	-2.189208	2.644957	0.109370
H	0.457085	0.602115	-0.468093
H	1.637127	2.779428	-0.363296
H	-2.054494	4.770540	0.341481
H	-3.255761	2.585676	0.238520
N	0.546015	5.145057	0.053443
O	-0.112698	6.141635	0.254142
O	1.747894	5.135534	-0.100734
O	-2.173137	0.325696	-0.164814
H	-1.594175	-0.415342	-0.326120

T-TS2

Cu	0.225798	0.118525	-0.883326
B	-2.107416	0.017508	1.086949
H	-2.979943	0.013253	1.890046
C	-3.818774	-1.016268	-0.605432
C	-3.724183	-1.386652	-1.930106
C	-2.418519	-1.089214	-2.341859
N	-1.773292	-0.567658	-1.318246
N	-2.616363	-0.521158	-0.262538
N	0.205204	-0.958167	0.852284
C	1.014939	-1.809917	1.457172
C	0.381878	-2.262486	2.617043
C	-0.849640	-1.640969	2.667987
N	-0.928940	-0.854473	1.584103
N	-0.559461	1.691691	0.042240
C	-0.328065	2.993334	-0.001704
C	-1.238103	3.619943	0.849757
C	-2.024444	2.622658	1.396617
N	-1.586744	1.463746	0.886684
O	1.275452	-0.277946	-2.317374
C	3.684475	-0.042907	-1.899503
C	4.205983	1.036742	-1.216948
C	5.573898	1.084686	-0.996718
C	6.357966	0.047817	-1.472952
C	5.831255	-1.032921	-2.160431
C	4.463185	-1.077329	-2.377063
H	2.372094	-0.126002	-2.176311
H	3.573548	1.832380	-0.860530
H	6.036211	1.900569	-0.471560
H	4.018274	-1.901571	-2.908469
C	-1.775884	-1.284889	-3.668165
H	-2.256390	-0.668439	-4.423816
H	-0.723814	-1.025030	-3.616345
H	-1.867590	-2.319200	-3.987990
C	-4.975376	-1.112861	0.324842
H	-5.830734	-1.519233	-0.201111
H	-4.754581	-1.762666	1.166973

H	-5.248993	-0.139241	0.720652
C	0.739193	3.592705	-0.845200
H	0.335807	4.392304	-1.459117
H	1.525032	4.021969	-0.228581
H	1.175581	2.846819	-1.501610
C	-3.152507	2.730647	2.359479
H	-3.290762	3.767122	2.641689
H	-4.078518	2.368894	1.922528
H	-2.960397	2.152291	3.257824
C	2.354734	-2.167944	0.924505
H	2.458217	-1.835509	-0.100985
H	3.143614	-1.713166	1.518579
H	2.499542	-3.243294	0.959463
C	-1.928965	-1.770204	3.683177
H	-2.174426	-0.808983	4.124082
H	-2.836251	-2.176480	3.245837
H	-1.604454	-2.436455	4.473059
Br	1.087621	-3.479292	3.855369
Br	-5.072094	-2.142198	-2.993114
Br	-1.353287	5.461027	1.174703
H	6.486955	-1.809358	-2.510288
N	7.795690	0.096626	-1.241684
O	8.463905	-0.819357	-1.664135
O	8.235839	1.050099	-0.640080

T-I4

Cu	-0.579590	-0.033910	-1.581701
B	-1.740773	0.062819	1.237206
H	-2.231567	0.118421	2.315372
C	-0.428632	-2.039738	2.080173
C	0.453178	-2.908426	1.472785
C	0.576749	-2.495022	0.140502
N	-0.192681	-1.439214	-0.034829
N	-0.805400	-1.157872	1.137775
N	-0.351266	1.546237	-0.247968
C	0.315901	2.684835	-0.209545
C	0.139299	3.252129	1.055474
C	-0.663445	2.386867	1.770944
N	-0.945842	1.360603	0.952853
N	-2.511623	-0.131562	-1.142173
C	-3.614613	-0.260098	-1.860706
C	-4.698334	-0.280438	-0.982734
C	-4.180206	-0.159265	0.293523
N	-2.849853	-0.070423	0.162631
O	0.747948	-0.192612	-2.809552
C	3.708407	0.491204	-0.108762
C	4.839461	0.712254	0.635440
C	6.052872	0.404193	0.034906
C	6.044214	-0.100077	-1.256433
C	4.877818	-0.311248	-1.975711
C	3.655924	-0.006134	-1.386557
H	0.857657	0.546838	-3.399974
H	4.802977	1.104986	1.637540
H	6.989392	0.545797	0.543036
H	2.723335	-0.153275	-1.913972
C	1.395386	-3.073931	-0.957067
H	1.074323	-4.087956	-1.181760
H	1.308303	-2.465150	-1.850151
H	2.441538	-3.120718	-0.667873
C	-0.914169	-2.014126	3.485900
H	-0.483236	-2.843753	4.033021
H	-0.630500	-1.093014	3.987066
H	-1.995861	-2.097826	3.533972
C	-3.601319	-0.358677	-3.343834
H	-4.096625	-1.267875	-3.671865
H	-4.127916	0.479897	-3.791211

H	-2.583888	-0.368903	-3.721224
C	-4.888238	-0.127473	1.600732
H	-5.955603	-0.211605	1.437287
H	-4.573553	-0.947682	2.238818
H	-4.694581	0.799855	2.131231
C	1.094145	3.199273	-1.366742
H	1.241442	2.412667	-2.097064
H	0.583039	4.033203	-1.841794
H	2.067892	3.552675	-1.043034
C	-1.155169	2.496187	3.170518
H	-2.239585	2.465580	3.212668
H	-0.776345	1.686801	3.787841
H	-0.822323	3.433747	3.598823
Br	0.861555	4.877670	1.647655
Br	1.326317	-4.369093	2.260310
Br	-6.501108	-0.441779	-1.464567
H	4.936318	-0.706379	-2.973857
N	7.318467	-0.420669	-1.884005
O	7.296793	-0.858211	-3.012372
O	8.326839	-0.230226	-1.241242

T-TS3

Cu	-0.373147	0.149817	-1.011263
B	1.492466	-0.311026	1.330768
H	2.187833	-0.533126	2.264170
C	2.987207	-2.009472	0.014439
C	2.943544	-2.526628	-1.264209
C	1.830921	-1.959817	-1.893004
N	1.244108	-1.146745	-1.032739
N	1.941391	-1.174991	0.127168
N	0.842147	1.677695	-0.062879
C	1.163420	2.952046	-0.211548
C	2.119516	3.283471	0.753411
C	2.360964	2.139155	1.483148
N	1.572941	1.183399	0.964193
N	-0.925320	-0.630577	0.715884
C	-2.038340	-1.163853	1.200999
C	-1.787994	-1.549626	2.518198
C	-0.468803	-1.238899	2.784710
N	0.031214	-0.685082	1.671994
O	-0.497464	0.650719	-2.822454
C	-2.069722	1.562118	-2.852649
C	-2.464505	1.883821	-1.538415
C	-3.751990	1.602937	-1.118725
C	-4.629909	0.964525	-1.980358
C	-4.240274	0.616198	-3.280371
C	-2.963941	0.885200	-3.701893
H	0.192074	1.225015	-3.150313
H	-1.823887	2.462091	-0.891157
H	-4.956509	0.141174	-3.927178
H	-2.654815	0.609132	-4.696526
Br	-3.017476	-2.349642	3.680963
Br	2.909133	4.966650	0.992386
Br	4.131048	-3.759009	-2.028020
C	1.318007	-2.186620	-3.270038
H	2.117671	-2.072391	-3.996243
H	0.931789	-3.197821	-3.372253
H	0.528513	-1.481003	-3.500111
C	3.964671	-2.271045	1.104546
H	4.702649	-2.987272	0.764714
H	4.481888	-1.362512	1.398770
H	3.475868	-2.675167	1.985842
C	-3.284960	-1.335433	0.411226
H	-4.067261	-0.655342	0.736291
H	-3.107070	-1.162332	-0.643491
H	-3.660374	-2.346499	0.536846

C	0.315528	-1.452466	4.029890
H	-0.334985	-1.839696	4.804292
H	1.120365	-2.164559	3.872547
H	0.755467	-0.524817	4.381931
C	0.577362	3.837472	-1.253943
H	-0.086376	4.575227	-0.809675
H	1.363640	4.382474	-1.767777
H	0.012807	3.270721	-1.983732
C	3.292498	1.927698	2.623454
H	3.773123	2.865034	2.875585
H	2.767530	1.564828	3.501688
H	4.065231	1.205427	2.376641
H	-4.093501	1.888849	-0.139526
N	-5.945003	0.636709	-1.522035
O	-6.203912	0.821635	-0.344613
O	-6.735746	0.178331	-2.322857

T-I5

Cu	-0.469231	0.123652	-1.004713
B	1.431626	-0.264082	1.340113
H	2.130632	-0.479277	2.272261
C	2.430104	-2.448191	0.309721
C	2.178555	-3.164630	-0.843037
C	1.175754	-2.481509	-1.534380
N	0.852365	-1.407777	-0.831026
N	1.608747	-1.388246	0.290769
N	1.173948	1.534584	-0.392662
C	1.721805	2.689847	-0.729824
C	2.731133	2.993481	0.189092
C	2.763074	1.957156	1.096660
N	1.809496	1.090575	0.715495
N	-1.048807	-0.259608	0.883261
C	-2.193386	-0.465775	1.518984
C	-1.912430	-0.581932	2.882402
C	-0.545814	-0.456844	3.024398
N	-0.045456	-0.266273	1.794689
O	-0.276996	0.346888	-3.075057
C	-1.575095	0.959090	-3.124507
C	-1.920413	1.581418	-1.853648
C	-3.275170	1.642472	-1.508331
C	-4.230388	1.024159	-2.274923
C	-3.872816	0.326401	-3.478885
C	-2.561584	0.264521	-3.849614
H	0.408700	0.954196	-3.348546
H	-1.297053	2.374841	-1.461600
H	-4.650039	-0.135047	-4.058306
H	-2.266494	-0.303019	-4.717994
Br	-3.164580	-0.875308	4.243971
Br	3.821122	4.518692	0.168345
Br	3.002727	-4.756196	-1.389031
C	0.520700	-2.855075	-2.815683
H	1.267171	-3.122616	-3.557494
H	-0.120391	-3.721893	-2.674974
H	-0.073161	-2.036215	-3.199918
C	3.401385	-2.729469	1.400282
H	3.950863	-3.634141	1.170737
H	4.112662	-1.917194	1.515188
H	2.897273	-2.867493	2.351955
C	-3.509413	-0.602871	0.842139
H	-4.113058	0.296848	0.922482
H	-3.383113	-0.827584	-0.209335
H	-4.070276	-1.409667	1.303136
C	0.280654	-0.518761	4.259332
H	-0.367688	-0.598638	5.123158
H	0.943977	-1.378719	4.249868
H	0.891450	0.371699	4.370619

C	1.307175	3.496207	-1.910574
H	0.936865	4.470604	-1.603384
H	2.152881	3.669521	-2.570592
H	0.521568	3.007442	-2.473640
C	3.645424	1.767507	2.279285
H	4.303830	2.621817	2.378952
H	3.068256	1.672918	3.194023
H	4.257208	0.875674	2.180088
H	-3.591714	2.194596	-0.640883
N	-5.576855	1.052882	-1.851810
O	-5.842659	1.558620	-0.764825
O	-6.418441	0.560500	-2.588746

MECP2

Cu	-0.48155347	0.12092151	-1.01169338
B	1.43302811	-0.26011864	1.34056337
H	2.13105025	-0.47929167	2.27222089
C	2.42530061	-2.44294644	0.30669465
C	2.17225030	-3.15832109	-0.84636559
C	1.16811511	-2.47578638	-1.53556893
N	0.84447392	-1.40223368	-0.82976453
N	1.60385663	-1.38387619	0.29044654
N	1.17674780	1.53223766	-0.39285610
C	1.72194846	2.68860587	-0.73085650
C	2.73110311	2.99476204	0.18802067
C	2.76523122	1.95956433	1.09648630
N	1.81321239	1.09120895	0.71577118
N	-1.04542766	-0.25501561	0.87875159
C	-2.19168553	-0.46376727	1.51218730
C	-1.91309234	-0.58016573	2.87552674
C	-0.54685909	-0.45340755	3.02021671
N	-0.04430958	-0.26080729	1.79242387
O	-0.28370926	0.31231639	-3.03956775
C	-1.55927764	0.99876949	-3.14025694
C	-1.91116376	1.57963982	-1.83913980
C	-3.27110715	1.62882718	-1.49517473
C	-4.22456341	1.01739675	-2.26332180
C	-3.85797065	0.32027999	-3.47078712
C	-2.55664009	0.28015894	-3.85379938
H	0.43506010	0.87954419	-3.31310678
H	-1.31972358	2.40652249	-1.46363781
H	-4.63317231	-0.15355484	-4.04345361
H	-2.26502904	-0.27425061	-4.73236896
Br	-3.16788635	-0.87575983	4.23349396
Br	3.81905703	4.52125980	0.16521519
Br	2.99526387	-4.74938112	-1.39409460
C	0.51548815	-2.85328795	-2.81680371
H	1.26515141	-3.12166308	-3.55507194
H	-0.12280034	-3.72180505	-2.67425237
H	-0.07930376	-2.03778186	-3.20566900
C	3.39771037	-2.72621947	1.39600840
H	3.94586356	-3.63116605	1.16461714
H	4.10973855	-1.91477937	1.51149078
H	2.89400913	-2.86522431	2.34770777
C	-3.50785303	-0.60307445	0.83672031
H	-4.11164269	0.29633770	0.91804376
H	-3.38382515	-0.82814640	-0.21469522
H	-4.06706528	-1.40964589	1.30000188
C	0.27819572	-0.51718552	4.25593457
H	-0.37082047	-0.59754753	5.11906423
H	0.94059251	-1.37785466	4.24559342
H	0.88968762	0.37255623	4.36831132
C	1.30454748	3.49345411	-1.91144480
H	0.93450505	4.46759847	-1.60298245
H	2.14967861	3.66792897	-2.57191033
H	0.51899303	3.00349321	-2.47344634

C	3.64749787	1.77018448	2.27936898
H	4.30618859	2.62412669	2.37961585
H	3.07000597	1.67548851	3.19393049
H	4.25904794	0.87809706	2.18027341
H	-3.58833639	2.17317720	-0.62305730
N	-5.57337037	1.04892741	-1.84923854
O	-5.84626711	1.55469650	-0.76496921
O	-6.41153585	0.56074154	-2.59188269

S-I6

Cu	-0.144751	-0.000106	1.105614
B	1.817633	-0.000245	-1.089829
H	2.619245	-0.000283	-1.964344
C	1.036584	2.260960	-2.126478
C	0.065852	3.182292	-1.797643
C	-0.587022	2.688173	-0.663107
N	-0.042046	1.534086	-0.333393
N	0.945886	1.272129	-1.218348
N	1.896405	-0.001662	1.441521
C	2.781311	-0.001707	2.417772
C	4.062974	-0.000751	1.858124
C	3.895212	-0.000097	0.489984
N	2.568279	-0.000689	0.268016
N	-0.044071	-1.532998	-0.335080
C	-0.589392	-2.686839	-0.665066
C	0.064614	-3.181840	-1.798567
C	1.036401	-2.261307	-2.126493
N	0.945191	-1.272056	-1.218863
O	-1.811323	0.000392	2.507572
C	-3.146640	0.000321	2.240215
C	-4.083815	-0.001910	3.263229
C	-5.429305	-0.001822	2.951985
C	-5.808692	0.000420	1.622498
C	-4.877659	0.002598	0.596942
C	-3.533557	0.002548	0.907067
H	-1.642181	-0.001610	3.446773
H	-3.767484	-0.003622	4.294444
H	-5.214063	0.004274	-0.423648
H	-2.781880	0.004151	0.135968
Br	-0.321427	-4.791951	-2.683456
Br	5.683107	-0.000490	2.806521
Br	-0.320146	4.792376	-2.682599
C	-1.704925	3.295788	0.106267
H	-1.435360	4.285878	0.464116
H	-2.592904	3.406339	-0.510989
H	-1.952000	2.675040	0.960126
C	2.019403	2.289962	-3.243047
H	1.866585	3.184387	-3.835014
H	3.040879	2.295961	-2.874133
H	1.908306	1.427204	-3.893009
C	-1.708792	-3.293366	0.102981
H	-1.440202	-4.283227	0.462204
H	-1.957138	-2.671728	0.955817
H	-2.595740	-3.404156	-0.515707
C	2.020688	-2.291475	-3.241737
H	1.867696	-3.185793	-3.833823
H	1.911451	-1.428656	-3.891927
H	3.041658	-2.298590	-2.871431
C	2.390170	-0.002687	3.851689
H	2.779379	-0.878688	4.363737
H	2.777898	0.873500	4.364546
H	1.308638	-0.003652	3.939515
C	4.927036	0.001120	-0.581605
H	5.913118	0.000967	-0.132821
H	4.841319	-0.875239	-1.217104
H	4.840886	0.878634	-1.215466

H	-6.181641	-0.003468	3.719035
N	-7.223784	0.000486	1.294773
O	-8.011529	-0.001478	2.213985
O	-7.527673	0.002533	0.123780

-Cl

C_6H_5Cl

C	-1.686059	1.296171	0.000068
C	-0.300001	1.293289	0.000589
C	0.390626	2.494920	-0.000045
C	-0.295682	3.699044	-0.001110
C	-1.679789	3.683485	-0.001586
C	-2.385509	2.492590	-0.001038
H	-2.229492	0.365737	0.000515
H	0.238520	0.360283	0.001381
H	1.468123	2.500320	0.000290
H	0.232068	4.636893	-0.001587
H	-3.461584	2.504740	-0.001418
Cl	-2.551109	5.192686	-0.002879

T-I1

Cu	-0.277591	0.095350	-1.344580
B	-1.891795	-0.068857	1.220383
H	-2.550000	-0.125498	2.205057
C	-1.308858	-2.590966	1.626523
C	-0.545952	-3.510904	0.936287
C	-0.063454	-2.861433	-0.204938
N	-0.521377	-1.624441	-0.196240
N	-1.275618	-1.453257	0.913177
N	0.066461	1.249971	0.361710
C	0.960669	2.129989	0.769184
C	0.686617	2.441059	2.105543
C	-0.414105	1.694160	2.471766
N	-0.769676	0.979922	1.390691
N	-2.220444	0.470961	-1.205006
C	-3.158418	0.835832	-2.063012
C	-4.358477	0.953278	-1.361261
C	-4.082814	0.639190	-0.043199
N	-2.775939	0.350763	0.018865
O	1.220042	-0.025589	-2.350319
C	4.465984	0.251332	-2.880016
C	5.662037	0.627300	-3.470713
C	6.849248	0.565206	-2.757280
C	6.820963	0.121770	-1.446696
C	5.636380	-0.258333	-0.839459
C	4.457688	-0.190585	-1.565683
H	3.536333	0.294973	-3.421321
H	5.677997	0.971305	-4.492273
H	7.783264	0.854795	-3.207087
H	3.521753	-0.478144	-1.117232
C	0.808625	-3.379768	-1.291545
H	0.259881	-4.058100	-1.940436
H	1.195712	-2.559898	-1.887395
H	1.645619	-3.932576	-0.876420
C	-2.045582	-2.746986	2.909216
H	-1.951711	-3.767792	3.259031
H	-1.649587	-2.087318	3.676034
H	-3.100839	-2.521103	2.789899
C	-2.884834	1.058843	-3.506918
H	-3.492031	0.398361	-4.119371
H	-3.124863	2.079693	-3.790508
H	-1.840011	0.875618	-3.735967
C	-4.994905	0.604767	1.130662

H	-5.995292	0.881181	0.821124
H	-5.033859	-0.387410	1.569995
H	-4.670661	1.297441	1.901321
C	2.036950	2.644610	-0.117401
H	2.080236	2.069150	-1.035429
H	1.869247	3.690276	-0.363314
H	3.002743	2.576258	0.373810
C	-1.121213	1.632674	3.778951
H	-2.166660	1.908214	3.677478
H	-1.081098	0.634274	4.204746
H	-0.653795	2.317599	4.475809
Br	1.642871	3.647309	3.175415
Br	-0.212513	-5.291842	1.417544
Br	-6.013565	1.444076	-2.087986
H	5.641577	-0.600561	0.181267
Cl	8.311409	0.040321	-0.540698

T-TS1

Cu	0.509623	0.381215	-0.894992
B	-1.412981	-0.548677	1.325891
H	-2.108494	-0.879218	2.228172
C	-2.980638	-1.950698	-0.236317
C	-2.972346	-2.206428	-1.590562
C	-1.851641	-1.551178	-2.119466
N	-1.226514	-0.942251	-1.133377
N	-1.905756	-1.180075	0.010323
N	1.010036	-0.718333	0.697790
C	2.126403	-1.315362	1.083457
C	1.860763	-1.998292	2.271280
C	0.527803	-1.786511	2.564796
N	0.038979	-1.006202	1.591300
N	-0.703237	1.605093	0.228885
C	-1.028524	2.886540	0.216762
C	-1.981254	3.105368	1.214050
C	-2.220240	1.883904	1.810404
N	-1.431067	0.993517	1.190993
O	1.033722	0.824154	-2.568470
C	3.220963	1.446188	-3.729738
C	4.432578	0.971661	-3.298901
C	4.854125	1.247764	-1.995355
C	4.074563	2.008144	-1.132927
C	2.855717	2.487274	-1.569376
C	2.349236	2.151828	-2.851932
H	2.890842	1.245598	-4.734543
H	4.437331	2.241106	-0.146427
H	2.277399	3.123270	-0.921486
H	1.596766	2.783955	-3.286399
C	-3.943797	-2.395198	0.806527
H	-4.435088	-1.549823	1.279975
H	-4.705053	-3.019649	0.354710
H	-3.449862	-2.969207	1.585144
C	-1.364904	-1.489033	-3.523023
H	-1.067491	-2.474662	-3.872650
H	-2.150321	-1.134145	-4.184807
H	-0.515612	-0.817163	-3.591917
C	-0.452732	3.860554	-0.746825
H	-1.226466	4.531241	-1.106531
H	0.314549	4.473946	-0.279689
H	-0.025655	3.338482	-1.594598
C	-3.155994	1.542041	2.914945
H	-3.617411	2.447031	3.290973
H	-3.942940	0.875143	2.574875
H	-2.638173	1.053543	3.734293
C	3.395393	-1.236927	0.315431
H	3.238316	-0.759313	-0.644285
H	4.148951	-0.674196	0.859688

H	3.794384	-2.232614	0.146264
C	-0.279047	-2.293770	3.706400
H	-0.742837	-1.479583	4.254243
H	-1.068396	-2.958155	3.366965
H	0.360451	-2.844733	4.385114
Br	3.092003	-3.006891	3.260008
Br	-4.212506	-3.231141	-2.556677
Br	-2.776318	4.748451	1.641680
H	5.069953	0.404807	-3.955439
Cl	6.393766	0.649126	-1.453129

T-I2

Cu	0.625443	0.260298	-0.674504
B	-1.752610	-0.412603	1.131587
H	-2.637332	-0.653678	1.883905
C	-3.162162	-1.608349	-0.723576
C	-2.932118	-1.867754	-2.057707
C	-1.661356	-1.357953	-2.356366
N	-1.162032	-0.827077	-1.258961
N	-2.067323	-0.975102	-0.266073
N	0.713269	-0.857143	0.968973
C	1.676958	-1.532665	1.573964
C	1.120235	-2.174148	2.681363
C	-0.222318	-1.848158	2.700037
N	-0.438736	-1.047172	1.647949
N	-0.567496	1.633246	0.260440
C	-0.673117	2.950714	0.270945
C	-1.751455	3.294149	1.089015
C	-2.288664	2.111270	1.557057
N	-1.548640	1.120718	1.037382
O	1.561092	0.634873	-2.182121
C	3.580628	0.741664	-3.399821
C	4.886621	0.464749	-3.173860
C	5.487444	0.836188	-1.950867
C	4.742202	1.479738	-0.940843
C	3.432903	1.771629	-1.143663
C	2.691018	1.427654	-2.403871
H	3.110405	0.442063	-4.322143
H	5.229632	1.736311	-0.014744
H	2.873742	2.277855	-0.371126
H	2.357333	2.372857	-2.860649
C	-4.349876	-1.927634	0.113050
H	-4.816115	-1.026564	0.501493
H	-5.082101	-2.457274	-0.484230
H	-4.082947	-2.553524	0.959570
C	-0.916005	-1.361633	-3.642826
H	-0.682936	-2.378259	-3.949956
H	-1.514650	-0.914692	-4.431975
H	0.006431	-0.800954	-3.536482
C	0.241046	3.838719	-0.494069
H	-0.316207	4.652285	-0.946963
H	0.993501	4.281302	0.154713
H	0.738629	3.281792	-1.279299
C	-3.456649	1.896184	2.452514
H	-3.849132	2.854010	2.771445
H	-4.249667	1.355100	1.944682
H	-3.179156	1.327651	3.334676
C	3.080332	-1.558791	1.085818
H	3.205717	-0.918293	0.220392
H	3.761981	-1.225606	1.863253
H	3.368880	-2.569979	0.812134
C	-1.281375	-2.263284	3.657925
H	-1.750486	-1.402522	4.124272
H	-2.058388	-2.837155	3.161616
H	-0.846072	-2.879827	4.434809
Br	2.038967	-3.264275	3.896804

Br	-4.085259	-2.734723	-3.257003
Br	-2.342767	5.032380	1.466587
H	5.480230	-0.046649	-3.913105
Cl	7.160188	0.479102	-1.677265

MECP1

Cu	0.72668313	0.25967817	-0.74840573
B	-1.62756223	-0.40855002	1.05433347
H	-2.52234228	-0.67987761	1.78537445
C	-3.02215352	-1.60857421	-0.80277568
C	-2.79629592	-1.87293693	-2.13653487
C	-1.52883866	-1.35940755	-2.43902480
N	-1.02646252	-0.82128345	-1.34751717
N	-1.92686330	-0.96978914	-0.35230966
N	0.84886796	-0.86519354	0.94840310
C	1.78092481	-1.53996840	1.59599875
C	1.18958808	-2.15682829	2.70173170
C	-0.14723732	-1.81831928	2.67661815
N	-0.32253179	-1.03474549	1.60017417
N	-0.53750736	1.69993660	0.20187293
C	-0.68275390	3.00813297	0.28207594
C	-1.74172718	3.28813662	1.15124720
C	-2.22909602	2.07282041	1.58196102
N	-1.47744447	1.12982214	0.98764646
O	1.67699589	0.84578350	-2.33708238
C	3.24643259	0.79979919	-3.32670927
C	4.32269464	0.02402155	-2.86801641
C	4.91365239	0.35699265	-1.68518370
C	4.48192815	1.47836398	-0.89701265
C	3.43818316	2.22060868	-1.29480670
C	2.65008911	1.85728096	-2.50396416
H	2.82914759	0.63558731	-4.30238637
H	5.03671687	1.72132926	-0.00737064
H	3.14920575	3.09524014	-0.73590616
H	2.25161451	2.72367996	-3.02367741
C	-4.20927617	-1.92661956	0.03541177
H	-4.67300885	-1.02467400	0.42468808
H	-4.94340078	-2.45497179	-0.56114457
H	-3.94262115	-2.55246967	0.88190461
C	-0.78754501	-1.36987204	-3.72788497
H	-0.56253307	-2.38725698	-4.03884016
H	-1.37765779	-0.91292734	-4.51792644
H	0.13945358	-0.81810255	-3.62051749
C	0.17454264	3.96046404	-0.47076366
H	-0.42817496	4.74378837	-0.92023916
H	0.89930976	4.44312430	0.18175679
H	0.70204582	3.43690756	-1.25906458
C	-3.35742991	1.78782077	2.50851247
H	-3.77189648	2.72130249	2.87007180
H	-4.14892986	1.23333256	2.01315787
H	-3.03135642	1.20476026	3.36427919
C	3.19451033	-1.59278368	1.14380436
H	3.31317901	-1.03695887	0.22149033
H	3.86258712	-1.17372197	1.89170828
H	3.50500552	-2.61961702	0.97097155
C	-1.23444329	-2.20361256	3.61585631
H	-1.70185081	-1.32976519	4.05933672
H	-2.00986124	-2.77379657	3.11292404
H	-0.82510890	-2.81341303	4.41235091
Br	2.06596819	-3.23860457	3.96025433
Br	-3.94764256	-2.75335025	-3.33070977
Br	-2.36666906	4.99493924	1.62180796
H	4.68408019	-0.80324980	-3.45247593
Cl	6.26449596	-0.55135121	-1.10517190

S-I3

Cu	-0.202312	-0.000136	1.291184
B	1.534802	-0.000123	-1.165657
H	2.241092	-0.000125	-2.119547
C	3.760342	0.001160	0.195062
C	4.062434	0.001508	1.539368
C	2.841968	0.000897	2.224882
N	1.865992	0.000199	1.340615
N	2.417199	0.000385	0.106233
N	-0.193659	-1.559817	-0.194016
C	-0.721734	-2.744027	-0.433307
C	-0.204256	-3.230179	-1.639183
C	0.674740	-2.275282	-2.102544
N	0.657900	-1.275059	-1.202035
N	-0.194090	1.559327	-0.194377
C	-0.722881	2.743099	-0.434260
C	-0.206199	3.228670	-1.640713
C	0.673116	2.273923	-2.103775
N	0.657202	1.274328	-1.202552
O	-1.806668	0.000036	2.579452
C	-4.010130	0.000266	3.333153
C	-5.313592	0.000306	3.018999
C	-5.773452	0.000045	1.642011
C	-4.918857	-0.000281	0.623554
C	-3.448908	-0.000409	0.857576
C	-2.997639	-0.000021	2.292909
H	-3.669027	0.000479	4.354126
H	-5.269712	-0.000478	-0.394578
H	-2.985817	-0.857417	0.366330
H	-2.985529	0.856098	0.365739
C	4.679160	0.001525	-0.975108
H	4.529183	0.878333	-1.598194
H	5.705587	0.002120	-0.628228
H	4.530128	-0.875580	-1.598000
C	2.588361	0.000923	3.689435
H	3.026547	-0.874772	4.161173
H	3.025753	0.877100	4.161013
H	1.520526	0.000453	3.879554
C	-1.687031	3.391996	0.494977
H	-1.357215	4.394749	0.751768
H	-2.674344	3.486831	0.047371
H	-1.765966	2.815893	1.410759
C	1.506515	2.282831	-3.336210
H	1.295978	3.178546	-3.908374
H	2.566534	2.271494	-3.099751
H	1.299424	1.420305	-3.962390
C	-1.685997	-3.392754	0.495930
H	-1.765433	-2.816207	1.411390
H	-2.673127	-3.488127	0.048031
H	-1.355955	-4.395270	0.753350
C	1.508714	-2.284639	-3.334587
H	1.301815	-1.422429	-3.961257
H	2.568623	-2.273063	-3.097624
H	1.298576	-3.180652	-3.906431
Br	-0.632455	-4.870680	-2.447114
Br	5.770321	0.002562	2.320403
Br	-0.635603	4.868389	-2.449593
H	-6.060841	0.000546	3.795225
Cl	-7.493030	0.000180	1.391849

C_6H_5ClO

O	-1.803369	0.000012	2.566492
C	-4.016092	0.000274	3.337963
C	-5.315361	0.000315	3.025670
C	-5.767445	0.000038	1.641146
C	-4.917751	-0.000290	0.621028

C	-3.443574	-0.000416	0.852513
C	-2.980539	-0.000049	2.298258
H	-3.675643	0.000496	4.359539
H	-5.276819	-0.000485	-0.394569
H	-2.989152	-0.864808	0.367316
H	-2.988892	0.863528	0.366769
H	-6.068341	0.000563	3.796374
Cl	-7.490224	0.000172	1.387847

C_6H_4ClOH

C	-1.774152	1.292300	-0.057703
C	-0.388767	1.242068	-0.144641
C	0.345683	2.419119	-0.140778
C	-0.299194	3.642195	-0.050532
C	-1.677689	3.682519	0.035807
C	-2.418292	2.511031	0.032555
H	-2.330004	0.370406	-0.061882
H	1.422458	2.387143	-0.208291
H	0.268878	4.556403	-0.047457
H	-3.491679	2.553969	0.100181
Cl	-2.492366	5.219541	0.149756
O	0.186059	0.021389	-0.230317
H	1.134058	0.107110	-0.285964

T-TS2

B	-1.927604	0.002322	1.125406
H	-2.771949	-0.012331	1.958164
C	-3.699729	-0.998141	-0.524609
C	-3.649803	-1.348631	-1.857045
C	-2.356944	-1.049298	-2.306843
N	-1.676851	-0.545662	-1.297200
N	-2.484720	-0.512348	-0.214208
N	0.372895	-0.974789	0.797373
C	1.200648	-1.837118	1.361180
C	0.604224	-2.308539	2.533239
C	-0.623673	-1.686656	2.634813
N	-0.736682	-0.881917	1.567306
N	-0.409884	1.689305	0.056340
C	-0.176951	2.990790	0.024651
C	-1.056491	3.605787	0.916080
C	-1.826345	2.601794	1.473591
N	-1.408701	1.450137	0.931217
O	1.336228	-0.234247	-2.399320
C	3.754378	-0.005748	-2.035111
C	4.293089	1.058629	-1.345559
C	5.667792	1.104505	-1.151038
C	6.455191	0.083186	-1.655854
C	5.904593	-0.981976	-2.350037
C	4.530220	-1.026316	-2.541762
H	2.445219	-0.084521	-2.282979
H	3.670801	1.849937	-0.960978
H	6.122780	1.921387	-0.617463
H	4.081330	-1.845205	-3.079101
C	-1.757920	-1.226618	-3.656019
H	-2.262928	-0.600179	-4.387115
H	-0.704733	-0.966810	-3.634652
H	-1.859844	-2.256575	-3.986801
C	-4.825302	-1.104963	0.441955
H	-5.701267	-1.493951	-0.062889
H	-4.581797	-1.773620	1.262878
H	-5.077293	-0.137681	0.866576
C	0.862499	3.600888	-0.845456
H	0.438611	4.405922	-1.438187
H	1.666331	4.025304	-0.249056

H	1.279824	2.862540	-1.522313
C	-2.921346	2.697014	2.475251
H	-3.044608	3.728688	2.781121
H	-3.863350	2.348084	2.062627
H	-2.702927	2.100881	3.355775
C	2.522316	-2.188099	0.780892
H	2.613969	-1.800457	-0.226006
H	3.330664	-1.781177	1.383210
H	2.647835	-3.266330	0.753319
C	-1.669423	-1.831180	3.682571
H	-1.897212	-0.877373	4.148453
H	-2.592024	-2.227068	3.268324
H	-1.321223	-2.512334	4.449363
Br	1.348104	-3.547134	3.727252
Br	-5.034915	-2.083721	-2.886572
Br	-1.156653	5.442164	1.272981
H	6.540987	-1.761045	-2.733576
Cl	8.180048	0.140312	-1.412542

T-I4

Cu	-0.773644	0.078041	-1.856919
B	-0.950602	0.020882	1.184151
H	-1.060192	0.014711	2.364805
C	0.047868	-2.394934	1.393864
C	0.477861	-3.320791	0.465613
C	0.297322	-2.744265	-0.797259
N	-0.216123	-1.540631	-0.627714
N	-0.369719	-1.323363	0.698357
N	0.116948	1.479369	-0.570086
C	0.948342	2.499379	-0.671816
C	1.339928	2.877723	0.615180
C	0.702156	2.024358	1.491797
N	-0.036215	1.187901	0.743653
N	-2.454209	0.253398	-0.815961
C	-3.727547	0.417350	-1.132085
C	-4.458069	0.491483	0.054160
C	-3.551305	0.362136	1.089756
N	-2.342851	0.219513	0.528819
O	0.120773	-0.165027	-3.409699
C	4.506781	0.149760	-2.694337
C	5.658769	0.363653	-1.983661
C	5.589103	0.215377	-0.601268
C	4.380098	-0.136162	-0.022133
C	3.237088	-0.345234	-0.776531
C	3.293323	-0.199604	-2.161715
H	0.400547	0.625982	-3.859607
H	6.586941	0.635440	-2.458732
H	6.458454	0.370371	0.014826
H	2.404542	-0.349237	-2.756770
C	0.597923	-3.296587	-2.144441
H	-0.067913	-4.124160	-2.377363
H	0.486382	-2.523939	-2.897516
H	1.614064	-3.679028	-2.178373
C	0.025334	-2.483031	2.878323
H	0.410101	-3.446478	3.189519
H	0.640601	-1.709767	3.328666
H	-0.983071	-2.375112	3.266880
C	-4.202650	0.497255	-2.538328
H	-4.937397	-0.276950	-2.739901
H	-4.676612	1.455696	-2.731213
H	-3.377501	0.374406	-3.232474
C	-3.787961	0.368129	2.557805
H	-4.845140	0.497975	2.754190
H	-3.466697	-0.564250	3.011990
H	-3.247391	1.176709	3.040316
C	1.360849	3.075574	-1.978051

H	0.718699	2.712566	-2.772456
H	1.306091	4.159486	-1.954645
H	2.385533	2.798298	-2.211591
C	0.777851	1.969340	2.975844
H	-0.207069	2.030913	3.427711
H	1.243590	1.046050	3.308761
H	1.375714	2.796714	3.337923
Br	2.506692	4.279601	1.046536
Br	1.170241	-5.028749	0.809588
Br	-6.312116	0.721813	0.191784
H	2.311720	-0.614206	-0.298309
Cl	4.295099	-0.317740	1.713544

T-TS3

Cu	0.284329	0.107037	-1.238902
B	-1.238026	-0.139573	1.398499
H	-1.834110	-0.183337	2.421844
C	-3.454953	-0.922412	0.261420
C	-3.834039	-1.101189	-1.052547
C	-2.726432	-0.777550	-1.843573
N	-1.737638	-0.424891	-1.039582
N	-2.177862	-0.510077	0.235706
N	0.866776	-1.122907	0.447795
C	1.811341	-1.987296	0.775161
C	1.503144	-2.527580	2.027857
C	0.327006	-1.938135	2.436848
N	-0.033933	-1.091769	1.458291
N	-0.091412	1.647183	0.015656
C	0.108299	2.960283	0.035688
C	-0.401793	3.460438	1.233021
C	-0.927944	2.385919	1.923111
N	-0.729222	1.304729	1.161078
O	0.574139	-0.973181	-2.782203
C	2.004482	-0.176739	-3.444543
C	3.105731	-1.042151	-3.590943
C	4.325798	-0.700361	-3.058270
C	4.469424	0.478507	-2.316247
C	3.408386	1.340925	-2.165848
C	2.164984	1.027926	-2.727955
H	0.749264	-1.899787	-2.613352
H	3.013952	-1.946891	-4.172660
H	3.558496	2.275255	-1.649634
H	1.420713	1.807265	-2.811987
Br	-0.386063	5.247930	1.797398
Br	2.515366	-3.801881	2.958691
Br	-5.511490	-1.660271	-1.676933
C	-2.613915	-0.778564	-3.326368
H	-3.088976	-1.663594	-3.738519
H	-3.122362	0.085849	-3.747963
H	-1.572997	-0.762675	-3.623831
C	-4.240050	-1.127622	1.508021
H	-5.239046	-1.461555	1.255503
H	-3.781443	-1.877207	2.146221
H	-4.321564	-0.208995	2.081388
C	0.745713	3.729485	-1.066494
H	0.645118	4.790536	-0.874112
H	1.803381	3.504926	-1.149466
H	0.272554	3.516001	-2.019653
C	-1.600524	2.360588	3.249654
H	-1.571302	3.350564	3.687820
H	-2.639410	2.057264	3.160618
H	-1.108861	1.670201	3.927413
C	2.987071	-2.276609	-0.083493
H	3.903980	-1.953823	0.402661
H	3.075767	-3.344609	-0.264087
H	2.911984	-1.764750	-1.033207

C	-0.446046	-2.146178	3.690587
H	0.054988	-2.884264	4.304991
H	-0.531221	-1.226921	4.262425
H	-1.451007	-2.501500	3.482797
H	5.185056	-1.332862	-3.212802
Cl	6.024942	0.868587	-1.620159

T-I5

Cu	0.392216	0.246140	-0.699471
B	-2.203126	-0.083109	0.879066
H	-3.196536	-0.171766	1.522302
C	-3.563228	1.427297	-0.779524
C	-3.225927	2.103824	-1.933757
C	-1.865097	1.868646	-2.159835
N	-1.420127	1.095584	-1.188061
N	-2.444107	0.823403	-0.347141
N	-0.571656	-1.625715	-0.248692
C	-0.409468	-2.904783	-0.526445
C	-1.509461	-3.608067	-0.023898
C	-2.336096	-2.678649	0.571155
N	-1.738454	-1.484314	0.419620
N	0.142089	0.759237	1.197287
C	0.940193	1.262264	2.124181
C	0.205320	1.378236	3.304207
C	-1.067193	0.912575	3.029815
N	-1.075210	0.545831	1.741098
O	1.442877	0.200670	-2.172726
C	4.880733	-1.209028	-1.052054
C	6.092802	-1.706044	-0.656950
C	7.197898	-0.901467	-0.914345
C	7.010417	0.327014	-1.539758
C	5.754000	0.778346	-1.919849
C	4.640669	-0.020027	-1.666099
H	1.529344	-0.623119	-2.636362
H	6.202694	-2.661396	-0.170992
H	5.647013	1.733865	-2.404446
H	3.634471	0.271092	-1.933316
Br	0.836912	2.048311	4.935559
Br	-1.783098	-5.459779	-0.138581
Br	-4.359549	3.144530	-3.005000
C	-0.986413	2.350569	-3.258170
H	-1.461946	2.187901	-4.220888
H	-0.803911	3.418571	-3.163720
H	-0.038550	1.822876	-3.232221
C	-4.878659	1.322226	-0.093562
H	-5.607614	1.934748	-0.610009
H	-5.238945	0.297301	-0.087601
H	-4.814613	1.657749	0.936904
C	2.357857	1.621800	1.857223
H	2.509947	2.692444	1.963604
H	3.018275	1.126059	2.562258
H	2.651935	1.333342	0.853150
C	-2.248469	0.803881	3.926056
H	-1.996201	1.185623	4.907551
H	-3.088543	1.373122	3.540436
H	-2.566047	-0.229138	4.030827
C	0.778297	-3.426234	-1.253762
H	1.277359	-4.197071	-0.673197
H	0.488164	-3.872054	-2.201524
H	1.488487	-2.629589	-1.444809
C	-3.635657	-2.877896	1.267006
H	-3.904867	-3.926819	1.239960
H	-3.579105	-2.566749	2.305999
H	-4.430532	-2.309648	0.793640
H	8.188997	-1.217413	-0.636394
Cl	8.402760	1.331763	-1.853989

MECP2

Cu	0.09657909	-0.19523861	-1.29417759
B	-1.16171367	0.22829439	1.47211385
H	-1.67888432	0.37298169	2.52888218
C	-3.40971068	-0.86755102	0.67426860
C	-3.82174644	-1.41812473	-0.52503799
C	-2.74961168	-1.30913312	-1.41011149
N	-1.75152275	-0.72296675	-0.76615645
N	-2.14861611	-0.45521309	0.49491309
N	0.77820915	-1.06167391	0.51510124
C	1.78321122	-1.82055905	0.91635275
C	1.73014397	-1.91580964	2.31040239
C	0.64330817	-1.17545836	2.72187578
N	0.08286390	-0.66889261	1.61167699
N	-0.27097754	1.69555384	-0.36902966
C	-0.03757013	2.97187573	-0.62188285
C	-0.40158623	3.71718169	0.50333066
C	-0.86548808	2.82067228	1.44231886
N	-0.77549882	1.60241209	0.88460185
O	0.63693854	-1.79752671	-3.71478801
C	1.82424406	-1.21744601	-3.35205540
C	3.02644684	-1.73094880	-3.67052282
C	4.22586754	-1.10839762	-3.20114439
C	4.09893072	-0.02401187	-2.31288044
C	2.90659412	0.51059782	-1.94644488
C	1.61643139	0.03707531	-2.56584688
H	0.80223720	-2.57977400	-4.23806555
H	3.09850121	-2.64052636	-4.25009893
H	2.87736504	1.30822914	-1.22346106
H	1.30238901	0.79411700	-3.30244682
Br	-0.28043527	5.57977070	0.67617632
Br	2.92091395	-2.87764212	3.39233145
Br	-5.49156588	-2.17104388	-0.91647804
C	-2.66453108	-1.73546999	-2.83096566
H	-2.96258366	-2.77536497	-2.92996288
H	-3.33934339	-1.14533863	-3.44581318
H	-1.65631775	-1.62758869	-3.21345795
C	-4.15625060	-0.72299255	1.95198100
H	-5.15404304	-1.12797654	1.83482741
H	-3.66270506	-1.25459897	2.75984628
H	-4.24132179	0.31969599	2.24259450
C	0.51572348	3.46839865	-1.90852506
H	-0.10268465	4.27001125	-2.30226963
H	1.51651210	3.86846047	-1.76836540
H	0.56498433	2.67305161	-2.63966767
C	-1.38026740	3.07727755	2.81392176
H	-1.35286824	4.14139414	3.01550195
H	-2.40472027	2.73468621	2.92335517
H	-0.77898965	2.57346239	3.56483186
C	2.76693318	-2.43804323	-0.00763461
H	3.74413346	-1.97951584	0.11384855
H	2.87038359	-3.49808018	0.20705212
H	2.46760709	-2.31299633	-1.03911537
C	0.13032756	-0.94149016	4.09790333
H	0.76280411	-1.45815752	4.80991089
H	0.12962772	0.11525866	4.34763288
H	-0.88455120	-1.31067239	4.21092972
H	5.19178192	-1.49490006	-3.46495413
Cl	5.57702884	0.60752487	-1.59932341

S-I6

Cu	0.398771	-0.125887	-1.167547
B	-0.914066	0.154392	1.485354

H	-1.430782	0.247174	2.548749
C	-2.990684	-1.286981	0.885610
C	-3.403945	-1.962264	-0.240829
C	-2.417293	-1.764343	-1.212940
N	-1.463877	-1.011813	-0.698681
N	-1.809780	-0.720487	0.577720
N	1.257719	-0.798493	0.614560
C	2.328826	-1.422171	1.066502
C	2.228378	-1.529183	2.457416
C	1.034208	-0.941659	2.814025
N	0.468367	-0.508919	1.673601
N	-0.296336	1.765795	-0.363418
C	-0.364980	3.058603	-0.622042
C	-0.889457	3.714291	0.496423
C	-1.140701	2.742052	1.439705
N	-0.771768	1.573005	0.887022
O	0.226991	-1.626169	-4.724184
C	1.276468	-1.082563	-4.077141
C	2.534678	-1.657984	-4.068953
C	3.591474	-1.017357	-3.435242
C	3.383922	0.187932	-2.798568
C	2.121477	0.771798	-2.787796
C	1.059940	0.137236	-3.426117
H	0.465935	-2.465841	-5.109368
H	2.705270	-2.594970	-4.575822
H	1.988099	1.739351	-2.339802
H	0.104479	0.623057	-3.541015
Br	-1.193896	5.559874	0.663111
Br	3.485259	-2.328164	3.600847
Br	-4.979512	-2.961511	-0.453769
C	-2.384690	-2.284255	-2.604330
H	-2.373346	-3.371842	-2.607580
H	-3.270416	-1.972017	-3.151689
H	-1.509757	-1.925929	-3.131396
C	-3.664739	-1.165961	2.206446
H	-4.600289	-1.712018	2.184704
H	-3.051303	-1.572015	3.005269
H	-3.880308	-0.129775	2.449548
C	0.057049	3.657238	-1.917613
H	-0.607957	4.469243	-2.193295
H	1.062055	4.070696	-1.859114
H	0.031560	2.918197	-2.710406
C	-1.708849	2.887303	2.807190
H	-1.880110	3.936454	3.016422
H	-2.654463	2.361976	2.903897
H	-1.034286	2.494541	3.561844
C	3.415289	-1.909864	0.178304
H	4.292122	-1.269917	0.239470
H	3.719371	-2.912294	0.464650
H	3.076022	-1.928275	-0.850193
C	0.430617	-0.788042	4.165244
H	1.106614	-1.191209	4.909777
H	0.245350	0.255312	4.401951
H	-0.514949	-1.316854	4.241523
H	4.572559	-1.460051	-3.443279
Cl	4.708330	0.998916	-2.019606

-CH3

$C_6H_5CH_3$

C	-1.694735	1.279486	-0.014645
C	-0.304064	1.300052	-0.010427
C	0.391410	2.498271	0.002239
C	-0.295094	3.702037	0.008469
C	-1.680698	3.695568	0.000838
C	-2.371444	2.494511	-0.011833

H	0.239138	0.367498	-0.019055
H	1.469688	2.491370	0.003334
H	0.244171	4.635342	0.015344
H	-3.450626	2.499392	-0.021554
H	-2.225246	4.626225	0.000830
C	-2.446391	-0.021949	0.003400
H	-1.889411	-0.808172	-0.497944
H	-2.630564	-0.354303	1.023855
H	-3.411294	0.071071	-0.486432

T-I1

Cu	-0.137783	-0.047277	-1.296941
B	1.360401	-0.154007	1.348496
H	1.918945	-0.216353	2.392944
C	3.472443	-1.300367	0.304356
C	3.797372	-1.702798	-0.974888
C	2.688513	-1.423019	-1.781018
N	1.753013	-0.890518	-1.019637
N	2.222656	-0.811103	0.245843
N	0.403681	1.617849	-0.148711
C	0.358578	2.929336	-0.278333
C	1.023921	3.497457	0.813419
C	1.474679	2.452243	1.593504
N	1.081752	1.322160	0.982791
N	-0.783890	-0.974084	0.337141
C	-1.869258	-1.665181	0.645141
C	-1.765422	-2.048944	1.983180
C	-0.564028	-1.551771	2.451367
N	0.009153	-0.903867	1.428336
O	-0.332569	0.451252	-3.022172
C	-3.729265	0.466673	-3.161269
C	-4.698294	-0.487840	-3.433466
C	-5.858677	-0.542621	-2.677492
C	-6.079649	0.354590	-1.636586
C	-5.100852	1.306818	-1.370425
C	-3.938920	1.364574	-2.125012
H	-2.812517	0.501210	-3.726232
H	-4.548244	-1.193905	-4.234652
H	-6.605037	-1.290545	-2.898028
H	-3.194131	2.112884	-1.905250
C	2.496297	-1.646946	-3.237953
H	2.357252	-2.703519	-3.454161
H	1.630240	-1.097189	-3.591020
H	3.368064	-1.312751	-3.791983
C	4.285941	-1.357678	1.548249
H	5.238806	-1.827314	1.336533
H	4.477151	-0.363882	1.942899
H	3.785598	-1.931329	2.322564
C	-2.959801	-1.933256	-0.327321
H	-3.199189	-2.992307	-0.345910
H	-3.863209	-1.393733	-0.057593
H	-2.678529	-1.621538	-1.326494
C	0.044529	-1.669380	3.803115
H	-0.618206	-2.229085	4.451675
H	0.999476	-2.184640	3.761929
H	0.212911	-0.692323	4.245681
C	-0.309413	3.592624	-1.428752
H	-0.498803	2.872734	-2.218043
H	-1.250700	4.045239	-1.125288
H	0.317482	4.384311	-1.827196
C	2.249401	2.486385	2.862777
H	1.729097	1.962503	3.658811
H	3.225402	2.024353	2.744240
H	2.396979	3.514739	3.169472
Br	1.248422	5.330662	1.138659
Br	5.407687	-2.478812	-1.541676

Br	-3.026960	-3.049435	2.941480
H	-5.252297	2.011253	-0.566776
C	-7.355821	0.315614	-0.843123
H	-8.131149	0.913832	-1.319176
H	-7.737681	-0.697476	-0.754960
H	-7.211903	0.709178	0.158813

T-TS1

Cu	-0.598058	0.402168	0.959039
B	1.204512	-0.549925	-1.360529
H	1.849393	-0.883867	-2.298679
C	2.795200	-2.025847	0.108137
C	2.835577	-2.304935	1.457066
C	1.759127	-1.624589	2.043982
N	1.113012	-0.979483	1.095441
N	1.735915	-1.217735	-0.079360
N	-1.195658	-0.656990	-0.633262
C	-2.344261	-1.213457	-0.980946
C	-2.148278	-1.889355	-2.186313
C	-0.822268	-1.715201	-2.531026
N	-0.271040	-0.963028	-1.568620
N	0.597912	1.604292	-0.204310
C	0.947457	2.879071	-0.195180
C	1.861186	3.089105	-1.230448
C	2.050113	1.869339	-1.847643
N	1.270681	0.988359	-1.202803
O	-1.050376	0.806141	2.655970
C	-3.134903	1.527445	3.961277
C	-4.394221	1.111000	3.621847
C	-4.936837	1.377818	2.352560
C	-4.161475	2.096566	1.443423
C	-2.890257	2.527951	1.769968
C	-2.300440	2.186484	3.013671
H	-2.738673	1.322389	4.941436
H	-4.574866	2.344900	0.477854
H	-2.336376	3.131665	1.070621
H	-1.493091	2.791955	3.383853
C	3.698766	-2.481256	-0.982305
H	4.203913	-1.644002	-1.455832
H	4.452301	-3.144907	-0.575644
H	3.152721	-3.016813	-1.753431
C	1.333025	-1.571247	3.467546
H	1.027033	-2.554647	3.816124
H	2.153762	-1.244490	4.100441
H	0.503701	-0.880499	3.580396
C	0.427930	3.854377	0.798510
H	1.227848	4.505089	1.137149
H	-0.343246	4.487469	0.365538
H	0.019273	3.332325	1.655150
C	2.930480	1.520130	-2.994614
H	3.402993	2.417887	-3.374214
H	3.710073	0.824687	-2.697660
H	2.366208	1.061312	-3.800468
C	-3.578633	-1.104483	-0.161831
H	-3.380400	-0.591151	0.771417
H	-4.351226	-0.559164	-0.697464
H	-3.970820	-2.093335	0.058617
C	-0.078024	-2.231867	-3.710363
H	0.388518	-1.425198	-4.266895
H	0.703385	-2.924309	-3.410952
H	-0.760893	-2.754354	-4.369128
Br	-3.450292	-2.846514	-3.135713
Br	4.082777	-3.384486	2.352420
Br	2.669481	4.720826	-1.677441
H	-4.994407	0.582738	4.346891
C	-6.314035	0.901113	1.998039

H	-7.017744	1.091162	2.804855
H	-6.686464	1.392307	1.104277
H	-6.324720	-0.172352	1.812454

T-I2

Cu	0.677792	0.305585	-0.833365
B	-1.406308	-0.439229	1.292212
H	-2.168657	-0.697833	2.163565
C	-2.955899	-1.811279	-0.313182
C	-2.879943	-2.118331	-1.654680
C	-1.694222	-1.545442	-2.135199
N	-1.096760	-0.933977	-1.133367
N	-1.856325	-1.091204	-0.026836
N	1.046031	-0.727072	0.831967
C	2.121540	-1.313482	1.331368
C	1.753449	-1.940886	2.522723
C	0.404900	-1.700311	2.702037
N	0.004191	-0.960339	1.659213
N	-0.493449	1.630073	0.183386
C	-0.694676	2.935684	0.143712
C	-1.682382	3.251185	1.079351
C	-2.065834	2.063376	1.669952
N	-1.326174	1.097258	1.105782
O	1.393732	0.647220	-2.459658
C	3.317491	0.730872	-3.832590
C	4.648635	0.546358	-3.682993
C	5.359561	1.047893	-2.558150
C	4.626695	1.739767	-1.568621
C	3.287342	1.951028	-1.676956
C	2.469011	1.464304	-2.836665
H	2.795434	0.323805	-4.683717
H	5.154314	2.112013	-0.702510
H	2.765332	2.500206	-0.907296
H	2.049757	2.346712	-3.346073
C	-4.003376	-2.163312	0.682612
H	-4.473909	-1.275318	1.095249
H	-4.769983	-2.762554	0.206379
H	-3.589772	-2.733061	1.509687
C	-1.119178	-1.562672	-3.506214
H	-0.857700	-2.575968	-3.801427
H	-1.841996	-1.192046	-4.228079
H	-0.231155	-0.940604	-3.541640
C	0.044565	3.837097	-0.778592
H	-0.627005	4.580293	-1.196169
H	0.836218	4.369953	-0.256845
H	0.481631	3.269208	-1.591600
C	-3.091265	1.820172	2.719610
H	-3.504891	2.765936	3.047789
H	-3.903768	1.205537	2.343108
H	-2.663947	1.314558	3.579873
C	3.450051	-1.272267	0.666762
H	3.426950	-0.642890	-0.215350
H	4.204586	-0.886304	1.346330
H	3.758211	-2.271820	0.371746
C	-0.494667	-2.140548	3.801361
H	-0.951938	-1.291861	4.300636
H	-1.292396	-2.775268	3.427148
H	0.074196	-2.702563	4.531887
Br	2.889568	-2.917845	3.648100
Br	-4.114623	-3.111287	-2.660042
Br	-2.349212	4.964194	1.447487
H	5.196948	-0.005572	-4.432476
C	6.836110	0.838615	-2.439111
H	7.374889	1.344312	-3.240285
H	7.217706	1.217467	-1.495875
H	7.095922	-0.217205	-2.504570

MECP1

Cu	0.80255349	0.32297204	-0.92873420
B	-1.25474254	-0.42792190	1.20232176
H	-2.02590291	-0.72571447	2.05480817
C	-2.77732386	-1.81297755	-0.41018539
C	-2.69651011	-2.13067755	-1.74844320
C	-1.51277314	-1.55472172	-2.22839929
N	-0.92033599	-0.93213812	-1.23166656
N	-1.68099838	-1.08548843	-0.12728286
N	1.22120867	-0.68734343	0.80681033
C	2.27758194	-1.25553244	1.35671952
C	1.87862934	-1.87494051	2.54449672
C	0.52382774	-1.64904640	2.67192741
N	0.15573832	-0.92549229	1.60144655
N	-0.46302179	1.70524672	0.11606012
C	-0.70339860	3.00063722	0.15497179
C	-1.65656507	3.24906223	1.14795160
C	-1.98010329	2.02683823	1.69714902
N	-1.23866842	1.11136746	1.04896634
O	1.49987195	0.83999281	-2.65849148
C	2.99264474	0.85725776	-3.84445854
C	4.20416140	0.26943056	-3.46101654
C	4.91217413	0.76551962	-2.39910039
C	4.39120972	1.89126936	-1.65737639
C	3.21734892	2.45847466	-1.96502330
C	2.34833074	1.90051081	-3.03865545
H	2.47672415	0.53782974	-4.73096698
H	4.99411755	2.29219112	-0.85759674
H	2.87443637	3.33077233	-1.43271023
H	1.81142789	2.67550023	-3.58116149
C	-3.83052318	-2.16035463	0.58117880
H	-4.30990541	-1.27055298	0.97983063
H	-4.58888752	-2.76911642	0.10365170
H	-3.42112913	-2.71826865	1.41820219
C	-0.93527603	-1.57954307	-3.59896466
H	-0.64969053	-2.58982433	-3.88359525
H	-1.66090441	-1.22994026	-4.32886492
H	-0.06120811	-0.93847284	-3.63699007
C	-0.03340673	3.96870425	-0.75272215
H	-0.74709814	4.69264591	-1.13381503
H	0.74683263	4.52349985	-0.23528949
H	0.40566487	3.44222049	-1.59196929
C	-2.94883193	1.71092421	2.78150096
H	-3.38728478	2.62999798	3.15166183
H	-3.75006529	1.07031378	2.42489047
H	-2.46525854	1.20449554	3.61123904
C	3.62715383	-1.20164146	0.73821194
H	3.58127949	-0.67068154	-0.20555262
H	4.33492926	-0.69711825	1.39120859
H	4.00931508	-2.20235020	0.55579351
C	-0.40741114	-2.09023122	3.74543145
H	-0.89046632	-1.24420725	4.22493099
H	-1.18703394	-2.73587431	3.35197247
H	0.14308447	-2.64090005	4.49905343
Br	2.98935794	-2.82886493	3.72066447
Br	-3.92227929	-3.14021762	-2.75155312
Br	-2.35258441	4.92767076	1.62194371
H	4.58819501	-0.56339580	-4.02704344
C	6.24088583	0.20824537	-2.00476007
H	7.00527797	0.98270898	-2.05215317
H	6.22337727	-0.15455851	-0.97837654
H	6.54359616	-0.60786090	-2.65117841

S-I3

Cu	0.407137	0.000348	-1.247538
B	-1.387030	0.000510	1.177318
H	-2.106922	0.000423	2.121390
C	-3.591011	-0.007298	-0.218140
C	-3.870084	-0.008712	-1.567300
C	-2.637754	-0.004449	-2.231897
N	-1.677152	-0.000653	-1.331364
N	-2.249464	-0.002259	-0.107248
N	0.361018	-1.552661	0.232946
C	0.900814	-2.727844	0.487418
C	0.372606	-3.212620	1.689281
C	-0.523803	-2.265476	2.134735
N	-0.506966	-1.271537	1.227603
N	0.359224	1.554764	0.231559
C	0.894403	2.732840	0.482341
C	0.360950	3.221315	1.680386
C	-0.533740	2.273277	2.127382
N	-0.510867	1.275286	1.224838
O	1.917881	-0.000714	-2.631983
C	4.044849	-0.001384	-3.582452
C	5.371970	-0.001369	-3.380839
C	5.993942	0.000170	-2.062331
C	5.204630	0.001766	-0.986009
C	3.718748	0.002072	-1.073625
C	3.132408	-0.000059	-2.457731
H	3.612305	-0.002602	-4.568445
H	5.633177	0.002983	0.004306
H	3.303067	-0.854609	-0.540871
H	3.303522	0.860910	-0.543941
C	-4.528949	-0.010367	0.936740
H	-4.392805	0.867167	1.562038
H	-5.549585	-0.014084	0.573088
H	-4.386589	-0.886644	1.562419
C	-2.357914	-0.003808	-3.691797
H	-2.783912	-0.881411	-4.171237
H	-2.790279	0.870440	-4.171670
H	-1.286746	0.000035	-3.861890
C	1.882046	3.372175	-0.428059
H	1.577100	4.384832	-0.676632
H	2.865983	3.439012	0.031927
H	1.958804	2.804078	-1.348919
C	-1.387472	2.286675	3.345793
H	-1.185046	3.183587	3.919067
H	-2.443454	2.276017	3.091886
H	-1.192038	1.425529	3.977692
C	1.887399	-3.368323	-0.423322
H	1.961461	-2.802632	-1.345878
H	2.872379	-3.432686	0.034764
H	1.583235	-4.382055	-0.668500
C	-1.373495	-2.275931	3.355991
H	-1.176400	-1.412846	3.984773
H	-2.430309	-2.266566	3.105627
H	-1.168546	-3.171025	3.931204
Br	0.809091	-4.843037	2.514192
Br	-5.565002	-0.014994	-2.377190
Br	0.789816	4.856692	2.499453
H	6.029576	-0.002583	-4.237767
C	7.491233	-0.000169	-1.988945
H	7.907825	0.874412	-2.484858
H	7.834150	0.001616	-0.960039
H	7.907301	-0.876810	-2.481653

$C_6H_5(CH_3)O$

O	1.916805	-0.000766	-2.618949
C	4.051877	-0.001517	-3.588184

C	5.374194	-0.001473	-3.387901
C	5.990437	0.000259	-2.061032
C	5.204581	0.001839	-0.984216
C	3.715184	0.002176	-1.068062
C	3.115766	0.000679	-2.461180
H	3.619273	-0.003050	-4.574657
H	5.640814	0.002932	0.003506
H	3.305200	-0.860286	-0.540831
H	3.305816	0.866411	-0.543255
H	6.038285	-0.002880	-4.240226
C	7.487546	-0.000096	-1.987310
H	7.904442	0.874162	-2.484388
H	7.832461	0.001328	-0.958719
H	7.903902	-0.876090	-2.481780

$C_6H_4(CH_3)OH$

C	-1.694577	1.279765	-0.019457
C	-0.304070	1.303917	-0.017531
C	0.394029	2.499980	-0.013326
C	-0.295617	3.701269	-0.014466
C	-1.680704	3.698659	-0.014640
C	-2.368191	2.496363	-0.018961
H	0.242453	0.373414	-0.018924
H	1.471500	2.514882	-0.004086
H	-3.447273	2.505046	-0.021423
H	-2.206106	4.639442	-0.006315
C	-2.446186	-0.021600	0.009131
H	-1.891219	-0.810574	-0.490211
H	-2.628003	-0.349670	1.031379
H	-3.412371	0.068305	-0.478856
O	0.391625	4.890412	0.028051
H	0.562524	5.193058	-0.860191

T-TS2

Cu	0.472312	0.129984	-1.002884
B	-1.657650	-0.009372	1.196701
H	-2.441892	-0.028531	2.086514
C	-3.542065	-0.996992	-0.332686
C	-3.584257	-1.340375	-1.667179
C	-2.323961	-1.042077	-2.202671
N	-1.575161	-0.545899	-1.239262
N	-2.307157	-0.516169	-0.103778
N	0.612626	-0.986317	0.703488
C	1.478985	-1.849068	1.204954
C	0.966046	-2.324722	2.414570
C	-0.252112	-1.704672	2.604010
N	-0.440228	-0.896951	1.549396
N	-0.216641	1.683265	0.034515
C	0.011959	2.984995	-0.008430
C	-0.805096	3.595353	0.943978
C	-1.533394	2.588364	1.549416
N	-1.152621	1.439346	0.975036
O	1.358762	-0.201483	-2.550080
C	3.791318	0.036456	-2.303196
C	4.352103	1.080826	-1.601580
C	5.733976	1.120856	-1.461656
C	6.541412	0.134163	-2.017672
C	5.935358	-0.905679	-2.717496
C	4.556421	-0.962855	-2.865685
H	2.482987	-0.046222	-2.490925
H	3.740789	1.854623	-1.165992
H	6.188560	1.931411	-0.912521
H	4.093553	-1.771970	-3.406596
C	-1.818306	-1.213579	-3.590343

H	-2.374104	-0.586554	-4.283075
H	-0.766897	-0.950308	-3.640336
H	-1.939582	-2.242891	-3.916759
C	-4.598834	-1.106096	0.708465
H	-5.510276	-1.484807	0.261941
H	-4.303666	-1.784204	1.504356
H	-4.813990	-0.141421	1.158437
C	0.987961	3.599606	-0.946205
H	0.519714	4.398632	-1.513285
H	1.824913	4.032999	-0.404439
H	1.367464	2.861710	-1.645238
C	-2.556794	2.678189	2.624606
H	-2.659161	3.708394	2.942967
H	-3.524906	2.330367	2.276466
H	-2.277956	2.078259	3.485300
C	2.757360	-2.196875	0.532968
H	2.788424	-1.790367	-0.470116
H	3.605690	-1.805987	1.089030
H	2.872819	-3.275280	0.477648
C	-1.221710	-1.852943	3.722107
H	-1.413146	-0.901327	4.208469
H	-2.172541	-2.243776	3.371876
H	-0.822378	-2.539599	4.458502
Br	1.793059	-3.566457	3.549703
Br	-5.039165	-2.066577	-2.603027
Br	-0.882895	5.430571	1.313811
H	6.548507	-1.681133	-3.151086
C	8.037741	0.201794	-1.889191
H	8.482595	0.703158	-2.747195
H	8.334705	0.751837	-1.001271
H	8.475510	-0.790557	-1.831207

T-I4

Cu	0.158811	0.085520	-1.278004
B	-1.568641	0.132113	1.251439
H	-2.252523	0.195656	2.218705
C	-3.522187	-1.193848	0.114025
C	-3.691659	-1.725562	-1.147891
C	-2.546268	-1.400239	-1.883982
N	-1.743492	-0.707493	-1.099387
N	-2.327765	-0.579283	0.112076
N	0.710214	-0.744514	0.641642
C	1.699135	-1.441650	1.166806
C	1.348756	-1.786420	2.477186
C	0.096540	-1.257675	2.706001
N	-0.264640	-0.629853	1.573948
N	-0.429853	1.725626	-0.317593
C	-0.246064	3.019532	-0.517091
C	-0.919108	3.709033	0.492173
C	-1.512447	2.755995	1.298917
N	-1.196729	1.561727	0.779950
O	0.967575	-0.612947	-2.727763
C	4.547615	-1.465454	-3.166699
C	5.610354	-1.992789	-2.480845
C	6.398562	-1.099699	-1.761090
C	6.115322	0.264459	-1.748626
C	5.017449	0.729778	-2.466864
C	4.208323	-0.138267	-3.197645
H	1.758979	-1.116922	-2.567735
H	5.834126	-3.047063	-2.488985
H	4.787427	1.784570	-2.459259
H	3.346596	0.220250	-3.735686
Br	-0.991064	5.570502	0.693911
Br	2.398241	-2.775404	3.677162
Br	-5.168024	-2.697133	-1.775528
C	-2.197019	-1.720034	-3.293249

H	-2.359935	-2.775310	-3.492631
H	-2.826153	-1.159957	-3.981284
H	-1.155829	-1.478123	-3.480843
C	-4.426273	-1.249527	1.293733
H	-5.341204	-1.763360	1.025032
H	-3.966874	-1.784050	2.120398
H	-4.682216	-0.254177	1.644147
C	0.554282	3.550856	-1.651470
H	-0.047236	4.211229	-2.269645
H	1.401795	4.125664	-1.288399
H	0.925110	2.742283	-2.273036
C	-2.348419	2.935922	2.515493
H	-2.459777	3.992480	2.726317
H	-3.336612	2.506703	2.381012
H	-1.894152	2.459832	3.379182
C	2.945032	-1.763551	0.423063
H	3.819849	-1.421438	0.968455
H	3.046765	-2.837050	0.287481
H	2.946219	-1.292043	-0.551852
C	-0.747956	-1.326301	3.928724
H	-0.224897	-1.880277	4.698734
H	-0.972492	-0.335714	4.312952
H	-1.691642	-1.826439	3.731286
H	7.245369	-1.470835	-1.203632
C	6.998216	1.219423	-0.993434
H	7.846750	1.532517	-1.599443
H	6.456648	2.115195	-0.704870
H	7.395408	0.763166	-0.091306

T-TS3

Cu	-0.314522	0.178158	1.370736
B	0.904754	-0.231045	-1.432869
H	1.398025	-0.333137	-2.505844
C	3.107167	-1.284008	-0.509930
C	3.578884	-1.534682	0.761145
C	2.590383	-1.106994	1.654375
N	1.579863	-0.627917	0.948663
N	1.892658	-0.731637	-0.362121
N	-1.188976	-0.995290	-0.287824
C	-2.231160	-1.775026	-0.515286
C	-2.103970	-2.327898	-1.794224
C	-0.930693	-1.838546	-2.323389
N	-0.400563	-1.034771	-1.386497
N	0.177414	1.675205	0.068518
C	0.181272	3.003439	0.071408
C	0.598351	3.441935	-1.185554
C	0.863282	2.313369	-1.934785
N	0.605273	1.261821	-1.148445
O	-0.582913	-0.887945	2.939442
C	-1.865472	0.163004	3.613274
C	-3.081393	-0.508795	3.837591
C	-4.243573	0.000882	3.314040
C	-4.264630	1.143985	2.486423
C	-3.069855	1.792738	2.271651
C	-1.859027	1.330968	2.824390
H	-0.919482	-1.770910	2.782706
H	-3.111104	-1.384121	4.469075
H	-3.069653	2.703132	1.689604
H	-1.022698	2.012662	2.892527
Br	0.779559	5.221708	-1.748115
Br	-3.314556	-3.503850	-2.612958
Br	5.231338	-2.292749	1.222363
C	2.615487	-1.128446	3.141212
H	3.012698	-2.074610	3.496377
H	3.265645	-0.343553	3.521908
H	1.616659	-0.990693	3.535530

C	3.750589	-1.545920	-1.825253
H	4.719057	-2.005174	-1.668796
H	3.150317	-2.215291	-2.434307
H	3.894851	-0.627304	-2.386317
C	-0.156313	3.848597	1.249195
H	0.156568	4.868704	1.061820
H	-1.222281	3.861059	1.449698
H	0.355924	3.499981	2.139770
C	1.348784	2.210090	-3.337142
H	1.390518	3.198545	-3.777946
H	2.342739	1.774794	-3.380926
H	0.688767	1.593063	-3.938249
C	-3.326452	-1.986819	0.463708
H	-4.273716	-1.641115	0.057965
H	-3.440073	-3.044717	0.687262
H	-3.136974	-1.450543	1.383111
C	-0.312439	-2.101312	-3.650735
H	-0.947019	-2.771574	-4.217844
H	-0.188382	-1.185088	-4.220222
H	0.665404	-2.563117	-3.549703
H	-5.180240	-0.488066	3.543832
C	-5.557782	1.633824	1.898850
H	-6.316883	1.775648	2.666028
H	-5.422123	2.584244	1.390940
H	-5.966151	0.932404	1.172458

T-I5

Cu	0.215979	-0.196257	-1.329685
B	-0.930034	0.179878	1.501443
H	-1.397574	0.294524	2.585349
C	-3.139057	-1.068424	0.846020
C	-3.584760	-1.659680	-0.320420
C	-2.570615	-1.502757	-1.266932
N	-1.574045	-0.851130	-0.689744
N	-1.915492	-0.586992	0.588695
N	1.052954	-0.968534	0.457656
C	2.116350	-1.667296	0.812401
C	2.133501	-1.761657	2.207882
C	1.023737	-1.086611	2.668043
N	0.385937	-0.617394	1.583358
N	-0.259652	1.701948	-0.387699
C	-0.161961	2.991914	-0.656022
C	-0.525764	3.711090	0.486990
C	-0.850908	2.783644	1.453137
N	-0.681258	1.575069	0.892336
O	0.671137	-1.677584	-4.064998
C	1.806068	-1.073432	-3.604097
C	3.046998	-1.567378	-3.823966
C	4.190761	-0.886359	-3.329466
C	4.036315	0.307388	-2.588646
C	2.785442	0.806083	-2.361179
C	1.538889	0.149319	-2.828442
H	0.904852	-2.436135	-4.595619
H	3.171464	-2.486629	-4.379962
H	2.682252	1.728252	-1.809664
H	0.943589	0.829133	-3.452098
Br	-0.564962	5.579216	0.650261
Br	3.425641	-2.649635	3.237106
Br	-5.229195	-2.513628	-0.601239
C	-2.537499	-1.946649	-2.685268
H	-2.780138	-3.003251	-2.755553
H	-3.276669	-1.405791	-3.270970
H	-1.559217	-1.785478	-3.124692
C	-3.817299	-0.948121	2.163966
H	-4.790725	-1.420633	2.115427
H	-3.241290	-1.428937	2.948774

H	-3.955114	0.092266	2.442726
C	0.257910	3.527483	-1.977840
H	-0.466197	4.251921	-2.339559
H	1.215707	4.036081	-1.904606
H	0.347178	2.731769	-2.705353
C	-1.306250	3.000986	2.852536
H	-1.352344	4.063955	3.055886
H	-2.292276	2.577987	3.020171
H	-0.625459	2.546896	3.566445
C	3.080808	-2.237356	-0.161292
H	4.054106	-1.765627	-0.060655
H	3.212730	-3.300794	0.019519
H	2.741763	-2.090235	-1.178060
C	0.560240	-0.877009	4.065933
H	1.253444	-1.350001	4.750859
H	0.506537	0.179532	4.311360
H	-0.424853	-1.304824	4.226570
H	5.173379	-1.283910	-3.521487
C	5.259916	1.011008	-2.069098
H	5.945135	1.257095	-2.878066
H	5.000180	1.933339	-1.558637
H	5.809212	0.385978	-1.366648

MECP2

Cu	0.15400383	-0.18569055	-1.35142687
B	-0.96585390	0.16925036	1.48503080
H	-1.43173554	0.29325410	2.56788655
C	-3.17338170	-1.08551057	0.83693072
C	-3.62913050	-1.66134675	-0.33408376
C	-2.62726183	-1.48324166	-1.28909533
N	-1.62773972	-0.83536935	-0.71109326
N	-1.95686679	-0.59237844	0.57427650
N	0.99981300	-1.00134098	0.43730016
C	2.07222890	-1.68662553	0.79204598
C	2.10274798	-1.76007500	2.18941839
C	0.99482553	-1.08382382	2.65070124
N	0.34322663	-0.63573793	1.56527407
N	-0.30098174	1.67717567	-0.41878604
C	-0.17615392	2.96409760	-0.69279403
C	-0.51404389	3.69170950	0.45265298
C	-0.84887563	2.77298225	1.42452797
N	-0.71191063	1.56009693	0.86603565
O	0.71522040	-1.84662731	-3.62232874
C	1.85483111	-1.09899547	-3.43666356
C	3.07024197	-1.46313029	-3.88627539
C	4.21737497	-0.67943170	-3.55368938
C	4.09887787	0.38209015	-2.61511087
C	2.87419296	0.74395447	-2.14514601
C	1.58809626	0.15715439	-2.67182331
H	0.92429380	-2.64875598	-4.09698200
H	3.19549969	-2.37550633	-4.45430743
H	2.80355566	1.51143765	-1.38811358
H	1.18603999	0.86881517	-3.41387976
Br	-0.51525477	5.55981910	0.61296454
Br	3.40885989	-2.62505458	3.22016192
Br	-5.27161404	-2.51865712	-0.60966023
C	-2.61131675	-1.89980607	-2.71604501
H	-2.84702472	-2.95677664	-2.80156522
H	-3.36384011	-1.35339658	-3.27894059
H	-1.64011779	-1.72627400	-3.16532835
C	-3.83361830	-0.99119506	2.16591185
H	-4.80257508	-1.47312677	2.12559633
H	-3.24040212	-1.47679768	2.93468515
H	-3.97762452	0.04334952	2.46229868
C	0.24523428	3.48704677	-2.01889100
H	-0.45376790	4.24313810	-2.36470259

H	1.22548131	3.95222976	-1.95681379
H	0.29247276	2.69131808	-2.75019363
C	-1.28332799	3.00074409	2.82890168
H	-1.31898106	4.06481460	3.02815185
H	-2.26952965	2.58490059	3.01159732
H	-0.59525426	2.54510688	3.53485705
C	3.03996799	-2.26323899	-0.17553206
H	4.01236202	-1.79135140	-0.06875991
H	3.16849064	-3.32616017	0.01139777
H	2.71189498	-2.11685180	-1.19597666
C	0.54242647	-0.85106619	4.04858151
H	1.23778258	-1.31690142	4.73620342
H	0.49563164	0.20955834	4.27797851
H	-0.44385469	-1.27120222	4.22160831
H	5.18483689	-0.96302889	-3.93244182
C	5.35315866	1.04559024	-2.11558226
H	5.94365716	1.43538395	-2.94295763
H	5.12653480	1.87284571	-1.44968140
H	5.98768874	0.34424869	-1.57511815

S-I6

Cu	-0.374227	-0.034452	1.288195
B	0.615561	0.043724	-1.536819
H	0.992746	0.053237	-2.661234
C	2.097061	-2.079256	-1.326488
C	2.372288	-2.966530	-0.310496
C	1.644352	-2.538711	0.805428
N	0.969037	-1.454304	0.477480
N	1.241857	-1.173084	-0.818574
N	-1.628837	-0.170080	-0.393351
C	-2.896660	-0.353402	-0.710374
C	-3.011741	-0.365277	-2.104134
C	-1.741456	-0.189653	-2.606870
N	-0.925293	-0.075316	-1.544069
N	0.863875	1.575575	0.457543
C	1.424549	2.731448	0.761418
C	1.994513	3.273759	-0.395395
C	1.754173	2.374238	-1.410554
N	1.068159	1.356767	-0.859978
O	-0.198150	-1.831951	4.744062
C	-1.070869	-0.915273	4.271891
C	-2.437148	-1.020915	4.434775
C	-3.272699	-0.007831	3.972563
C	-2.774578	1.112700	3.334540
C	-1.386816	1.202354	3.168887
C	-0.531721	0.204193	3.625251
H	-0.666021	-2.565002	5.136486
H	-2.856948	-1.876499	4.941567
H	-0.969595	2.097993	2.741141
H	0.539380	0.326694	3.613136
Br	2.913915	4.906773	-0.522991
Br	-4.601086	-0.587382	-3.079182
Br	3.499892	-4.466579	-0.385499
C	1.591752	-3.154389	2.156413
H	1.171250	-4.156280	2.105551
H	2.590722	-3.247395	2.574637
H	0.990361	-2.558943	2.831104
C	2.608954	-2.065506	-2.723451
H	3.277927	-2.905109	-2.869780
H	1.800969	-2.143225	-3.444802
H	3.156441	-1.152386	-2.937622
C	1.425617	3.305187	2.134826
H	2.372523	3.794628	2.338774
H	0.644690	4.053290	2.257175
H	1.280189	2.528974	2.877129
C	2.150596	2.448384	-2.842780

H	2.660619	3.386331	-3.027131
H	2.820590	1.637611	-3.113397
H	1.286654	2.395006	-3.498310
C	-3.969006	-0.535739	0.301378
H	-4.694281	0.273243	0.255873
H	-4.508849	-1.461687	0.121458
H	-3.544602	-0.568175	1.296614
C	-1.293227	-0.133441	-4.024502
H	-2.153618	-0.216024	-4.677855
H	-0.785381	0.800571	-4.244970
H	-0.609209	-0.944002	-4.258035
H	-4.336404	-0.103729	4.123523
C	-3.670752	2.210744	2.838713
H	-4.708035	2.015748	3.091492
H	-3.397972	3.170111	3.272377
H	-3.604093	2.313289	1.757948

-OCH3

$C_6H_5OCH_3$

C	-1.679003	1.283584	0.012615
C	-0.292566	1.280546	0.031719
C	0.397865	2.482834	0.012514
C	-0.291341	3.684073	-0.030640
C	-1.677729	3.679367	-0.051937
C	-2.374848	2.480980	-0.030575
H	-2.221123	0.352023	0.034948
H	0.246007	0.347836	0.067466
H	1.475682	2.486691	0.034783
H	0.228689	4.627840	-0.033937
H	-3.452188	2.502431	-0.033859
O	-2.361453	4.863252	-0.060496
C	-2.649704	5.365629	-1.345041
H	-3.187588	6.296206	-1.207075
H	-1.737878	5.558038	-1.909239
H	-3.270473	4.672507	-1.911604

T-I1

Cu	-0.005420	-0.038337	-1.264276
B	-1.592073	-0.166786	1.327557
H	-2.185418	-0.237191	2.352119
C	-3.644535	-1.355283	0.213625
C	-3.916019	-1.768346	-1.074694
C	-2.784505	-1.470725	-1.841970
N	-1.886898	-0.917954	-1.049904
N	-2.402934	-0.842739	0.197539
N	0.603555	-0.944473	0.395578
C	1.690058	-1.614471	0.743673
C	1.543345	-2.003266	2.076187
C	0.315814	-1.530463	2.499422
N	-0.231016	-0.891697	1.456366
N	-0.618040	1.619826	-0.141196
C	-0.592776	2.931674	-0.272020
C	-1.305345	3.489161	0.795148
C	-1.762829	2.437063	1.561845
N	-1.328534	1.313390	0.967410
O	0.240714	0.458457	-2.983594
C	4.631716	-0.355107	-3.255323
C	5.797429	-0.405409	-2.499470
C	5.971433	0.488955	-1.451007
C	4.978640	1.424316	-1.167691
C	3.827660	1.461057	-1.929399
C	3.640690	0.569918	-2.981325
H	4.505862	-1.052750	-4.067901

H	5.139285	2.110522	-0.353191
H	3.068087	2.193148	-1.706040
H	2.728819	0.592485	-3.553831
C	-4.500546	-1.423556	1.428044
H	-4.725111	-0.432148	1.810937
H	-5.435840	-1.912781	1.184746
H	-4.017042	-1.984094	2.222387
C	-2.536359	-1.696304	-3.290147
H	-2.371776	-2.751100	-3.496912
H	-3.393254	-1.378995	-3.876392
H	-1.667746	-1.132903	-3.614218
C	0.101898	3.605623	-1.400210
H	-0.531734	4.374131	-1.832154
H	1.015267	4.089892	-1.062363
H	0.345842	2.885524	-2.174145
C	-2.580701	2.458745	2.804022
H	-2.757852	3.484645	3.103171
H	-3.543228	1.978162	2.653377
H	-2.077947	1.946057	3.618406
C	2.822775	-1.858300	-0.186035
H	2.582036	-1.532289	-1.191276
H	3.708612	-1.316143	0.132376
H	3.072013	-2.915058	-0.211038
C	-0.340724	-1.662609	3.827129
H	-0.541134	-0.689984	4.266121
H	-1.285037	-2.192887	3.748653
H	0.306288	-2.213979	4.498305
Br	2.787089	-2.981158	3.079802
Br	-5.490259	-2.576695	-1.695154
Br	-1.574929	5.318385	1.107951
H	6.552696	-1.133257	-2.737274
O	7.064372	0.530930	-0.654208
C	8.107406	-0.374052	-0.895800
H	8.870938	-0.162204	-0.157849
H	7.778845	-1.406183	-0.781292
H	8.528767	-0.244815	-1.891635

T-TS1

Cu	0.485894	0.427590	-0.921581
B	-1.390803	-0.558356	1.331321
H	-2.064852	-0.902010	2.245341
C	-2.889786	-2.078628	-0.189069
C	-2.877841	-2.359129	-1.538255
C	-1.803277	-1.647130	-2.090141
N	-1.207892	-0.983024	-1.122158
N	-1.861543	-1.239221	0.031971
N	1.031065	-0.647256	0.682005
C	2.170367	-1.200805	1.064146
C	1.943011	-1.863773	2.271477
C	0.608458	-1.686985	2.579715
N	0.082271	-0.944866	1.595710
N	-0.789062	1.598442	0.183012
C	-1.185018	2.858447	0.130323
C	-2.149387	3.054624	1.121704
C	-2.320931	1.840987	1.755561
N	-1.482514	0.977171	1.163406
O	1.019371	0.875260	-2.582464
C	3.273728	1.544251	-3.588559
C	4.469474	1.110968	-3.065153
C	4.804353	1.424178	-1.743517
C	3.933138	2.197846	-0.970483
C	2.739641	2.629273	-1.494515
C	2.320577	2.238533	-2.796354
H	3.022139	1.309084	-4.608688
H	4.235910	2.462738	0.028844
H	2.103738	3.266615	-0.904132

H	1.582702	2.837106	-3.298280
C	-3.814386	-2.560913	0.871814
H	-4.347726	-1.737733	1.338824
H	-4.543761	-3.234744	0.438643
H	-3.279830	-3.093752	1.652964
C	-1.331531	-1.579896	-3.498724
H	-0.988428	-2.554029	-3.838689
H	-2.138202	-1.272420	-4.158794
H	-0.516768	-0.867908	-3.582255
C	-0.666666	3.829793	-0.867978
H	-1.482800	4.422698	-1.268871
H	0.043079	4.520311	-0.418003
H	-0.186020	3.303998	-1.684333
C	-3.237857	1.481195	2.870114
H	-3.742031	2.371663	3.225458
H	-3.992299	0.769249	2.547865
H	-2.696178	1.037700	3.699549
C	3.422812	-1.105044	0.271130
H	3.219301	-0.717396	-0.720057
H	4.148013	-0.451989	0.748431
H	3.878315	-2.086432	0.176404
C	-0.166425	-2.193220	3.743777
H	-0.654518	-1.382426	4.275256
H	-0.934107	-2.895414	3.431709
H	0.500537	-2.701629	4.429321
Br	3.218032	-2.812568	3.265366
Br	-4.062718	-3.475272	-2.473043
Br	-3.034491	4.663939	1.499245
H	5.143627	0.545487	-3.683754
O	5.942100	1.032763	-1.129865
C	6.887972	0.295503	-1.860673
H	7.710372	0.102667	-1.183632
H	6.478066	-0.652820	-2.203669
H	7.255393	0.856233	-2.718126

T-I2

Cu	0.410159	0.306532	-0.966609
B	-1.591232	-0.234171	1.302140
H	-2.330850	-0.384081	2.217541
C	-2.939925	-2.102157	0.056759
C	-2.821301	-2.668181	-1.194157
C	-1.741368	-2.034557	-1.824919
N	-1.245728	-1.140454	-0.994818
N	-1.967736	-1.176570	0.146580
N	0.888590	-0.364605	0.882921
C	2.004659	-0.739464	1.484723
C	1.681116	-1.142662	2.782300
C	0.315516	-0.993914	2.919058
N	-0.138193	-0.519230	1.750057
N	-0.876497	1.643168	-0.208624
C	-1.181314	2.897393	-0.497716
C	-2.197171	3.304601	0.367994
C	-2.484921	2.224749	1.180916
N	-1.666556	1.232296	0.806108
O	1.292563	0.140361	-2.537399
C	3.511483	-0.411895	-3.202543
C	4.778423	-0.336463	-2.707968
C	5.200881	0.799964	-1.990929
C	4.289301	1.859557	-1.756418
C	3.025978	1.816948	-2.235567
C	2.486383	0.672578	-3.043165
H	3.189240	-1.291067	-3.736272
H	4.643229	2.701415	-1.182909
H	2.357204	2.645391	-2.061217
H	2.248296	1.054837	-4.047428
C	-3.912305	-2.396765	1.143200

H	-4.488831	-1.515861	1.410444
H	-4.600252	-3.166792	0.815504
H	-3.410965	-2.748457	2.040534
C	-1.173732	-2.252327	-3.181903
H	-0.868216	-3.287845	-3.307838
H	-1.915497	-2.039226	-3.947667
H	-0.314454	-1.606675	-3.328868
C	-0.507777	3.662976	-1.579292
H	-1.234917	4.237460	-2.144113
H	0.215455	4.363936	-1.169121
H	0.004897	2.991823	-2.259176
C	-3.491065	2.104877	2.269532
H	-3.988767	3.056633	2.409714
H	-4.243076	1.358547	2.030690
H	-3.025245	1.818681	3.207285
C	3.332316	-0.705781	0.820155
H	3.224995	-0.620390	-0.253908
H	3.922885	0.136074	1.172449
H	3.886010	-1.612018	1.045451
C	-0.552358	-1.286551	4.091022
H	-1.104001	-0.405619	4.404342
H	-1.273469	-2.066193	3.863619
H	0.059763	-1.620500	4.919867
Br	2.885506	-1.764555	4.077549
Br	-3.889274	-4.025321	-1.928532
Br	-3.003811	4.995891	0.405645
H	5.453294	-1.161474	-2.861814
O	6.435871	0.970135	-1.468023
C	7.399612	-0.028580	-1.671032
H	8.306146	0.321273	-1.193226
H	7.101045	-0.972790	-1.218251
H	7.593660	-0.188469	-2.730345

MECP1

Cu	0.35384656	0.14756787	-1.11818290
B	-1.60046466	-0.26683984	1.24663186
H	-2.34037377	-0.42942944	2.16274895
C	-2.99029695	-2.12839806	0.04822877
C	-2.91242258	-2.71644226	-1.19560675
C	-1.82951340	-2.11916537	-1.85506678
N	-1.29136177	-1.22889832	-1.05006765
N	-1.99022639	-1.22838932	0.10320380
N	0.88694382	-0.47144087	0.86084235
C	1.97973868	-0.80173473	1.51864214
C	1.63360416	-1.11855658	2.83596019
C	0.26753819	-0.96154659	2.93055612
N	-0.15298228	-0.56754582	1.71481923
N	-0.95039741	1.62966679	-0.26248034
C	-1.25322883	2.88956059	-0.49182382
C	-2.23118721	3.28687332	0.42925000
C	-2.49881286	2.18823663	1.21771268
N	-1.70232044	1.19969169	0.76985013
O	1.31166857	0.48787827	-2.81387778
C	3.54924051	-0.36149369	-3.07548379
C	4.85814382	-0.29215423	-2.71976031
C	5.32552988	0.86872938	-2.06275371
C	4.46818944	1.96213797	-1.79578726
C	3.16545909	1.89369336	-2.15217842
C	2.59439543	0.76343485	-2.92688599
H	3.14980285	-1.24255411	-3.55146652
H	4.88253285	2.80595092	-1.27157953
H	2.48340478	2.69774220	-1.92920727
H	2.85305894	1.20975084	-3.96018561
C	-3.95317697	-2.38272243	1.15370500
H	-4.51783040	-1.48920122	1.40413495
H	-4.65284959	-3.15386926	0.85364323

H	-3.44644420	-2.71494708	2.05532860
C	-1.29420248	-2.36447125	-3.22073402
H	-0.92829454	-3.38391409	-3.32121315
H	-2.06600138	-2.22252716	-3.97292596
H	-0.48095041	-1.67432987	-3.41972129
C	-0.60409088	3.66418230	-1.58329140
H	-1.34447246	4.17538333	-2.19223502
H	0.06519142	4.42497101	-1.18498325
H	-0.04090427	2.98698586	-2.21761152
C	-3.45971161	2.04781489	2.34519811
H	-3.96002460	2.99401465	2.51482487
H	-4.21541120	1.29681861	2.13276968
H	-2.95710912	1.75786457	3.26345926
C	3.32011959	-0.80858357	0.87686586
H	3.20879042	-0.66622816	-0.19176943
H	3.95241793	-0.01557566	1.27112515
H	3.83109189	-1.75162983	1.05240799
C	-0.62430667	-1.17244695	4.10284946
H	-1.15366416	-0.26233879	4.36900346
H	-1.36752114	-1.93884994	3.90369763
H	-0.03300050	-1.48491908	4.95550166
Br	2.81720199	-1.65529425	4.19474610
Br	-4.03226807	-4.05830662	-1.88889624
Br	-3.01658089	4.99078282	0.55041579
H	5.51930305	-1.12059136	-2.89738888
O	6.56325658	1.02243986	-1.65805039
C	7.51538921	-0.02274678	-1.79768704
H	8.43087661	0.36148459	-1.37153917
H	7.19824780	-0.90499157	-1.25093889
H	7.67109230	-0.26414249	-2.84441063

S-I3

Cu	0.514675	0.000588	-0.884689
B	-1.881708	-0.001975	0.938957
H	-2.828630	-0.003038	1.655834
C	-3.627912	0.003838	-1.000924
C	-3.527830	0.006525	-2.375163
C	-2.160656	0.005284	-2.677804
N	-1.482788	0.002028	-1.549280
N	-2.367291	0.001120	-0.529077
N	0.089792	-1.523484	0.532579
C	0.574846	-2.673362	0.954895
C	-0.270086	-3.176091	1.950817
C	-1.291954	-2.263181	2.097516
N	-1.043502	-1.272050	1.222109
N	0.089980	1.520587	0.537393
C	0.575912	2.668381	0.964331
C	-0.267667	3.166880	1.963522
C	-1.289668	2.253656	2.107300
N	-1.042600	1.266426	1.227097
O	2.144526	0.003307	-2.140008
C	4.307259	0.004464	-3.081724
C	5.762858	0.004636	-2.752507
C	6.178965	0.003055	-1.483853
C	5.222095	0.001230	-0.387344
C	3.896306	0.001143	-0.574961
C	3.348710	0.002973	-1.919823
H	4.053163	-0.858617	-3.698963
H	5.640248	-0.000031	0.606589
H	3.199639	-0.000122	0.246615
H	4.052490	0.868403	-3.697443
C	-4.845057	0.003711	-0.145369
H	-4.880438	0.879142	0.496537
H	-5.728145	0.006332	-0.773236
H	-4.882941	-0.874403	0.492721
C	-1.490649	0.007043	-4.004846

H	-1.772738	-0.867527	-4.585617
H	-1.771105	0.884216	-4.582464
H	-0.414099	0.005792	-3.872881
C	1.821299	3.262725	0.410660
H	1.639997	4.267556	0.038680
H	2.596340	3.333943	1.170125
H	2.188911	2.653222	-0.407357
C	-2.460116	2.288800	3.025060
H	-2.414666	3.183522	3.634570
H	-3.396451	2.298358	2.474898
H	-2.474441	1.426403	3.684949
C	1.820750	-3.265589	0.400131
H	2.189870	-2.652288	-0.414363
H	2.594624	-3.340853	1.160391
H	1.639543	-4.268514	0.022995
C	-2.463547	-2.302090	3.013670
H	-2.478093	-1.443005	3.677867
H	-3.399197	-2.308216	2.462301
H	-2.419463	-3.199890	3.618737
Br	-0.038641	-4.786582	2.890403
Br	-4.937383	0.011016	-3.617479
Br	-0.034643	4.773148	2.909917
H	6.453211	0.006013	-3.577078
O	7.452443	0.002947	-1.044591
C	8.475108	0.004786	-2.007896
H	9.409507	0.004511	-1.462518
H	8.422688	-0.881598	-2.637814
H	8.421783	0.892840	-2.635381

$C_6H_5(OCH_3)O$

C	2.744087	0.260658	-2.132640
C	3.834127	0.298047	-3.187184
C	5.240660	0.121043	-2.712892
C	5.512060	-0.060682	-1.419782
C	4.448171	-0.093273	-0.422884
C	3.160481	0.054547	-0.740899
O	6.730443	-0.232204	-0.861418
C	7.843524	-0.222024	-1.714683
O	1.582642	0.396359	-2.438366
H	3.580890	-0.463394	-3.925835
H	4.760537	-0.245062	0.598037
H	2.379379	0.029511	0.000023
H	3.725249	1.245700	-3.715986
H	6.018876	0.146508	-3.455326
H	8.713298	-0.372826	-1.088275
H	7.784216	-1.022905	-2.450233
H	7.933208	0.730464	-2.234962

$C_6H_4(OCH_3)OH$

C	-1.680554	1.279775	0.013062
C	-0.294540	1.279900	0.036433
C	0.398918	2.479958	0.013541
C	-0.292543	3.679062	-0.029580
C	-1.678842	3.678401	-0.049396
C	-2.372901	2.478349	-0.030176
H	-2.203359	0.337762	0.023959
H	0.231224	4.620648	-0.036733
H	-3.450213	2.495912	-0.037791
O	-2.362468	4.862664	-0.057076
C	-2.650785	5.365499	-1.341639
H	-3.188523	6.296156	-1.203376
H	-1.739000	5.557819	-1.905880
H	-3.271621	4.672606	-1.908353
H	1.476107	2.461469	0.024748

O	0.392016	0.090085	0.038392
H	0.553832	-0.185346	0.937211

T-TS2

Cu	0.332870	0.102024	-0.946206
B	-1.897175	0.023512	1.155580
H	-2.719747	0.025528	2.010215
C	-3.760812	-0.829036	-0.477304
C	-3.759846	-1.144379	-1.819349
C	-2.460916	-0.909375	-2.290283
N	-1.731101	-0.475561	-1.283206
N	-2.512458	-0.425300	-0.181950
N	0.348939	-1.045675	0.745336
C	1.150216	-1.958098	1.266651
C	0.557208	-2.438110	2.437275
C	-0.640360	-1.768329	2.582742
N	-0.739856	-0.928387	1.541227
N	-0.330297	1.667433	0.090013
C	-0.038674	2.957153	0.084546
C	-0.863625	3.587250	1.017002
C	-1.663034	2.604767	1.570893
N	-1.314169	1.450367	0.987149
O	1.260592	-0.242808	-2.465733
C	3.692345	-0.131065	-2.139015
C	4.291022	0.908597	-1.469676
C	5.672294	0.899322	-1.287770
C	6.421911	-0.158441	-1.786122
C	5.794031	-1.203332	-2.462786
C	4.423625	-1.191920	-2.640837
H	2.385159	-0.146896	-2.371231
H	3.711581	1.732752	-1.086190
H	6.141242	1.713954	-0.765067
H	3.935098	-1.997054	-3.164618
C	-1.901362	-1.083651	-3.656768
H	-2.390287	-0.415379	-4.361274
H	-0.836247	-0.876778	-3.653574
H	-2.062280	-2.099010	-4.008817
C	-4.870034	-0.894683	0.511606
H	-5.775776	-1.227676	0.019375
H	-4.644996	-1.589807	1.315570
H	-5.061726	0.076682	0.958101
C	1.002117	3.542253	-0.800966
H	0.597493	4.378820	-1.362797
H	1.839985	3.916986	-0.218313
H	1.367592	2.802528	-1.505556
C	-2.724214	2.722784	2.606006
H	-2.788711	3.749764	2.944119
H	-3.693170	2.431699	2.211245
H	-2.511130	2.091193	3.462797
C	2.443276	-2.350213	0.649737
H	2.574026	-1.869766	-0.311684
H	3.276105	-2.073245	1.290834
H	2.484193	-3.426721	0.510467
C	-1.669951	-1.898733	3.648208
H	-1.841079	-0.951927	4.151303
H	-2.619222	-2.234721	3.241444
H	-1.339722	-2.622369	4.383420
Br	1.271889	-3.741694	3.579188
Br	-5.208256	-1.767035	-2.836353
Br	-0.869420	5.416463	1.423123
O	7.765104	-0.264603	-1.665495
C	8.463815	0.753526	-1.001561
H	8.142974	0.851009	0.034689
H	9.508376	0.469419	-1.023361
H	8.345465	1.713732	-1.501653
H	6.402827	-2.008204	-2.839455

T-I4

Cu	-0.029267	0.183621	-1.158900
B	-2.076948	-0.091628	1.102575
H	-2.892298	-0.157540	1.961790
C	-1.913129	-2.703517	1.222167
C	-1.180672	-3.633880	0.515398
C	-0.434363	-2.925574	-0.435327
N	-0.712359	-1.644534	-0.302327
N	-1.607519	-1.502839	0.700211
N	0.162620	1.004791	0.748225
C	1.076106	1.687030	1.413625
C	0.613151	1.877935	2.719057
C	-0.621953	1.267843	2.797150
N	-0.869334	0.747108	1.584771
N	-1.915164	0.808996	-1.232116
C	-2.647598	1.395143	-2.164013
C	-3.932154	1.574408	-1.649529
C	-3.923074	1.061521	-0.365677
N	-2.683499	0.605017	-0.142365
O	1.468301	-0.155982	-2.108278
C	4.655110	0.986609	-2.056987
C	5.737570	1.817668	-2.090949
C	6.989533	1.255884	-1.832005
C	7.088554	-0.102631	-1.556079
C	5.948022	-0.906076	-1.535506
C	4.700071	-0.361544	-1.790510
H	2.068254	0.570719	-2.249645
H	5.650569	2.869636	-2.308891
H	7.862502	1.883955	-1.851227
H	3.804566	-0.960655	-1.779160
C	0.523220	-3.424661	-1.457312
H	0.007617	-4.009824	-2.215102
H	1.026695	-2.589825	-1.933255
H	1.265411	-4.072187	-0.998801
C	-2.865070	-2.903829	2.347432
H	-2.952057	-3.961489	2.564746
H	-2.526977	-2.398707	3.247787
H	-3.852614	-2.522469	2.104854
C	-2.106288	1.761493	-3.499124
H	-2.713024	1.329180	-4.289336
H	-2.112068	2.839621	-3.635548
H	-1.088402	1.403431	-3.613837
C	-5.024597	0.988771	0.630646
H	-5.920612	1.432319	0.214006
H	-5.244561	-0.040117	0.899153
H	-4.768416	1.522170	1.540942
C	2.350270	2.139364	0.796767
H	2.538226	1.606342	-0.127454
H	2.324091	3.206486	0.588313
H	3.183299	1.959173	1.468696
C	-1.549655	1.159624	3.954831
H	-2.519847	1.590288	3.726515
H	-1.705891	0.123608	4.241085
H	-1.132182	1.687230	4.803747
Br	1.510258	2.793995	4.087545
Br	-1.173716	-5.493338	0.763758
Br	-5.376390	2.363076	-2.545583
O	8.253614	-0.741366	-1.293512
C	9.443301	-0.000963	-1.302053
H	9.434067	0.784047	-0.546923
H	10.238437	-0.700101	-1.075206
H	9.630920	0.447116	-2.276983
H	6.065813	-1.954622	-1.317716

T-TS3

Cu	0.231163	0.094839	-1.356293
B	-1.100229	-0.122688	1.422848
H	-1.629723	-0.146830	2.483167
C	-3.424384	-0.786524	0.441921
C	-3.903913	-0.943127	-0.841171
C	-2.839159	-0.678127	-1.709322
N	-1.777994	-0.381252	-0.977946
N	-2.131937	-0.443330	0.325123
N	0.855983	-1.226998	0.313508
C	1.756627	-2.161159	0.562831
C	1.522127	-2.677220	1.842149
C	0.433128	-2.003487	2.348393
N	0.053348	-1.132627	1.398466
N	-0.026122	1.645210	-0.039369
C	0.200979	2.953816	-0.023888
C	-0.162654	3.445979	1.229868
C	-0.637557	2.373516	1.957581
N	-0.547919	1.301203	1.161995
O	0.342744	-0.981724	-2.943200
C	1.807534	-0.153339	-3.538448
C	2.886621	-1.033174	-3.762295
C	4.107456	-0.767834	-3.204663
C	4.297629	0.328069	-2.336010
C	3.258544	1.191891	-2.105716
C	1.994510	0.967475	-2.709775
H	0.514065	-1.912624	-2.793672
H	2.770029	-1.881133	-4.420176
H	3.398566	2.065663	-1.492198
H	1.317484	1.805406	-2.797101
Br	-0.043738	5.224533	1.813175
Br	2.515900	-4.023992	2.689184
Br	-5.648988	-1.411216	-1.344646
C	-2.837456	-0.681005	-3.196555
H	-3.374060	-1.547763	-3.570788
H	-3.345925	0.201633	-3.578777
H	-1.822005	-0.701699	-3.571087
C	-4.130239	-0.951150	1.740914
H	-5.159437	-1.236124	1.559542
H	-3.665498	-1.721250	2.349497
H	-4.127035	-0.028811	2.314111
C	0.707295	3.739978	-1.182238
H	0.569505	4.796766	-0.987601
H	1.764389	3.571812	-1.359542
H	0.165063	3.493742	-2.089095
C	-1.166587	2.342617	3.347678
H	-1.044533	3.318312	3.801849
H	-2.222351	2.088668	3.364045
H	-0.639901	1.612886	3.954033
C	2.821628	-2.548250	-0.395752
H	3.801280	-2.262183	-0.021505
H	2.832069	-3.625306	-0.539801
H	2.676842	-2.065362	-1.351987
C	-0.241440	-2.154671	3.665695
H	0.260248	-2.922737	4.241830
H	-0.215519	-1.229950	4.234601
H	-1.282222	-2.442073	3.548993
O	5.550276	0.434224	-1.810675
C	5.821055	1.529300	-0.984693
H	6.855088	1.435666	-0.676148
H	5.692175	2.473995	-1.513520
H	5.184656	1.533464	-0.099364
H	4.960938	-1.394348	-3.411430

T-I5

Cu	0.112852	-0.252555	-1.300871
B	-1.073381	0.264066	1.492687

H	-1.560630	0.436233	2.560023
C	-3.402423	-0.679549	0.751191
C	-3.881992	-1.198466	-0.436044
C	-2.824314	-1.170005	-1.346690
N	-1.770698	-0.660117	-0.729477
N	-2.117857	-0.362355	0.539995
N	0.777490	-1.131983	0.509434
C	1.736082	-1.954475	0.896759
C	1.697881	-2.045854	2.292147
C	0.665142	-1.238725	2.717783
N	0.123711	-0.698145	1.614158
N	-0.129844	1.690691	-0.354412
C	0.150267	2.957172	-0.606324
C	-0.164590	3.714577	0.526442
C	-0.652872	2.835558	1.468933
N	-0.620552	1.617021	0.904821
O	0.450098	-1.739085	-4.043676
C	1.643053	-1.320211	-3.526048
C	2.805158	-1.991435	-3.712068
C	4.014968	-1.501908	-3.163353
C	3.994718	-0.313137	-2.400482
C	2.839116	0.381051	-2.197777
C	1.532628	-0.083096	-2.737073
H	0.589562	-2.513346	-4.584436
H	2.815420	-2.909417	-4.283002
H	2.838851	1.290371	-1.623604
H	1.076688	0.684102	-3.376194
Br	0.037946	5.570839	0.704183
Br	2.838867	-3.080841	3.361932
Br	-5.613883	-1.825906	-0.780627
C	-2.801758	-1.604126	-2.768208
H	-3.163177	-2.624825	-2.857077
H	-3.455789	-0.976448	-3.368298
H	-1.799001	-1.554868	-3.178252
C	-4.104067	-0.479908	2.047084
H	-5.128518	-0.820854	1.960897
H	-3.623128	-1.036717	2.845545
H	-4.114839	0.567804	2.332288
C	0.694807	3.437639	-1.903725
H	0.074531	4.235474	-2.302375
H	1.696936	3.839802	-1.779073
H	0.734965	2.634295	-2.627118
C	-1.137685	3.107096	2.848907
H	-1.050086	4.165859	3.060451
H	-2.178139	2.819937	2.968234
H	-0.557315	2.562857	3.587983
C	2.656024	-2.637405	-0.047054
H	3.661971	-2.234991	0.032049
H	2.707158	-3.698446	0.180536
H	2.327395	-2.510057	-1.070020
C	0.186786	-0.970807	4.100643
H	0.793579	-1.525364	4.806058
H	0.256959	0.084547	4.347181
H	-0.848428	-1.272746	4.228636
O	5.211353	0.044471	-1.900698
C	5.301508	1.233612	-1.170999
H	6.340682	1.341089	-0.883577
H	5.006276	2.095856	-1.768732
H	4.684847	1.203972	-0.272819
H	4.946928	-2.018058	-3.306473

MECP2

Cu	0.05529516	-0.23519133	-1.32719041
B	-1.10659099	0.26226494	1.47603398
H	-1.58880299	0.44078445	2.54425231
C	-3.43680580	-0.68598543	0.74575988

C	-3.92474141	-1.19487078	-0.44301370
C	-2.87684234	-1.14853845	-1.36317530
N	-1.82022489	-0.63819040	-0.74914215
N	-2.15784183	-0.35702380	0.52657361
N	0.72065551	-1.15501265	0.48178954
C	1.68724016	-1.97005809	0.86552336
C	1.66810599	-2.04466992	2.26275302
C	0.64066991	-1.23420874	2.69351533
N	0.08240969	-0.70830664	1.59122906
N	-0.17034163	1.67367621	-0.38656468
C	0.13570244	2.93372750	-0.64371850
C	-0.15353640	3.69701471	0.49150770
C	-0.65078870	2.82881735	1.43983717
N	-0.65008427	1.60968807	0.87777552
O	0.45429832	-1.92483731	-3.62389672
C	1.68100103	-1.35974641	-3.36858962
C	2.84972542	-1.89965122	-3.76473109
C	4.07973821	-1.29015920	-3.38631303
C	4.05789276	-0.21102118	-2.46096900
C	2.89415238	0.33710869	-2.02569776
C	1.56313842	-0.08104041	-2.60469997
H	0.56511786	-2.73277450	-4.12090174
H	2.86648883	-2.82058075	-4.33168049
H	2.89401964	1.11075708	-1.27788604
H	1.27460405	0.67151892	-3.35890023
Br	0.08838977	5.54808551	0.66646209
Br	2.82661632	-3.06330341	3.32877057
Br	-5.65480928	-1.83032304	-0.77809058
C	-2.87005108	-1.56437276	-2.79033050
H	-3.23709355	-2.58218050	-2.88736432
H	-3.52816479	-0.92678031	-3.37520109
H	-1.87111449	-1.51748503	-3.20867345
C	-4.12579869	-0.50694002	2.05126587
H	-5.14829874	-0.85459434	1.97112541
H	-3.63166234	-1.07017470	2.83706979
H	-4.14007475	0.53726546	2.34889377
C	0.67932178	3.39995636	-1.94607933
H	0.06493254	4.20174417	-2.34617442
H	1.68580867	3.79148311	-1.82573300
H	0.71158305	2.59020611	-2.66223961
C	-1.11383456	3.10916667	2.82529776
H	-1.01466163	4.16777069	3.03207338
H	-2.15440910	2.82990225	2.96040651
H	-0.52624699	2.56261230	3.55694459
C	2.60137436	-2.66368763	-0.07673216
H	3.60729887	-2.26144295	-0.00248332
H	2.65002004	-3.72264548	0.16064823
H	2.27419275	-2.54175048	-1.10079947
C	0.18135006	-0.94819521	4.07915954
H	0.79373410	-1.49794421	4.78331458
H	0.26126841	0.10953168	4.31253757
H	-0.85404490	-1.24223131	4.22355328
O	5.30011424	0.15583782	-2.03164437
C	5.39965364	1.23300361	-1.14811453
H	6.45715514	1.39732357	-0.97738743
H	4.96058630	2.13834494	-1.56717055
H	4.91552235	1.02135274	-0.19445346
H	5.02592427	-1.69326580	-3.69800740

S-I6

Cu	-0.300724	-0.196416	1.239492
B	0.712491	0.223909	-1.549517
H	1.112009	0.357877	-2.658302
C	2.818455	-1.266238	-1.241190
C	3.346791	-1.984197	-0.191795
C	2.485034	-1.801094	0.895476

N	1.494349	-1.016359	0.518989
N	1.693662	-0.689579	-0.779715
N	-1.339070	-0.799602	-0.487996
C	-2.452292	-1.411419	-0.847656
C	-2.523595	-1.419633	-2.244764
C	-1.385691	-0.791135	-2.702908
N	-0.687675	-0.428228	-1.612854
N	0.280727	1.761978	0.408382
C	0.385250	3.042877	0.710942
C	0.815280	3.740410	-0.422840
C	0.973996	2.805410	-1.422101
N	0.642331	1.617681	-0.885143
O	0.428176	-1.653928	4.851604
C	-0.701608	-1.196906	4.260093
C	-1.915832	-1.857479	4.335321
C	-3.046589	-1.317715	3.743160
C	-2.983100	-0.114274	3.060296
C	-1.762013	0.559067	2.979811
C	-0.621614	0.016276	3.579070
H	0.268869	-2.500779	5.260346
H	-1.990314	-2.791513	4.871064
H	-1.707322	1.536248	2.538806
H	0.298888	0.574657	3.625184
Br	1.119994	5.589983	-0.542805
Br	-3.903290	-2.156946	-3.284333
Br	4.916001	-3.015870	-0.197111
C	2.600372	-2.366298	2.264819
H	2.520899	-3.450917	2.240529
H	3.567366	-2.125510	2.698605
H	1.826149	-1.975719	2.913033
C	3.336162	-1.114644	-2.627793
H	4.251429	-1.684517	-2.734308
H	2.621896	-1.476194	-3.361511
H	3.552316	-0.076179	-2.860877
C	0.087393	3.591851	2.062259
H	0.784521	4.385918	2.309573
H	-0.913151	4.017718	2.111315
H	0.172279	2.821401	2.819742
C	1.424276	3.001856	-2.826598
H	1.591668	4.057040	-3.006784
H	2.350876	2.471576	-3.025837
H	0.682760	2.647820	-3.536296
C	-3.409660	-1.988285	0.132986
H	-4.209449	-1.292952	0.373888
H	-3.863019	-2.888335	-0.269473
H	-2.896266	-2.237743	1.054035
C	-0.951422	-0.539319	-4.103614
H	-1.715766	-0.889859	-4.786810
H	-0.789576	0.518709	-4.285994
H	-0.025577	-1.058801	-4.332849
O	-4.134035	0.333552	2.512902
C	-4.110740	1.537195	1.784329
H	-5.114704	1.679006	1.405922
H	-3.849840	2.382026	2.420030
H	-3.415027	1.483052	0.949686
H	-3.994514	-1.824406	3.806828

$-N(CH_3)_2$

$C_6H_5N(CH3)_2$

C	-1.408122	1.364741	0.000763
C	-0.036364	1.464445	-0.163360
C	0.598214	2.693075	-0.165820
C	-0.132424	3.879344	-0.001405
C	-1.521098	3.768520	0.164126
C	-2.137743	2.530791	0.163793

H	-1.895405	0.404229	0.001592
H	0.557109	0.572999	-0.292429
H	1.664719	2.726339	-0.296181
H	-2.124067	4.648959	0.293696
H	-3.207522	2.483362	0.293671
N	0.490954	5.108120	-0.002462
C	-0.284253	6.308358	0.168250
H	-1.026436	6.432723	-0.621390
H	-0.809712	6.324463	1.124006
H	0.374943	7.166833	0.139643
C	1.917216	5.191223	-0.174309
H	2.456484	4.666838	0.615654
H	2.239878	4.775429	-1.129736
H	2.220646	6.230232	-0.147318

T-I1

Cu	-1.021559	0.001491	1.754770
B	-1.505959	-0.006185	-1.246225
H	-1.728656	-0.009907	-2.411215
C	-0.241446	2.255607	-1.630094
C	0.416585	3.128196	-0.788376
C	0.278307	2.622082	0.508871
N	-0.432306	1.512778	0.441597
N	-0.747916	1.282560	-0.854168
N	-0.337065	-1.464643	0.432589
C	0.446331	-2.524117	0.492320
C	0.624742	-3.005984	-0.809294
C	-0.086835	-2.171156	-1.645667
N	-0.661311	-1.242605	-0.862587
N	-2.809522	-0.056230	0.893360
C	-4.052009	-0.102117	1.343588
C	-4.906186	-0.130983	0.240676
C	-4.105221	-0.099875	-0.885887
N	-2.837293	-0.054674	-0.455474
O	-0.025850	0.032958	3.260224
C	3.284047	0.097156	2.168539
C	4.508236	0.069686	2.817714
C	5.698200	0.058149	2.112141
C	5.705125	0.083532	0.710664
C	4.463982	0.098666	0.060139
C	3.282775	0.109814	0.782083
H	2.357427	0.101013	2.719162
H	4.543879	0.051330	3.895784
H	6.622436	0.028870	2.660028
H	2.345367	0.123069	0.248628
C	0.814409	3.151890	1.789463
H	0.451762	4.159262	1.974558
H	0.524475	2.510834	2.614496
H	1.899563	3.188822	1.757379
C	-0.401549	2.308673	-3.107923
H	0.062529	3.209746	-3.489961
H	0.067377	1.454854	-3.588637
H	-1.449248	2.315337	-3.393065
C	-4.381571	-0.117013	2.792944
H	-5.001995	0.735434	3.054744
H	-4.934517	-1.015468	3.052161
H	-3.479237	-0.083213	3.394830
C	-4.492126	-0.111315	-2.321631
H	-5.571227	-0.149311	-2.406703
H	-4.138975	0.779911	-2.831463
H	-4.077046	-0.973955	-2.834029
C	1.010988	-3.030309	1.770115
H	0.653997	-2.435058	2.603111
H	2.095354	-2.968287	1.753737
C	-0.236942	-2.220079	-3.124721
H	-1.280631	-2.294379	-3.414899

H	0.175335	-1.331911	-3.594998
H	0.288297	-3.084010	-3.513315
Br	1.641216	-4.502189	-1.301989
Br	1.328053	4.693870	-1.269873
Br	-6.777274	-0.198278	0.300754
H	4.412478	0.102564	-1.013408
N	6.895137	0.105830	-0.005061
C	8.121841	-0.202260	0.687502
H	8.310964	0.515998	1.480655
H	8.127139	-1.202442	1.129977
H	8.948570	-0.135378	-0.009637
C	6.853498	-0.174041	-1.418991
H	6.443470	-1.161373	-1.649817
H	6.257989	0.567441	-1.944915
H	7.858575	-0.121657	-1.820266

T-TS1

Cu	0.359309	0.348542	-0.916801
B	-1.634982	-0.451479	1.317327
H	-2.348994	-0.721874	2.225948
C	-3.247050	-1.842354	-0.211505
C	-3.238554	-2.138127	-1.557312
C	-2.092150	-1.536568	-2.096894
N	-1.453989	-0.919802	-1.125242
N	-2.147872	-1.100837	0.019121
N	0.782690	-0.748771	0.711266
C	1.863850	-1.394302	1.114728
C	1.559052	-2.034387	2.317765
C	0.238469	-1.746667	2.600560
N	-0.204962	-0.964388	1.606472
N	-0.821326	1.628537	0.158445
C	-1.083489	2.922514	0.099257
C	-2.034173	3.219553	1.078648
C	-2.336544	2.031731	1.713023
N	-1.584694	1.084728	1.132298
O	1.005972	0.663867	-2.559662
C	3.328447	1.115183	-3.564688
C	4.491432	0.636026	-3.027467
C	4.888743	0.974930	-1.713052
C	4.059428	1.856022	-0.995298
C	2.890043	2.335683	-1.539934
C	2.429016	1.917154	-2.812316
H	3.059770	0.846457	-4.572369
H	4.343427	2.189524	-0.013635
H	2.309912	3.047540	-0.976891
H	1.732109	2.541492	-3.340532
C	-4.230113	-2.223525	0.837853
H	-4.692647	-1.348688	1.286195
H	-5.011493	-2.832432	0.399196
H	-3.760709	-2.793673	1.634436
C	-1.590873	-1.530510	-3.496851
H	-1.349194	-2.538502	-3.825055
H	-2.347417	-1.142411	-4.173773
H	-0.702291	-0.911418	-3.568059
C	-0.449237	3.831522	-0.890796
H	-1.192632	4.499437	-1.314670
H	0.314458	4.450721	-0.425721
H	-0.001151	3.256341	-1.692431
C	-3.298273	1.770869	2.817334
H	-3.716311	2.708855	3.162096
H	-4.115275	1.135181	2.488256
H	-2.813722	1.280529	3.655695
C	3.134381	-1.402965	0.345399
H	3.010156	-0.910825	-0.611557
H	3.925831	-0.896573	0.890880

H	3.460926	-2.424718	0.173505
C	-0.598320	-2.184695	3.749362
H	-1.027241	-1.335342	4.271633
H	-1.415935	-2.820423	3.421985
H	0.010145	-2.746493	4.447578
Br	2.733647	-3.082310	3.336455
Br	-4.507208	-3.149146	-2.502192
Br	-2.754191	4.912500	1.442126
H	5.115902	0.005496	-3.633755
N	6.041474	0.458036	-1.157073
C	6.560206	1.043394	0.056579
H	6.826563	2.096426	-0.059619
H	5.839556	0.966094	0.866201
H	7.446471	0.500435	0.360636
C	6.974665	-0.253050	-1.998941
H	6.496784	-1.106067	-2.471469
H	7.405456	0.373595	-2.783362
H	7.783057	-0.633586	-1.386823

T-I2

Cu	0.291038	0.277681	-0.941931
B	-1.778145	-0.189268	1.288098
H	-2.538132	-0.313140	2.190839
C	-3.224737	-1.938576	-0.018824
C	-3.121014	-2.485183	-1.279582
C	-1.991005	-1.910519	-1.878802
N	-1.453255	-1.068109	-1.021587
N	-2.195930	-1.080724	0.106824
N	0.693559	-0.469398	0.896130
C	1.773264	-0.937944	1.498105
C	1.406568	-1.352807	2.780527
C	0.052935	-1.113832	2.907144
N	-0.352103	-0.576500	1.747310
N	-0.925324	1.672878	-0.168739
C	-1.152949	2.947442	-0.438562
C	-2.158716	3.396399	0.418600
C	-2.521689	2.320312	1.205743
N	-1.754705	1.289570	0.825236
O	1.174490	0.108170	-2.507944
C	3.363564	-0.540442	-3.178196
C	4.622162	-0.565462	-2.682092
C	5.156584	0.506618	-1.908147
C	4.282818	1.589818	-1.621189
C	3.014772	1.644191	-2.102481
C	2.405741	0.593249	-2.977665
H	2.991421	-1.377954	-3.746137
H	4.625926	2.398500	-0.999502
H	2.398794	2.499320	-1.869747
H	2.196950	1.041654	-3.960872
C	-4.233456	-2.190470	1.045012
H	-4.761799	-1.280616	1.315228
H	-4.959579	-2.913218	0.692899
H	-3.771101	-2.583931	1.945895
C	-1.412982	-2.137472	-3.229917
H	-1.157565	-3.185225	-3.366479
H	-2.130396	-1.876510	-4.003949
H	-0.519724	-1.534318	-3.353450
C	-0.418072	3.692581	-1.494195
H	-1.102430	4.312996	-2.063970
H	0.331965	4.348428	-1.058356
H	0.073274	3.005687	-2.174022
C	-3.552090	2.238498	2.275024
H	-3.984475	3.218223	2.437656
H	-4.350529	1.554801	2.001372
H	-3.123641	1.891412	3.209918
C	3.108261	-0.980264	0.848765

H	3.027248	-0.820272	-0.219103
H	3.762521	-0.215707	1.260096
H	3.578733	-1.943087	1.023791
C	-0.847712	-1.379661	4.060601
H	-1.362468	-0.477898	4.376850
H	-1.600841	-2.121372	3.810468
H	-0.266559	-1.753873	4.894584
Br	2.550607	-2.089954	4.070409
Br	-4.261778	-3.754750	-2.060092
Br	-2.866938	5.130834	0.477000
H	5.227969	-1.433601	-2.874856
N	6.470875	0.498057	-1.471232
C	7.224609	-0.731491	-1.534688
H	6.808313	-1.522797	-0.905505
H	7.276256	-1.102130	-2.553664
H	8.240373	-0.540038	-1.210065
C	6.884155	1.456116	-0.474584
H	6.721977	2.471685	-0.822742
H	6.361714	1.336363	0.478768
H	7.946439	1.343282	-0.294161

MECP1

Cu	0.41182205	0.07154551	-1.02755924
B	-1.72115887	-0.22181403	1.23847275
H	-2.49204778	-0.35621285	2.13315415
C	-3.20695149	-1.94078473	-0.05340473
C	-3.13512204	-2.49099695	-1.31421022
C	-1.98473788	-1.96351160	-1.91864990
N	-1.40276714	-1.15001959	-1.06534531
N	-2.13809710	-1.12968060	0.06395799
N	0.75904777	-0.59296210	0.91266728
C	1.80609318	-1.02412900	1.58935762
C	1.39880140	-1.34580462	2.88668697
C	0.04592275	-1.08768582	2.94955744
N	-0.30915078	-0.63129913	1.73605435
N	-0.90801608	1.65531739	-0.20915146
C	-1.15128252	2.92634109	-0.44427487
C	-2.14031854	3.36391486	0.44682215
C	-2.48086565	2.27711786	1.22154538
N	-1.71203747	1.25664585	0.79561009
O	1.13466469	0.45568404	-2.79407612
C	3.30305049	-0.46660300	-3.15423908
C	4.53957257	-0.56664008	-2.63785131
C	5.08289346	0.50767006	-1.84922879
C	4.26028276	1.65282192	-1.57689583
C	3.02639792	1.74236377	-2.10316549
C	2.42913207	0.74029557	-3.03488160
H	2.87264392	-1.28551745	-3.70913961
H	4.62137441	2.41999786	-0.91820324
H	2.39728302	2.58539712	-1.86709194
H	2.56804346	1.23147579	-4.03418423
C	-4.22127511	-2.15057413	1.01505673
H	-4.73178479	-1.22535394	1.26743588
H	-4.96175655	-2.86630595	0.67738435
H	-3.76632088	-2.53328265	1.92424900
C	-1.42692914	-2.20174036	-3.27711528
H	-1.11831910	-3.23788884	-3.39868466
H	-2.17080868	-1.99498784	-4.04255210
H	-0.57194021	-1.55227827	-3.43491326
C	-0.44462770	3.67647932	-1.51726056
H	-1.14485019	4.26424626	-2.10427214
H	0.28628303	4.36959392	-1.10333846
H	0.05561566	2.97325197	-2.17474376
C	-3.48394920	2.17581020	2.31567488
H	-3.93360851	3.14694326	2.48717485
H	-4.27542350	1.47446634	2.06707364

H	-3.02844500	1.84183644	3.24327144
C	3.16054780	-1.12206389	0.98659767
H	3.09409948	-0.96430600	-0.08318400
H	3.83437460	-0.37935685	1.41035967
H	3.59734988	-2.09987073	1.17214341
C	-0.89101772	-1.26313043	4.09198617
H	-1.36563812	-0.32508512	4.36417230
H	-1.67761869	-1.97307170	3.85383149
H	-0.34641344	-1.63320988	4.95299162
Br	2.50026272	-2.00475761	4.26063170
Br	-4.33615290	-3.71304670	-2.08929929
Br	-2.85388488	5.10084289	0.54887458
H	5.11000303	-1.46704253	-2.77078271
N	6.31362215	0.42939052	-1.35529626
C	7.15307473	-0.73882323	-1.59755204
H	6.73867025	-1.62782587	-1.13021977
H	7.26882091	-0.91849051	-2.66166874
H	8.13198790	-0.55890683	-1.17684903
C	6.84634858	1.48876474	-0.50490837
H	6.83848057	2.44228197	-1.02404472
H	6.27308997	1.58014425	0.41304523
H	7.86769641	1.25067260	-0.24574064

S-I3

Cu	0.084956	0.000216	1.208574
B	-1.848281	-0.000326	-1.114377
H	-2.625694	-0.000533	-2.012028
C	-3.960957	-0.004571	0.415625
C	-4.155865	-0.005046	1.779553
C	-2.884975	-0.002460	2.366614
N	-1.982061	-0.000524	1.407965
N	-2.629124	-0.001772	0.221704
N	-0.021558	1.532717	-0.296400
C	0.534770	2.686687	-0.604391
C	-0.070016	3.178481	-1.766981
C	-1.026920	2.256566	-2.132812
N	-0.971201	1.270415	-1.218945
N	-0.020797	-1.532502	-0.296450
C	0.538579	-2.684400	-0.606639
C	-0.062690	-3.174008	-1.771977
C	-1.020589	-2.252907	-2.137249
N	-0.968882	-1.269351	-1.220346
O	1.689723	0.000280	2.497125
C	3.903079	0.000373	3.324835
C	5.337266	0.000542	2.919715
C	5.727734	0.000697	1.628855
C	4.688859	0.000718	0.599486
C	3.373017	0.000598	0.858466
C	2.885648	0.000435	2.222071
H	3.679115	0.862732	3.956142
H	4.994876	0.000835	-0.432219
H	2.640777	0.000618	0.068227
H	3.679263	-0.862210	3.955893
C	-4.969239	-0.006713	-0.678243
H	-4.867865	-0.883503	-1.311126
H	-5.965024	-0.008606	-0.251175
H	-4.871314	0.870223	-1.311457
C	-2.515425	-0.001801	3.806365
H	-2.916021	0.873278	4.311653
H	-2.912414	-0.878597	4.311522
H	-1.435742	0.000410	3.909701
C	1.620113	-3.291067	0.214493
H	1.382732	-4.321030	0.465803
H	2.567894	-3.298203	-0.319143
H	1.742592	-2.730397	1.134320
C	-1.957973	-2.277830	-3.292421

H	-1.779652	-3.168370	-3.883382
H	-2.993295	-2.288174	-2.964369
H	-1.824211	-1.410698	-3.932370
C	1.617028	3.293051	0.216012
H	1.741731	2.730844	1.134600
H	2.563888	3.302190	-0.319234
H	1.378944	4.322289	0.469588
C	-1.967071	2.283151	-3.285698
H	-1.834212	1.417483	-3.927793
H	-3.001616	2.292022	-2.955123
H	-1.790902	3.175101	-3.875176
Br	0.351394	4.787554	-2.641401
Br	-5.797520	-0.008414	2.692954
Br	0.363696	-4.779806	-2.650000
H	6.054860	0.000516	3.719562
N	7.046292	0.000824	1.224879
C	8.063334	0.000814	2.241541
H	7.991915	0.881813	2.881473
H	7.992044	-0.880306	2.881320
H	9.041435	0.000925	1.778569
C	7.420906	0.001013	-0.169336
H	7.064821	-0.882223	-0.699482
H	7.064679	0.884311	-0.699284
H	8.500927	0.001107	-0.237290

$C_6H_5N(CH3)_2O$

O	1.697106	0.001765	2.467080
C	3.904118	0.004325	3.352650
C	5.343496	0.004909	2.955606
C	5.745256	0.003589	1.670648
C	4.721302	0.001517	0.620733
C	3.408498	0.000894	0.869593
C	2.884843	0.002269	2.236643
H	3.677369	0.868049	3.980308
H	5.044849	0.000446	-0.405738
H	2.682574	-0.000627	0.073678
H	3.678636	-0.858280	3.982304
H	6.057887	0.006441	3.759002
N	7.072842	0.004059	1.282510
C	8.076363	0.006139	2.310836
H	7.996124	0.887272	2.950036
H	7.997450	-0.873484	2.952272
H	9.060836	0.006306	1.860665
C	7.466030	0.002827	-0.104609
H	7.117830	-0.880644	-0.640529
H	7.116547	0.884608	-0.642481
H	8.547249	0.003547	-0.158573

$C_6H_4N(CH3)_2OH$

C	-1.503836	1.417445	-0.122202
C	-0.132079	1.476704	-0.290238
C	0.539149	2.687564	-0.233872
C	-0.144957	3.885634	-0.006104
C	-1.534688	3.804884	0.161715
C	-2.198016	2.594782	0.104417
H	0.430367	0.571682	-0.468111
H	1.605412	2.686058	-0.370078
H	-2.111648	4.694358	0.339638
H	-3.266335	2.551817	0.235659
N	0.518765	5.097426	0.051327
O	-2.211486	0.255146	-0.168217
H	-1.618456	-0.473150	-0.329461
C	1.944095	5.139923	-0.125326
H	2.470417	4.557974	0.633481
H	2.249168	4.761721	-1.102753

H	2.284081	6.165120	-0.048343
C	-0.218871	6.308259	0.287359
H	-0.961435	6.496342	-0.490000
H	-0.739833	6.292928	1.246231
H	0.465690	7.147214	0.298270

T-TS2

Cu	0.188200	0.108877	-0.886121
B	-2.162996	0.017589	1.082227
H	-3.035860	0.018585	1.885722
C	-3.897186	-0.943962	-0.631169
C	-3.804699	-1.297505	-1.960420
C	-2.488144	-1.030899	-2.360500
N	-1.835547	-0.543050	-1.325637
N	-2.683029	-0.488357	-0.275046
N	0.136055	-0.989742	0.840955
C	0.930947	-1.860429	1.437688
C	0.280547	-2.327396	2.583077
C	-0.943760	-1.693428	2.634625
N	-1.003588	-0.886173	1.564601
N	-0.583033	1.679841	0.064438
C	-0.328167	2.977034	0.041125
C	-1.226691	3.606117	0.903768
C	-2.030415	2.614808	1.435091
N	-1.613565	1.456665	0.905903
O	1.205120	-0.262384	-2.339000
C	3.609494	-0.071318	-1.873158
C	4.151810	1.028353	-1.246950
C	5.513720	1.066813	-0.989423
C	6.348609	-0.001107	-1.350134
C	5.761499	-1.098328	-1.998817
C	4.400072	-1.133412	-2.253784
H	2.323106	-0.135113	-2.181632
H	3.537160	1.866625	-0.960329
H	5.920062	1.937301	-0.507424
H	3.970433	-1.986614	-2.753318
C	-1.840830	-1.224434	-3.684973
H	-2.302235	-0.587884	-4.435958
H	-0.783696	-0.988228	-3.621506
H	-1.953427	-2.252438	-4.018570
C	-5.061214	-1.020481	0.291754
H	-5.923861	-1.399284	-0.242938
H	-4.861953	-1.684011	1.128573
H	-5.312118	-0.044202	0.696259
C	0.749259	3.569311	-0.794349
H	0.360887	4.388619	-1.391861
H	1.545894	3.969355	-0.172004
H	1.168325	2.825882	-1.464352
C	-3.155952	2.727444	2.400492
H	-3.275006	3.761531	2.699949
H	-4.088812	2.389989	1.958607
H	-2.974251	2.130714	3.288967
C	2.272150	-2.225969	0.914694
H	2.350942	-3.303066	0.797108
H	2.454718	-1.756572	-0.043706
H	3.052309	-1.915974	1.604993
C	-2.033539	-1.827623	3.638009
H	-2.262887	-0.873772	4.103214
H	-2.945628	-2.202698	3.183166
H	-1.728957	-2.521598	4.411881
Br	0.961768	-3.575566	3.804990
Br	-5.167818	-1.999716	-3.041856
Br	-1.309443	5.444210	1.258811
H	6.363357	-1.932233	-2.311177
N	7.704841	0.021387	-1.065639
C	8.561832	-0.955158	-1.692435

H	8.540595	-0.905888	-2.784268
H	9.581632	-0.792717	-1.365659
H	8.285617	-1.963807	-1.396191
C	8.307043	1.265180	-0.652919
H	7.873200	1.618066	0.279218
H	9.363377	1.105235	-0.474726
H	8.202307	2.059736	-1.396515

T-I4

Cu	0.076295	0.146397	-1.018006
B	-2.104102	0.107370	1.126133
H	-2.930208	0.128359	1.977331
C	-3.967422	0.923549	-0.526770
C	-3.963132	1.237901	-1.870200
C	-2.645235	1.089272	-2.318839
N	-1.907238	0.712720	-1.292776
N	-2.701611	0.609616	-0.204792
N	-0.531629	-1.573569	0.109966
C	-0.279559	-2.867972	0.130682
C	-1.178102	-3.477153	1.013451
C	-1.980640	-2.474912	1.517505
N	-1.563132	-1.330808	0.950698
N	0.130158	1.168386	0.686251
C	0.998949	2.000126	1.235721
C	0.480486	2.409713	2.465260
C	-0.739390	1.778277	2.615647
N	-0.924397	1.031734	1.518322
O	0.765464	-0.227347	-2.645307
C	3.733712	-0.880216	-0.941195
C	4.695276	-1.281677	-0.053305
C	6.017182	-0.969189	-0.346192
C	6.355800	-0.272752	-1.517176
C	5.316763	0.122827	-2.374524
C	3.990929	-0.183615	-2.093277
H	1.117713	-1.107226	-2.735193
H	4.456337	-1.820584	0.849355
H	6.780138	-1.275949	0.346063
H	3.193864	0.122696	-2.751260
C	-2.071844	1.294653	-3.675055
H	-2.032833	2.353730	-3.919969
H	-1.070763	0.878141	-3.719063
H	-2.692457	0.812098	-4.424506
C	-5.099339	0.905855	0.438084
H	-6.003124	1.239930	-0.056922
H	-5.274277	-0.093786	0.825758
H	-4.909584	1.560343	1.283481
C	2.277770	2.381023	0.581254
H	2.283153	3.440653	0.339576
H	3.117147	2.190991	1.243376
H	2.434486	1.814899	-0.330049
C	-1.711509	1.857434	3.738185
H	-1.330163	2.524366	4.501702
H	-2.672196	2.235177	3.401516
H	-1.876440	0.882147	4.186038
C	0.793825	-3.490120	-0.689518
H	0.380629	-3.952595	-1.583080
H	1.526123	-2.748021	-0.989049
H	1.302865	-4.266103	-0.126838
C	-3.099751	-2.562442	2.493685
H	-2.908047	-1.954753	3.372943
H	-4.034940	-2.225610	2.055844
H	-3.224595	-3.590954	2.809904
Br	-1.260811	-5.305578	1.425756
Br	-5.431016	1.767856	-2.910855
Br	1.299965	3.592344	3.665900
H	5.531850	0.673438	-3.272333

N	7.680349	0.005224	-1.823798
C	7.964434	0.952769	-2.873569
H	7.547513	1.944998	-2.680725
H	9.037809	1.051232	-2.980387
H	7.576794	0.605270	-3.827542
C	8.679474	-0.163085	-0.797562
H	8.736882	-1.199595	-0.475185
H	9.649319	0.101013	-1.201084
H	8.496844	0.455622	0.085363

T-TS3

Cu	0.163139	0.089710	-1.310197
B	-1.337388	-0.115008	1.383024
H	-1.930984	-0.134316	2.408879
C	-3.603956	-0.753902	0.262106
C	-4.008659	-0.897106	-1.047988
C	-2.892500	-0.636069	-1.850220
N	-1.873268	-0.354360	-1.055718
N	-2.303898	-0.422092	0.223811
N	0.662195	-1.252340	0.388502
C	1.537242	-2.194118	0.695342
C	1.228363	-2.693438	1.965608
C	0.121675	-2.002407	2.405718
N	-0.196480	-1.137855	1.427935
N	-0.145197	1.639304	-0.002255
C	0.099270	2.944633	0.030826
C	-0.345328	3.441899	1.256159
C	-0.885133	2.376250	1.947594
N	-0.754436	1.302759	1.159459
O	0.346174	-0.963141	-2.905893
C	1.870954	-0.174000	-3.397705
C	2.938207	-1.082798	-3.545151
C	4.113265	-0.869764	-2.875340
C	4.291503	0.216940	-1.986682
C	3.251593	1.107295	-1.848294
C	2.029878	0.928142	-2.536183
H	0.482314	-1.900876	-2.764024
H	2.846724	-1.925373	-4.214092
H	3.371137	1.976475	-1.222481
H	1.373878	1.780204	-2.642907
Br	-0.243951	5.218633	1.848365
Br	2.157745	-4.042648	2.879672
Br	-5.725203	-1.345642	-1.656606
C	-2.805293	-0.628572	-3.334825
H	-3.321766	-1.491262	-3.745131
H	-3.289338	0.257998	-3.738980
H	-1.770219	-0.649340	-3.650927
C	-4.386265	-0.921347	1.516159
H	-5.405057	-1.197207	1.272529
H	-3.963774	-1.698958	2.145641
H	-4.410220	-0.002865	2.095078
C	0.701083	3.723811	-1.085838
H	0.574867	4.782590	-0.894417
H	1.763694	3.531373	-1.190984
H	0.216534	3.494903	-2.029251
C	-1.511138	2.352803	3.296956
H	-1.415088	3.329028	3.756189
H	-2.566975	2.105430	3.239233
H	-1.033259	1.621620	3.940900
C	2.647940	-2.604461	-0.199755
H	3.605377	-2.262943	0.185705
H	2.694402	-3.687288	-0.274522
H	2.523986	-2.186267	-1.188833
C	-0.624398	-2.132269	3.686365
H	-0.165389	-2.902376	4.294444
H	-0.614884	-1.203093	4.248558

H	-1.661746	-2.405380	3.516894
H	4.931755	-1.561007	-3.007464
N	5.520815	0.313757	-1.282785
C	6.679120	0.556190	-2.121249
H	6.702778	1.576650	-2.520822
H	7.585310	0.396125	-1.542840
H	6.693667	-0.130056	-2.959860
C	5.520997	1.163933	-0.118749
H	4.685869	0.911210	0.526801
H	6.441105	1.003355	0.436501
H	5.462535	2.234132	-0.353930

T-I5

Cu	0.069641	-0.264864	-1.246722
B	-1.325693	0.275646	1.445597
H	-1.891728	0.453664	2.472434
C	-3.586449	-0.694468	0.544336
C	-3.972674	-1.229749	-0.669325
C	-2.850262	-1.203576	-1.499058
N	-1.849151	-0.679045	-0.810913
N	-2.291796	-0.370166	0.425654
N	0.600911	-1.119272	0.616706
C	1.533722	-1.930803	1.082906
C	1.396462	-2.002515	2.473226
C	0.331672	-1.196095	2.812605
N	-0.132705	-0.674436	1.665738
N	-0.256667	1.689342	-0.340768
C	0.034457	2.954950	-0.584415
C	-0.371149	3.722313	0.512292
C	-0.924535	2.850052	1.424538
N	-0.841653	1.625906	0.878115
O	0.602506	-1.719028	-4.018606
C	1.743959	-1.331833	-3.376027
C	2.903305	-2.028107	-3.442119
C	4.060434	-1.574152	-2.762485
C	4.017419	-0.367250	-2.017195
C	2.853518	0.347815	-1.957659
C	1.585122	-0.102381	-2.587253
H	0.780274	-2.495134	-4.545277
H	2.948794	-2.950268	-4.005129
H	2.819912	1.279715	-1.421055
H	1.179302	0.675534	-3.244980
Br	-0.194398	5.581747	0.684940
Br	2.462762	-3.015746	3.636961
Br	-5.669931	-1.873652	-1.134981
C	-2.719107	-1.654177	-2.909556
H	-3.061960	-2.680106	-3.011253
H	-3.334623	-1.041630	-3.563612
H	-1.689965	-1.597165	-3.247045
C	-4.384213	-0.486024	1.781966
H	-5.397770	-0.833330	1.622861
H	-3.961907	-1.032522	2.619749
H	-4.421085	0.564286	2.055071
C	0.677031	3.425063	-1.840036
H	0.101625	4.234866	-2.279463
H	1.676136	3.806607	-1.645478
H	0.751573	2.621069	-2.559963
C	-1.515069	3.132944	2.760336
H	-1.446134	4.193840	2.968092
H	-2.561194	2.844297	2.801720
H	-0.992514	2.597197	3.547180
C	2.520976	-2.622531	0.216722
H	3.511157	-2.190524	0.331846
H	2.584251	-3.672708	0.487184
H	2.245530	-2.541421	-0.826580
C	-0.246386	-0.913288	4.153796

H	0.318847	-1.444902	4.909610
H	-0.211576	0.147122	4.385333
H	-1.282789	-1.231882	4.214914
H	4.948783	-2.180020	-2.759281
N	5.191582	0.013290	-1.319454
C	6.372030	0.187814	-2.141657
H	6.335695	1.108665	-2.735329
H	7.253150	0.229908	-1.506461
H	6.490643	-0.643069	-2.825186
C	5.058289	1.073657	-0.353002
H	4.240240	0.859111	0.326552
H	5.974898	1.138001	0.226948
H	4.881151	2.058331	-0.802966

MECP2

Cu	-0.00776294	-0.23714568	-1.29309855
B	-1.36930345	0.27859143	1.41724382
H	-1.92469188	0.46460962	2.44789885
C	-3.63439887	-0.70103044	0.53870028
C	-4.03311521	-1.22714778	-0.67586495
C	-2.92474009	-1.17602055	-1.52243188
N	-1.91907000	-0.64636629	-0.84247685
N	-2.34747542	-0.35738770	0.40390752
N	0.52433893	-1.14709388	0.56544805
C	1.46846262	-1.94934705	1.02562986
C	1.35999263	-1.99687753	2.42013160
C	0.30417368	-1.18410804	2.76910572
N	-0.18538637	-0.68254396	1.62375544
N	-0.31546600	1.67514032	-0.39102596
C	0.00860830	2.93276563	-0.63799722
C	-0.35880587	3.70608022	0.46781266
C	-0.92012702	2.84584906	1.38788605
N	-0.88111851	1.62238200	0.83786513
O	0.55268696	-1.92089946	-3.55705943
C	1.76472350	-1.38203179	-3.18888317
C	2.95258205	-1.94699887	-3.46745813
C	4.15885676	-1.38597196	-2.95342712
C	4.09498083	-0.29958729	-2.02188425
C	2.89659202	0.27844886	-1.73402984
C	1.60756075	-0.10253743	-2.43125853
H	0.68949270	-2.75216646	-4.00698097
H	3.00416470	-2.86636184	-4.03524025
H	2.83572326	1.06662528	-1.00510989
H	1.42476610	0.66100362	-3.20640109
Br	-0.12813995	5.55841669	0.64415266
Br	2.45002710	-2.99061922	3.57802072
Br	-5.72921900	-1.88529338	-1.12313886
C	-2.81232030	-1.60466903	-2.94124744
H	-3.15614573	-2.62915768	-3.05136036
H	-3.43727108	-0.98263667	-3.57719916
H	-1.78741255	-1.54652081	-3.28929936
C	-4.40968265	-0.51741506	1.79495897
H	-5.42399222	-0.87094789	1.65471845
H	-3.96408615	-1.07053934	2.61642765
H	-4.44847935	0.52898628	2.08208504
C	0.64814935	3.38317335	-1.90215759
H	0.08724948	4.20459052	-2.33888037
H	1.65769997	3.73993290	-1.71653796
H	0.70114725	2.57363100	-2.61761834
C	-1.47959218	3.13311165	2.73634819
H	-1.40017816	4.19269077	2.94594477
H	-2.52591134	2.84896932	2.79872690
H	-0.94363665	2.59144172	3.51036525
C	2.44706229	-2.65986342	0.16363847
H	3.44083390	-2.23723521	0.27868597
H	2.49735759	-3.70740670	0.44681713

H	2.18112551	-2.58482994	-0.88232837
C	-0.24601722	-0.87799623	4.11666724
H	0.32916627	-1.40421835	4.86863594
H	-0.19798997	0.18510763	4.33310803
H	-1.28372719	-1.18693227	4.20134168
H	5.08479052	-1.90440152	-3.11960887
N	5.29501357	0.04252601	-1.34673858
C	6.44334018	0.29868924	-2.19026699
H	6.36180550	1.24216663	-2.74212841
H	7.33682700	0.34396380	-1.57296169
H	6.58140412	-0.49546515	-2.91199563
C	5.18504366	1.01531086	-0.29102053
H	4.43474590	0.70418697	0.42805699
H	6.13840288	1.08616479	0.22472110
H	4.91903804	2.01813941	-0.64805122

S-I6

Cu	0.481662	-0.015514	-0.564853
B	-2.201276	0.037155	0.703179
H	-3.266656	0.052610	1.226367
C	-2.714689	2.523724	0.099688
C	-2.027528	3.500715	-0.587656
C	-0.817276	2.927580	-0.996158
N	-0.782670	1.679483	-0.577217
N	-1.930929	1.429684	0.087951
N	-1.104531	-1.302527	-1.137113
C	-1.392111	-2.271899	-1.980982
C	-2.724926	-2.651816	-1.789261
C	-3.218490	-1.855420	-0.778286
N	-2.209594	-1.047308	-0.404157
N	0.179161	-0.396603	1.448917
C	0.844995	-0.718015	2.539729
C	-0.063326	-0.830354	3.598210
C	-1.310834	-0.561204	3.078898
N	-1.131115	-0.301658	1.770958
O	2.137743	0.130211	-1.929267
C	3.491980	0.025731	-1.693304
C	4.311215	-0.751094	-2.493429
C	5.662342	-0.834561	-2.220076
C	6.233563	-0.141051	-1.149160
C	5.382973	0.634919	-0.360366
C	4.024825	0.716275	-0.625646
H	1.918712	-0.207107	-2.794088
H	3.897213	-1.307851	-3.320553
H	5.773032	1.197871	0.468084
H	3.381794	1.326413	-0.013174
Br	0.358634	-1.266792	5.375491
Br	-3.641987	-3.991093	-2.734299
Br	-2.587203	5.262285	-0.922503
C	0.296593	3.538358	-1.768969
H	-0.051667	3.905314	-2.731073
H	0.723751	4.384387	-1.236452
H	1.076266	2.803472	-1.937096
C	-4.051676	2.592457	0.748676
H	-4.466597	3.584803	0.617897
H	-4.741871	1.874537	0.315245
H	-3.987731	2.386985	1.813174
C	2.318180	-0.915170	2.552535
H	2.799435	-0.214720	3.230634
H	2.572012	-1.916936	2.889279
H	2.726016	-0.771788	1.558328
C	-2.630298	-0.544316	3.766042
H	-2.493458	-0.775457	4.815697
H	-3.106012	0.428913	3.689902
H	-3.309448	-1.277666	3.341273
C	-0.395466	-2.810738	-2.944049

H	-0.317632	-3.891145	-2.861916
H	-0.673061	-2.582460	-3.970476
H	0.581367	-2.383771	-2.740538
C	-4.577142	-1.840131	-0.172229
H	-5.198535	-2.581915	-0.659691
H	-4.541681	-2.068887	0.888848
H	-5.052107	-0.870026	-0.284679
H	6.275285	-1.472561	-2.832691
N	7.602999	-0.275637	-0.875524
C	8.519308	-0.070557	-1.979597
H	8.632314	0.987227	-2.238133
H	9.496195	-0.460675	-1.710123
H	8.189315	-0.598136	-2.864691
C	8.089738	0.291696	0.357654
H	7.490591	-0.054147	1.193418
H	9.109030	-0.045058	0.517831
H	8.094853	1.387412	0.363973
